# Evaluating quality of colorectal cancer surgery across elective and emergency health systems: secondary analysis of prospective cohort studies in 95 countries

**DOI:** 10.1093/bjsopen/zrag062

**Published:** 2026-07-22

**Authors:** Sivesh K Kamarajah, Adewale O Adisa, Ajay Aggarwal, Alazar Berhe, James Glasbey, Ewen Harrison, Parvez D Haque, Ismail Lawani, Sonia Mathai, Ana Minaya Bravo, C S Pramesh, April C Roslani, Chris Varghese, Aneel Bhangu

**Affiliations:** NIHR Global Health Research Unit on Global Surgery, School of Health Sciences, University of Birmingham, Birmingham, UK; Obafemi Awolowo University Teaching Hospital Complex, Ife, Osun, Nigeria; Faculty of Public health and policy, London School of Hygiene and Tropical Medicine, London, UK; Department of Oncology, Guy's & ST Thomas' NHS Trust, London UK; Hawassa University College of Medicine and Health Sciences, Ethiopia; NIHR Global Health Research Unit on Global Surgery, School of Health Sciences, University of Birmingham, Birmingham, UK; Department of HPB Surgery, NHS Lothian, Edinburgh, UK; Christian Medical College, Ludhiana, India; International Hospital of Calavi (CHIC), Cotonou, Benin; Tata Medical Center, Kolkata, India; General Surgery Department, Hospital Universitario HM Rivas, Rivas-VaciaMadrid, Madrid, Spain; Tata Memorial Hospital, Homi Bhabha National Institute, Mumbai, India; National Cancer Grid, Tata Memorial Hospital, Mumbai, India; Faculty of Medicine, Universiti Malaya, Kuala Lumpur, Malaysia; University Malaya Medical Centre, Kuala Lumpur, Malaysia; Department of Surgery, University of Auckland, Auckland, New Zealand; NIHR Global Health Research Unit on Global Surgery, School of Health Sciences, University of Birmingham, Birmingham, UK

**Keywords:** global health

## Abstract

**Background:**

The Lancet Oncology Commission on Global Cancer Surgery recommended that access to and the quality of surgical care be improved. This study aimed to understand differences in surgical quality between emergency and elective resection among patients with potentially curative colorectal cancer.

**Methods:**

This preplanned secondary analysis included patients undergoing only curative-intent surgery for colorectal cancer from three contemporary global prospective cohort studies (GlobalSurg-3, 5506 patients; CovidSurg-Cancer, 6719 patients; APOLLO, 876 patients) registered from 2018 to 2023. Hierarchical multilevel logistic regression models quantified associations between the urgency of surgery (elective *versus* emergency) and surgical quality, measured by margin-positive resection, adjusting for patient, disease, and health system factors. Bootstrap multivariable simulations evaluated effect modification by country income level, cancer stage, and location.

**Results:**

Of the 45 699 patients registered, 13 101 across 95 countries were included in this analysis. Overall, 678 patients (5.4%) had margin-positive resections, with higher rates in the emergency than elective surgery group (13.7 *versus* 4.5%; *P* < 0.0001). In adjusted multilevel models, emergency surgery was associated with increased odds of margin-positive resections (adjusted odds ratio 2.45, 95% confidence interval (c.i.) 1.86 to 3.22), consistent across all country income groups and robust to alternative health system indicators. Bootstrap-derived absolute risk differences revealed the greatest disparities in patients with stage III–IV rectal cancers, with absolute differences of 12.6% (95% c.i. 10.2 to 15.7) in high-income countries, 19.0% (95% c.i. 13.6 to 23.9) in upper middle-income countries, and 14.1% (95% c.i. 10.9 to 16.5) in lower middle- and low-income countries. Variance decomposition demonstrated that hospital- and country-level factors accounted for 76% of the explained variation in surgical quality.

**Conclusion:**

Emergency surgery was associated with a two- to threefold increase in the risk of margin-positive resections globally, independent of resource availability, highlighting a neglected area of global surgical practice. These findings challenge the assumption that poorer outcomes after emergency surgery are due to advanced disease stage. For patients presenting as an emergency with potentially curative resection, enhanced decision-making around resectability, ensuring specialist surgeon availability, and developing bridge-to-surgery pathways represent immediate, low-cost strategies to improve global cancer outcomes.

## Teaser

Emergency surgery conferred a two- to threefold increased risk of margin-positive resections globally, independent of resource availability, exposing a neglected area of global surgical practice. The findings challenge the assumption that poorer outcomes after emergency surgery are primarily due to advanced disease stage. For patients who present as an emergency with potentially curative disease, enhanced decision-making around resectability, ensuring specialist surgeon availability, and developing bridge-to-surgery pathways represent immediate, low-cost strategies to improve global cancer outcomes.

## Introduction

Colorectal cancer is the world’s third most common malignancy and the second leading cause of cancer-related death^1,2^, driven by shifting demographics, urbanization, and lifestyles^3^. Up to 30% of patients present as an emergency, often due to obstruction, requiring urgent surgery^4^. Unlike many other cancers, a substantial proportion of patients with colorectal cancer still have potentially curable disease. The recent Lancet Oncology Commission on Global Cancer Surgery^5^ noted the urgent need to improve both access to and the quality of surgical care, noting that surgery remains the primary curative modality in up to 80% of cancer cases^2,6^. Although improved access through screening, early diagnosis, and elective surgery is ideal, this requires long-term investment in infrastructure and workforce, often hindered by global health funding cliffs and competing priorities. In contrast, improving the quality of emergency cancer surgery through better frontline decision-making, specialist availability, and systems-level support offers a more immediate, cost-effective route to better outcomes.

The quality of cancer surgery in both elective and emergency settings critically affects recurrence, long-term survival, functional outcomes, and healthcare costs^7^. Quality can be assessed objectively using established indicators such as margin-positive resection and lymph node yield^7,8^. Despite calls to monitor and improve these metrics, global data, particularly in emergency contexts, remain sparse and fragmented^9,10^. Further, worse outcomes in emergency colorectal surgery are often attributed to late-stage disease at presentation^11^, with less focus on potentially modifiable factors, such as surgical decision-making and operator expertise. However, even in advanced-stage disease, long-term outcomes can be improved through higher-quality surgery. Disentangling the impact of disease stage from that of surgical quality is therefore essential to identify actionable, system-level interventions that can improve global cancer care.

To address this critical knowledge gap, the present study, a comprehensive global assessment of surgical quality in patients with potentially curative colorectal cancer, was conducted, comparing elective and emergency settings using data from three international prospective cohort studies. The focus was on margin-positive resection as the primary indicator of quality because it is directly modifiable, measurable across surgical contexts, reflects technical and decision-making expertise, and is strongly linked to long-term outcomes. The primary aims of this study were to quantify differences in margin-positive resection between emergency and elective surgery and to determine whether these differences persist after adjusting for patient, tumour, country income, and health system factors.

## Methods

### Data source, study design and participants

This was a preplanned secondary analysis of three large global prospective cohort studies, namely GlobalSurg-3 (15 958 patients, 428 hospitals, 82 countries)^12^, CovidSurg-Cancer (20 006 patients, 466 hospitals, 61 countries)^13,14^, and APOLLO (1861 patients, 466 hospitals, 61 countries)^15^, registered between 2018 and 2023. These studies collectively provide the most comprehensive international data sets for evaluating surgical quality in potentially curative colorectal cancer across elective and emergency settings. They were selected for their recent, harmonized data collection, broad representation of different health system settings, and consistent capture of clinically relevant variables. Primary results of these individual studies have been reported elsewhere^12–14^, and the detailed methodology for each original cohort study, including specifics on data collection periods, inclusion criteria, and follow-up processes, is provided fully in the *Supplementary material*. All data collection systems were designed specifically for this study, building upon existing collaborative research networks globally (*Supplementary material*). All participating centres obtained ethics approval or an exemption in accordance with local regulations. Informed consent was not required for these observational studies using routinely collected data. This secondary analysis included adult patients (age ≥ 18 years) undergoing curative-intent colorectal cancer surgery across these cohorts. Patients undergoing palliative surgery were excluded from the present analysis.

This study is reported in accordance with the STROBE statement for observational studies^16^ (*Supplementary material*).

### Study exposure and variables

The primary exposure in this study is the urgency of surgery, defined pragmatically as the time between the decision to operate and skin incision, following the National Confidential Enquiry into Patient Outcome and Death (NCEPOD) nomenclature^17^. Therefore, procedures performed ≤ 48 hours (h) after the decision point were defined as emergency surgeries, whereas those undertaken > 48 h were defined as elective surgeries. This timing-based definition is widely used in contemporary global cohort studies (for example, GlobalSurg-3^12^, CovidSurg-Cancer^13,14^) and offers a reproducible, auditable exposure that can be captured across heterogeneous health systems. Although time is the operational criterion, the clinical substrate of emergency surgery is inherently unplanned: decisions are driven by acute presentations, such as large bowel obstruction, impending perforation, or uncontrolled haemorrhage in otherwise potentially curable disease. Where surgeons deemed that short-term decompression (for example, loop stoma, stenting) could safely bridge to surgery, those patients entered the elective category because definitive resection occurred after the 48-h window. Thus, the exposure captures real-world surgical decision-making rather than preconceived intent. Precise timestamps beyond the 48-h dichotomy (for example, 72 h or 7 days) were not consistently available, precluding formal threshold robustness analyses. Nevertheless, contemporary studies show that scheduled elective colorectal resections typically occur weeks after multidisciplinary review, well beyond the emergency window, limiting the risk of systematic misclassification.

To evaluate whether the association between urgency and quality of cancer surgery across health systems is consistent across health systems, as a secondary analysis, a concise panel of macro-level secondary exposures that directly addresses policy-relevant disparities highlighted by the Lancet Oncology Commission on Global Cancer Surgery^5^ was identified. These macro-level indicators were the World Bank income group^18^, Healthcare Access and Quality^19^, Universal Health Coverage index^20^, specialist surgical workforce density^21^, health expenditure per capita (purchasing power parity in US$; World Bank), and the human development index (HDI)^2^. A detailed description of these indicators is provided in the *Supplementary material*. To account for case mix differences, clinically relevant variables were age at surgery, sex, body mass index (defined according to World Health Organization categories), American Society of Anesthesiologists (ASA)physical status (grades I–V), American Joint Commission on Cancer tumour stage, cancer location (colon or rectum), and surgical approach (open or minimally invasive surgery).

### Outcomes of interest

The primary outcome was margin-positive resection because it represents a directly modifiable, pathologically verifiable, and internationally standardized indicator of surgical oncological quality^7,8^. Margin-positive resection was defined as microscopic tumour at or within 1 mm of a surgical margin (R1) or macroscopic residual tumour (R2). Margin status was classified according to the international pathology guidelines issued by The Royal College of Pathologists^22–24^. For rectal cancers, circumferential resection margins ≤ 1 mm were defined as margin positive, consistent with European Society for Medical Oncology standards^23^. These guidelines can be consistently applied across diverse global settings. Margin status was assessed by consultant gastrointestinal pathologists at each site using final pathology reports. Interobserver reproducibility of margin assessment has been demonstrated to be substantial; for example, in comparative studies^25^, the agreement among experienced gastrointestinal pathologists for margin status was κ = 0.67. Further, margin positivity has been validated as a surrogate marker for local recurrence and cancer-specific survival, independently of stage. For example, cohort studies^26,27^ have shown that margin positivity confers a more than two to threefold increased risk of recurrence and death, even after multivariable adjustment. Mortality outcomes such as 90- or 365-day mortality and recurrence data were not uniformly available across all three cohort data sets. Moreover, mortality is highly susceptible to postoperative system-level factors, such as critical care infrastructure or adjuvant therapy access, which vary markedly between countries. Margin positivity avoids these confounders by focusing on intraoperative decision-making and surgical technique, making it a more globally comparable and actionable quality metric^7,8^.

### Statistical analysis

This analysis leverages three existing global cohorts and was therefore not prospectively powered. Nonetheless, an *a priori* sample-size calculation was conducted to ensure that the combined data sets would be sufficient to detect clinically important differences in the primary outcome between emergency and elective surgery. Contemporary national audits from high-income settings report margin-positive rates of 5–8% after elective colorectal resection, whereas rates for emergency procedures consistently exceed 10%^10^. Assuming a conservative absolute difference of 5% between elective and emergency procedures (that is, 10 *versus* 15%), a two-sided χ^2^ test with α = 0.05 and 80% power requires approximately 685 patients per group. In a more stringent scenario of a 3% absolute difference (5 *versus* 8%), the required sample is approximately 1060 patients per group.

Categorical data are reported as counts and percentages and were compared using χ^2^ tests. Continuous data are summarized as the median with interquartile range or as the mean and standard deviation and were compared using the Mann-Whitney *U* test, Student’s *t* test, or analysis of variance (ANOVA), as appropriate. Baseline characteristics were compared between patients undergoing elective and emergency surgery, between patients with and without margin-positive resections, and between patients who received and did not receive adjuvant chemotherapy. A detailed description of the statistical methodology, including handling of missing data, is presented in the *Supplementary material*. Briefly, a complete-case analysis was preplanned if missing data were minimal (< 5%) and missing at random. For missingness exceeding 5%, multiple imputation by chained equations was planned, assuming data were missing at random or completely at random.

For the primary analysis, the association between the urgency of surgery (primary exposure) and positive resection margin (primary outcome) was evaluated with hierarchical multilevel logistic regression models (patients nested within hospitals, hospitals within countries) to account for clustering and unmeasured contextual effects, specifying random intercepts at hospital and country levels. Clinically plausible patient and operative-related factors were selected *a priori* for inclusion as fixed effects in adjusted analyses by clinical experts within the study management group. The factors included in the models were World Bank income group, age, sex, body mass index category, ASA grade, American Joint Commission on Cancer tumour stage, cancer site, and operative approach (open or minimally invasive). All patients had the same follow-up period of up to 30 days after surgery. To explore potential effect modification by key clinical characteristics, bootstrap simulations (2000 iterations) were conducted using multivariable models that incorporated prespecified interactions among urgency, macro-level indicators, preoperative overall stage, and cancer site. These interaction terms were selected *a priori* based on existing literature suggesting that stage of presentation and cancer site may modulate margin-positive resection^28^. The primary analyses are further detailed in the *Supplementary material*. Sensitivity analyses were undertaken to include patients undergoing surgery in the non-pandemic cohorts. Three secondary analyses were conducted. First, the robustness of the primary analysis was evaluated by replacing income group with alternative macro-level indicators as described above, each categorized into tertiles. For each indicator the adjusted odds ratio (aOR) is reported for emergency *versus* elective surgery and the macro-level indicators. Second, a variance decomposition analysis was conducted to estimate the proportion of total explained variation attributable to patient-level *versus* system-level characteristics. Conditional and marginal pseudo-*R*^2^ values were calculated based on fixed and random effects. Following the approach of Nakagawa and Schielzeth (2013)^29^ and the nested-effects extension by Johnson (2014)^30^, marginal *R*^2^ reflects the variance explained by the fixed (patient-level) covariates alone, whereas conditional *R*^2^ reflects variance explained by both fixed effects and the random intercepts for hospital, country, and World Bank income group. The gap between the two therefore quantifies the contribution of system-level factors. Third, the association between surgical urgency and the receipt of adjuvant chemotherapy among eligible patients was evaluated using multilevel logistic regression models with prespecified clinical covariates. The secondary analyses are further detailed in the *Supplementary material*. Statistical significance was defined as *P* < 0.05 two-tailed. All analyses were performed using R version 4.3.3^®^ (R Foundation Statistical Computing, Vienna, Austria) with finalfit, dplyr, and ggplot2 packages.

## Results

### Cohort characteristics

**Fig. 1:**
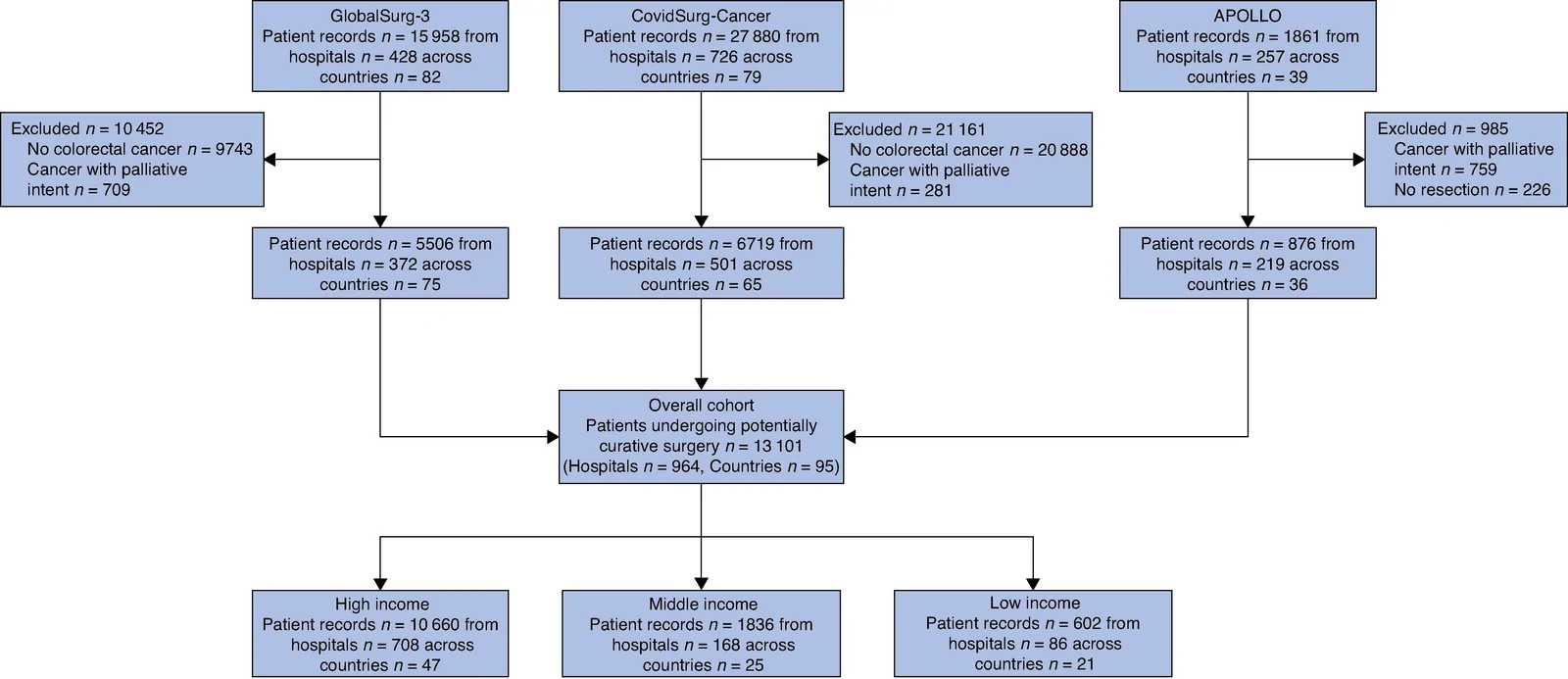
Flow chart of patient inclusion across the three prospective cohort studies

Of 45 699 patients registered, this study included 13 101 patients with potentially curative colorectal cancers across 964 hospitals and 95 countries: 5506 from GlobalSurg-3, 6719 from CovidSurg-Cancer, and 876 from APOLLO (*Fig. 1* and *Table S1*). Of these patients, 10 121 (77.3%) were from high-income countries, 1618 (12.4%) were from upper middle-income countries, and 1351 (10.3%) were from lower middle- or low-income countries. A summary flow diagram is presented in *Fig. 1*. Most patients in the cohort were aged 70–79 years (3788, 28.9%), and 7412 (56.6%) were male. Patients undergoing potentially curative colorectal cancer surgery were more likely to be older, have colon cancer with preoperative overall stage I or II and open surgery in APOLLO compared with CovidSurg-Cancer or GlobalSurg-3. Baseline characteristics across the three studies are presented in *Tables S2* and *S3*. Because the overall missing rate was < 5% across the data sets (*Fig. S2* and *Table S10*), a complete case analysis was performed.

### Associations between urgency of surgery and cohort characteristics

In the present cohort, 1366 patients (10.4%) underwent emergency cancer surgery, which was more common than elective surgery among patients from upper middle-income (19.5 *versus* 11.5%) and lower middle- or low-income (12.8 *versus* 10.0%, *P* < 0.0001) countries (*Table 1*). Patients undergoing emergency cancer surgery were more likely to be younger (< 50 years: 13.5 *versus* 10.7%; *P* < 0.0001), ASA grade I (16.3 *versus* 13.7%; *P* < 0.0001), and be classified as normal weight (43.6 *versus* 40.6%, *P* < 0.0001) than patients undergoing elective surgery (*Table 2*). Patients undergoing emergency surgery were more likely to have colon cancer (90.3 *versus* 66.6%; *P* < 0.0001), undergo open surgery (82.3 *versus* 41.3%; *P* < 0.0001), and have stage III or IV cancers (49.0 *versus* 42.5%; *P* < 0.0001) than those undergoing elective surgery (*Table 2*). A comparison of patient- and operative-level characteristics between the elective and emergency surgery groups is presented in *Table 2*.

**Table 1:** Health systems characteristics for patients undergoing potentially curative colorectal cancer surgery overall and stratified by urgency of surgery

	Elective	Emergency	Total	*P*
No. of patients	11 726 (89.6%)	1366 (10.4%)	13 092	
**Country income level**				
High income	9197 (78.4%)	924 (67.6%)	10 121 (77.3%)	< 0.001
Upper middle income	1351 (11.5%)	267 (19.5%)	1618 (12.4%)	
Lower middle or low income	1176 (10.0%)	175 (12.8%)	1351 (10.3%)	
Missing	2 (0.0%)	0 (0.0%)	2 (0.0%)	
**HAQ index**				
High	4899 (41.8%)	430 (31.5%)	5329 (40.7%)	< 0.001
Middle	3798 (32.4%)	434 (31.8%)	4232 (32.3%)	
Low	2890 (24.6%)	497 (36.4%)	3387 (25.9%)	
Missing	139 (1.2%)	5 (0.4%)	144 (1.1%)	
**Surgical workforce**				
High	3899 (33.3%)	494 (36.2%)	4393 (33.6%)	< 0.001
Middle	4466 (38.1%)	358 (26.2%)	4824 (36.8%)	
Low	2964 (25.3%)	402 (29.4%)	3366 (25.7%)	
Missing	397 (3.4%)	112 (8.2%)	509 (3.9%)	
**HDI tertile**				
High	9661 (82.4%)	995 (72.8%)	10 656 (81.4%)	< 0.001
Middle	1558 (13.3%)	273 (20.0%)	1831 (14.0%)	
Low	505 (4.3%)	98 (7.2%)	603 (4.6%)	
Missing	2 (0.0%)	0 (0.0%)	2 (0.0%)	
**Health expenditure per PPP**				
High	3472 (29.6%)	319 (23.4%)	3791 (29.0%)	< 0.001
Middle	5130 (43.7%)	548 (40.1%)	5678 (43.4%)	
Low	2985 (25.5%)	494 (36.2%)	3479 (26.6%)	
Missing	139 (1.2%)	5 (0.4%)	144 (1.1%)	
**Universal health coverage**				
High	3806 (32.5%)	411 (30.1%)	4217 (32.2%)	< 0.001
Middle	4660 (39.7%)	390 (28.6%)	5050 (38.6%)	
Low	3085 (26.3%)	542 (39.7%)	3627 (27.7%)	
Missing	175 (1.5%)	23 (1.7%)	198 (1.5%)	

Values are *n* (%). HAQ, healthcare access and quality; HDI, human development index; PPP, power purchase parity.

**Table 2:** Patient and procedural characteristics for patients undergoing potentially curative colorectal cancer surgery overall and stratified by the urgency of surgery

	Elective	Emergency	Total	*P*
No. of patients	11 726 (89.6%)	1366 (10.4%)	13 092	
**Age group**				
< 50 years	1250 (10.7%)	185 (13.5%)	1435 (11.0%)	< 0.001
50–59 years	1986 (16.9%)	241 (17.6%)	2227 (17.0%)	
60–69 years	3265 (27.8%)	310 (22.7%)	3575 (27.3%)	
70–79 years	3433 (29.3%)	355 (26.0%)	3788 (28.9%)	
> 80 years	1792 (15.3%)	275 (20.1%)	2067 (15.8%)	
Missing	0 (0.0%)	0 (0.0%)	0 (0.0%)	
**Sex**				
Female	5061 (43.2%)	613 (44.9%)	5674 (43.4%)	0.23
Male	6660 (56.8%)	752 (55.1%)	7412 (56.6%)	
**ASA physical status**				
Grade I	1601 (13.7%)	223 (16.3%)	1824 (13.9%)	< 0.001
Grade II	6117 (52.2%)	573 (41.9%)	6690 (51.1%)	
Grade III	3588 (30.6%)	431 (31.6%)	4019 (30.7%)	
Grade IV	289 (2.5%)	79 (5.8%)	368 (2.8%)	
Grade V	5 (0.0%)	29 (2.1%)	34 (0.3%)	
Missing	126 (1.1%)	31 (2.3%)	157 (1.2%)	
**Body mass index**				
< 18.5 kg/m^2^ (underweight)	412 (3.5%)	88 (6.4%)	500 (3.8%)	< 0.001
18.5–24.9 kg/m^2^ (normal weight)	4764 (40.6%)	595 (43.6%)	5359 (40.9%)	
25–29.9 kg/m^2^ (overweight)	4056 (34.6%)	387 (28.3%)	4443 (33.9%)	
≥ 30.0 kg/m^2^ (obese)	2155 (18.4%)	179 (13.1%)	2334 (17.8%)	
Missing	339 (2.9%)	117 (8.6%)	456 (3.5%)	
**Cancer site**				
Colon	7807 (66.6%)	1233 (90.3%)	9040 (69.0%)	< 0.001
Rectum	3869 (33.0%)	127 (9.3%)	3996 (30.5%)	
Missing	50 (0.4%)	6 (0.4%)	56 (0.4%)	
**AJCC preoperative T category**				
T1	2372 (20.2%)	204 (14.9%)	2576 (19.7%)	< 0.001
T2	2585 (22.0%)	145 (10.6%)	2730 (20.9%)	
T3	5056 (43.1%)	595 (43.6%)	5651 (43.2%)	
T4	1595 (13.6%)	400 (29.3%)	1995 (15.2%)	
Missing	118 (1.0%)	22 (1.6%)	140 (1.1%)	
**AJCC preoperative N category**				
N0	6639 (56.6%)	722 (52.9%)	7361 (56.2%)	0.008
N1	3350 (28.6%)	423 (31.0%)	3773 (28.8%)	
N2 or N3	1317 (11.2%)	181 (13.3%)	1498 (11.4%)	
Missing	420 (3.6%)	40 (2.9%)	460 (3.5%)	
**AJCC preoperative overall stage**				
I	3620 (30.9%)	193 (14.1%)	3813 (29.1%)	< 0.001
II	2592 (22.1%)	377 (27.6%)	2969 (22.7%)	
III	4173 (35.6%)	492 (36.0%)	4665 (35.6%)	
IV	811 (6.9%)	178 (13.0%)	989 (7.6%)	
Missing	530 (4.5%)	126 (9.2%)	656 (5.0%)	
**Surgical approach**				
Open	4847 (41.3%)	1124 (82.3%)	5971 (45.6%)	< 0.001
Minimally invasive	6851 (58.4%)	239 (17.5%)	7090 (54.2%)	
Missing	28 (0.2%)	3 (0.2%)	31 (0.2%)	

Values are *n* (%). ASA, American Society of Anesthesiology; AJCC, American Joint Commission on Cancer.

### Primary analysis: urgency of surgery and quality of cancer surgery

**Fig. 2:**
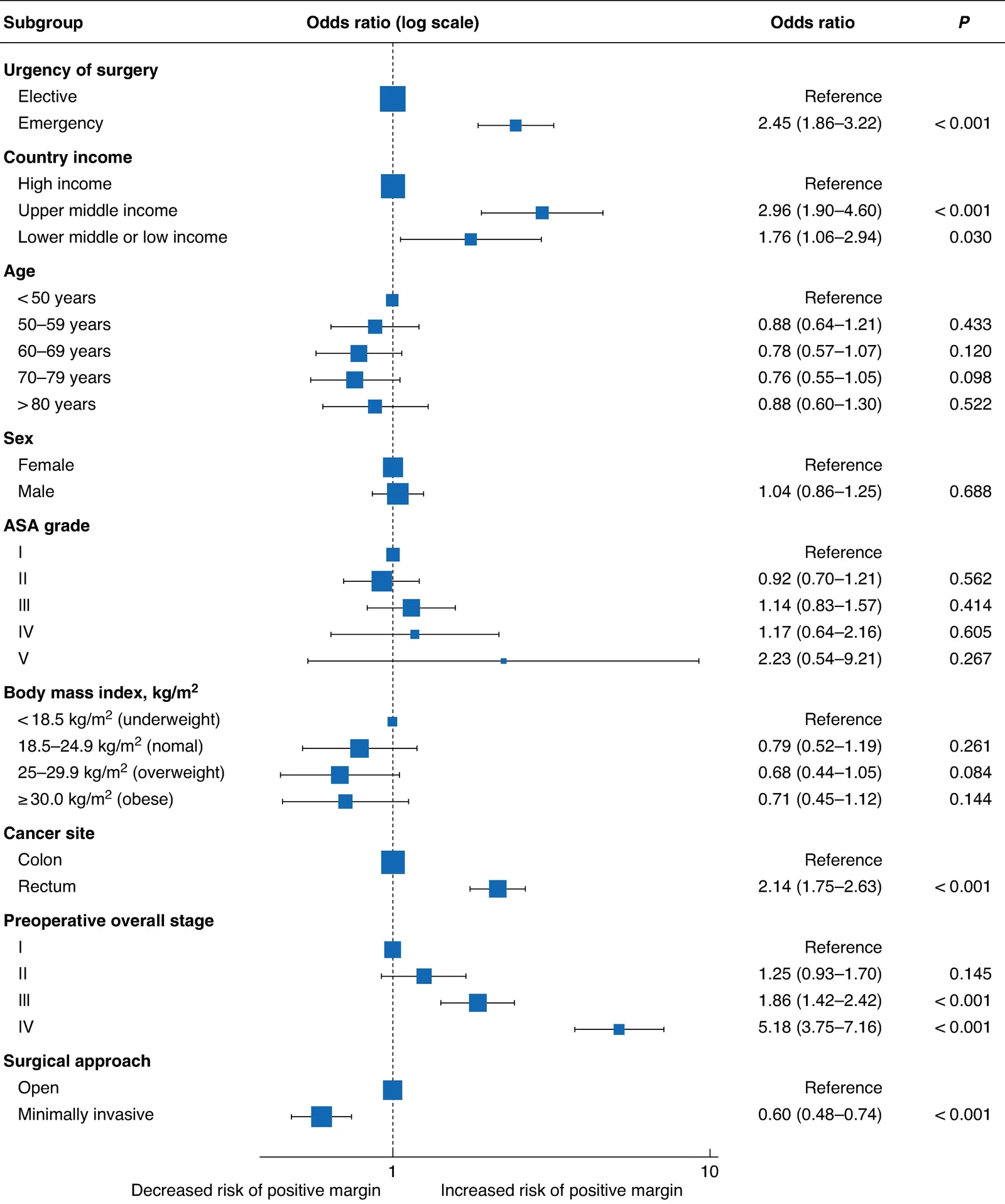
Multilevel logistic regression of patient, disease, and health system characteristics on margin-positive resection in patients undergoing potentially curative cancer surgery The multilevel model includes hospital and country identifier as random effects. Error bars and values in parentheses represent 95% confidence intervals. ASA, American Society of Anesthesiologists.

In the entire cohort, 678 patients (5.4%) had a margin-positive resection, with the rate being higher in patients undergoing emergency than elective cancer surgery (13.7 *versus* 4.5%; *P* < 0.0001). Margin-positive resection was more common in younger patients (8.9, 6.5, 4.5, 4.5, and 4.5% for age < 50, 50–59, 60–69, 70–79, and ≥ 80 years, respectively; *P* < 0.0001), those with ASA grade I (7.2 *versus* 4.7, 5.2, and 6.1% in grades II, III, and IV, respectively; *P* < 0.0001), and those with underweight or normal weight (9.8 and 5.7 *versus* 4.5 and 4.9% for overweight or obesity, respectively; *P* < 0.0001). Margin-positive resection was more common in patients with rectal than colon cancer (6.8 *versus* 4.8%; *P* < 0.0001), in those with preoperative stage III or IV *versus* stage I or II (6.1, 14.8, 2.8, and 4.7%, respectively), and in those undergoing open *versus* minimally invasive cancer surgery (7.7 *versus* 3.5%; *P* < 0.0001). A comparison of patient- and operative-level characteristics between patients with and without margin-positive resection is presented in *Tables S4* and *S5*. In an adjusted model accounting for patient and disease characteristics with hospital and country as random effects, patients undergoing emergency surgery were more likely to have a margin-positive resection (OR 2.45; 95% confidence interval (c.i.) 1.86 to 3.22; *P* < 0.0001). Surgery in upper-middle income (OR 2.96; 95% c.i. 1.90 to 4.60; *P* < 0.0001) and lower middle- or low-income countries (OR 1.76; 95% c.i. 1.06 to 2.94; *P* = 0.03) was associated with a higher rate of margin-positive resections than surgery in high income countries. Other factors associated with margin-positive resections were rectal cancers (OR 2.14; 95% c.i. 1.75 to 2.63; *P* < 0.0001) and stage III (OR 1.86; 95% c.i. 1.42 to 2.42; *P* < 0.0001) and stage IV (OR 5.18; 95% c.i. 3.75 to 7.16; *P* < 0.0001) cancers (*Fig. 2*). The full model is presented in *Table S6*.

**Fig. 3:**
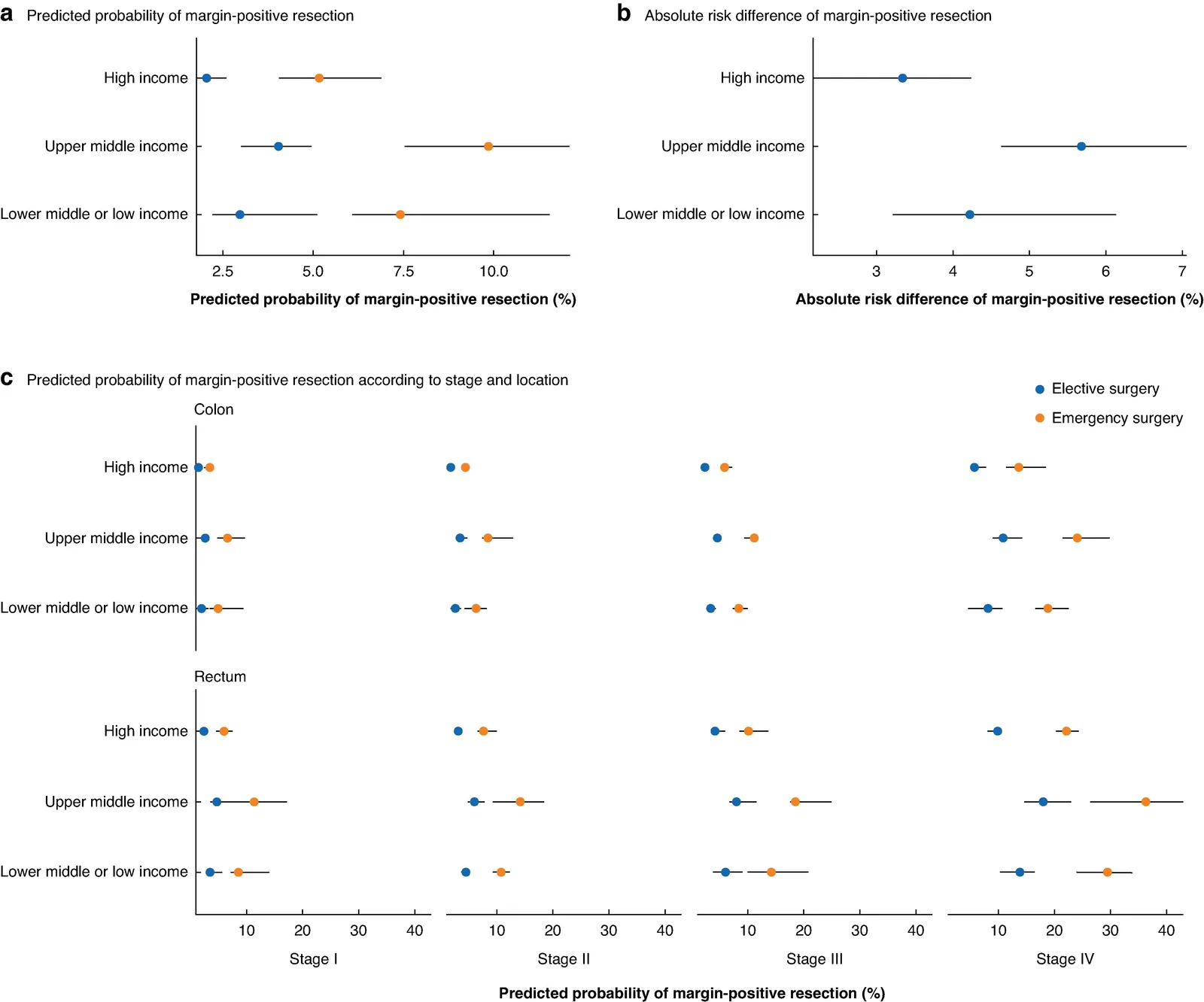
Association between the urgency of surgery and country income level from bootstrap simulations **a** Predicted probability and **b** absolute risk difference for margin-positive resection by urgency of surgery and country income level. **c** Predicted probability for margin-positive resection by urgency of surgery stratified by preoperative overall stage and cancer location (that is, colon, rectum). Data show odds ratio with 95% confidence interval.

Subgroup analysis were then conducted evaluating the preplanned interaction between urgency and country income level, cancer stage, and cancer site. Absolute risk differences revealed the greatest disparities in patients with stage III–IV rectal cancers, with absolute differences of 12.6% (95% c.i. 10.2 to 15.7; absolute risk for elective and emergency 9.9 *versus* 22.1%) in high-income countries, 19.0% (95% c.i. 13.6 to 23.9; absolute risk for elective and emergency 18.0 *versus* 36.3%) in upper-middle-income countries, and 14.1% (95% c.i. 10.9 to 16.5; absolute risk for elective and emergency 13.8 *versus* 29.4%) in lower middle- and low-income countries (*Fig. 3*) (*Tables S7* and *S8*). Despite this, the relative risk of margin-positive resection in emergency cancer surgery was consistent across country income groups, cancer stage, and site. Sensitivity analyses restricted to prepandemic cohorts (that is, GlobalSurg-3) demonstrated that emergency surgery remained strongly associated with margin-positive resection with similar effect estimates. There was no evidence that the observed association was confined to pandemic-era access constraints (*Table S11*).

**Fig. 4:**
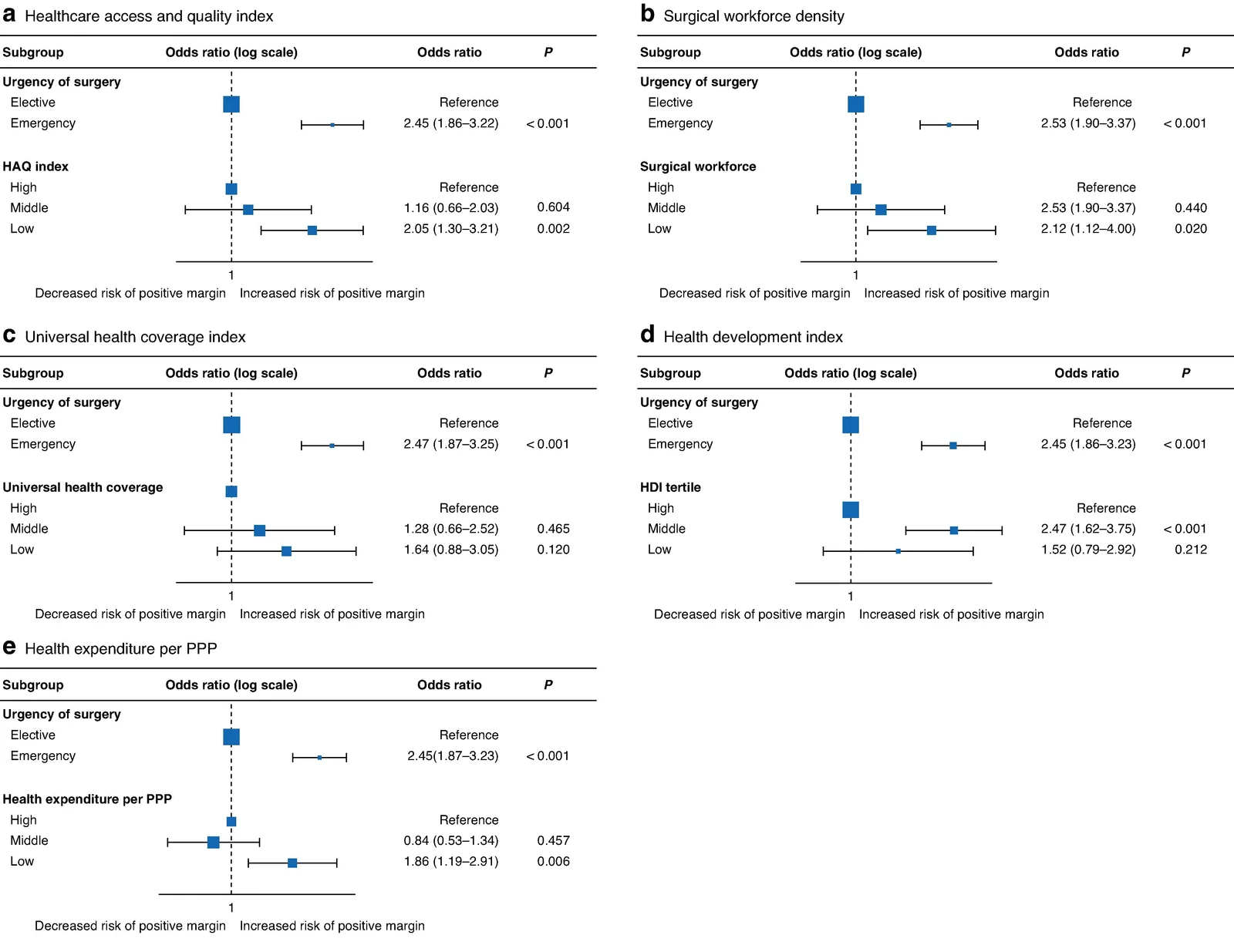
Multilevel logistic regression model in patients with potentially curative colorectal cancer evaluating associations between the urgency of surgery and margin status across different health system policy indices **a** Healthcare access and quality index, **b** surgical workforce density, **c** universal health coverage index, **d** human development index, and **e** healthcare expenditure per PPP. Error bars and values in parentheses represent 95% confidence intervals. PPP, power purchase parity.

Finally, an analysis was conducted to examine variation in outcomes across the models. In a four-level model of patients nested in hospitals, countries, and World Bank income groups, 76% of the variation in outcome captured by the model was explained by hospital, country, and country income group factors (*Table S9* and *Fig. S1*). This was reflected in moderate-to-substantial clustering. The intraclass correlation coefficient was 0.18 at the hospital level and 0.11 at the country level, indicating that outcomes for patients treated within the same hospital or country were meaningfully correlated. When primary models were developed replacing World Bank country income with other preplanned health system macro-level indicators, patients undergoing emergency surgery were more likely to have a margin-positive resection, independent of healthcare access and quality (*Fig. 4*) (*Tables S12–S14* and *Fig. S3*), universal health coverage (*Tables S15–S18*), specialist surgical workforce density (*Tables S19–S22*), human development index (*Tables S23–S26*), and healthcare expenditure (*Tables S27–S30*).

## Discussion

This preplanned secondary analysis of over 13 000 patients undergoing potentially curative colorectal cancer surgery across 95 countries identified a significant surgical quality gap in emergency settings, reflected by a two- to threefold higher risk of margin-positive resection. A key strength of this study was the ability to distinguish patients with curable disease from those with non-curative cancers (that is, obviously unresectable or perforated tumours). This disparity persisted after adjustment for patient, tumour, and country-level factors, and is consistent with contributions from modifiable system-level drivers such as clinical decision-making, surgeon availability, and operative technique. These findings highlight emergency surgical care as a neglected aspect of global oncology, for which immediate, targeted efforts will yield substantial improvements in outcomes.

Globally, surgical teams classify a potentially curative patient as an emergency when an acute oncological crisis, most commonly complete or impending large-bowel obstruction, imminent perforation, or life-threatening haemorrhage, demands resection within hours rather than weeks. In health systems that can deploy decompression stomas, endoscopic stents, or short-course neoadjuvant therapy, these crises are sometimes bridged into a planned elective pathway; however, such resources are patchily available, especially outside high-income settings. Consequently, the 48-h timing definition is more than a clock-based threshold; it is a real-world proxy for surgery performed without the usual safeguards of full staging, bowel preparation, multidisciplinary deliberation, and subspecialist availability. The baseline contrasts observed in this study, namely higher stage, predominance of colon cancers, and greater reliance on open surgery, highlight how this constrained decision space shapes both case mix and technical outcome.

These findings may have direct implications for frontline clinical practice globally. First, the decision to operate in emergency presentations requires careful consideration. In rectal cancer, where margin-positive resections are more common, curative resection should generally be deferred in favour of temporizing strategies such as defunctioning stomas and, in well-resourced centres, short-course neoadjuvant radiotherapy or chemoradiotherapy to downstage disease before surgery. In contrast, endoscopic stenting is best reserved for select left-sided colonic obstructions and is rarely appropriate for low rectal tumours^31^. The limited availability of such technology in Africa and Southeast Asia represents a missed, cost-effective opportunity to improve oncological outcomes. For colonic tumours, surgery should only proceed when a clear resection margin is realistically achievable based on imaging and clinical assessment. Second, surgical expertise appears critical. Complex emergency resections should be performed by colorectal specialists wherever possible. If unavailable, early referral to regional centres or adoption of non-resection strategies should be prioritized. These pragmatic, evidence-informed strategies provide immediate avenues to enhance emergency cancer care globally in an era when expanding access is severely constrained by funding limitations.

Although surgical oncology is increasingly recognized as fundamental to global cancer control efforts, systematic international assessments of the quality of cancer surgery remain limited, particularly regarding oncological outcomes beyond perioperative mortality. The Lancet Oncology Commission on Global Cancer Surgery^5^ highlighted the essential role that timely, safe, and effective surgical care plays in optimizing cancer survival outcomes; yet, existing research predominantly addresses perioperative mortality or severe postoperative complications^12^, often neglecting critical oncological quality measures such as resection margin status, lymph node yield, or adherence to multidisciplinary guidelines^7,8^. A recent systematic review including 89 studies identified substantial variability in quality measurement globally; notably, of the few studies examining margin positivity^32–34^, none evaluated its relationship with urgency of surgery, and all were based exclusively in high-income countries (USA and Canada). Furthermore, current knowledge predominantly attributes poorer emergency surgery outcomes to advanced disease stage at presentation, overlooking potential deficits in emergency surgical systems. By systematically demonstrating a global quality gap specifically in emergency cancer surgery, independent of disease stage, this study advances understanding and identifies actionable targets to close global surgical oncology disparities.

Several plausible pathways explain the consistently higher margin-positive resection rates observed in emergency colorectal cancer surgery. Emergency presentations limit the opportunities for comprehensive preoperative evaluation and informed clinical decision-making. Essential steps, such as computed tomography imaging and multidisciplinary team consultations, that are routinely undertaken in elective settings may be unavailable in emergency settings. In addition, the composition of surgical teams in emergency contexts often differs substantially from that of teams in elective care. Although elective colorectal surgeries are typically performed by specialized colorectal surgeons supported by dedicated teams experienced in complex cancer care, emergency colorectal procedures are performed by general surgeons, who may or may not have formal colorectal training, especially in low-resource settings and in some high-income settings with limited specialist availability.

The principal strength of this study lies in its large, globally representative cohort, comprising over 13 000 patients from 964 hospitals across 95 countries, and in a robust, prospectively collected data set. The use of standardized, validated methodologies^12,35^ provides insight into variations in surgical quality both within and between countries, identifying urgent and actionable targets for improving global cancer care. The analytical approach, incorporating multilevel clustering and sequential adjustment for macro-level health system indicators, strengthens confidence in the finding that surgical urgency independently influences oncological quality. However, several limitations should be acknowledged. First, the observational design limits causal inference. Despite comprehensive adjustment, residual confounding may persist, particularly from unmeasured factors such as tumour biology, surgeon experience, and intraoperative decision-making. Further, it was not possible to adjust for important pathway factors such as access to pelvic magnetic resonance imaging staging, neoadjuvant chemoradiotherapy, or watch-and-wait protocols, which are known to influence circumferential margin status independently of operative technique. These elements vary by resource setting and are likely to differ systematically between elective and emergency pathways. Second, resection margins were assessed locally without central pathology review. Although margin status was reported according to international standards (The Royal College of Pathologists and European Society for Medical Oncology guidelines) and assessed by consultant gastrointestinal pathologists, interobserver variation remains likely, particularly in settings without subspecialist colorectal pathology. Central slide review or formal international pathology calibration exercises were not undertaken for this secondary analysis. As a result, some non-differential measurement error in margin status is probable; this would tend to attenuate rather than exaggerate the association between emergency surgery and margin positivity. Third, although the inclusion of other key oncological outcomes, such as lymph node yield or longer-term measures (for example, recurrence, 90- or 365-day mortality), would provide deeper insight, these data were incomplete or inconsistent across data sets and would introduce heterogeneity due to variable follow-up protocols and health system capacity. In contrast, margin-positive status reflects surgical technique and decision-making at the point of care and is minimally dependent on postoperative care systems. Its accepted prognostic value is supported by multiple studies demonstrating strong associations with recurrence-free survival, local recurrence, and cancer-specific mortality, even after adjusting for tumour stage and patient factors. Fourth, reliance on national-level macro indicators may obscure subnational disparities, thereby limiting the precision with which outcomes can be attributed to resource constraints. Participation bias is also possible, because contributing centres may be better resourced, potentially underestimating the true global burden. Fifth, the inclusion period spanned the COVID-19 pandemic. Although the year of surgery and cohort were included in the models, pandemic-era theatre capacity constraints and altered triage pathways may have shifted some cases of clinical emergency into later, apparently elective time windows or, conversely, accelerated surgery in borderline elective patients. These temporal pressures may have introduced non-differential misclassification of urgency that could not be fully quantified. Sixth, urgency was defined by a fixed 48-h threshold, which, although aligned with international audit standards, may not perfectly discriminate clinical urgency across all settings; for example, pandemic-era theatre constraints or local triage protocols may delay an operation that was a clinical emergency. Conversely, access to bridging strategies could reschedule an otherwise emergency presentation into an elective pathway. These nuances could not be captured in the current data sets and represent an inherent limitation of large, pragmatic, global studies. Finally, the absence of data on key pathway components, such as preoperative imaging, stenting availability, and multidisciplinary involvement, limits mechanistic interpretation, and surgeon-level factors were not available. Despite these limitations, the consistent excess risk of margin-positive resection during emergency surgery across all income settings highlights an urgent need to strengthen global surgical systems.

## Collaborators

### Writing group

Sivesh K Kamarajah, Adewale O. Adisa, Ajay Aggarwal, Rifat Atun, Alazar Berhe, James Glasbey, Ewen Harrison, Parvez D Haque, Ismail Lawani, Sonia Mathai, Salome Maswime, Ana Minaya Bravo, CS Pramesh, April Camilla Roslani, Nobhojit Roy, Richard Sullivan, Sudha Sundar, Chris Varghese, Aneel A Bhangu

### Statistical analysis and data handling

Sivesh K Kamarajah, Aneel A Bhangu

### CovidSurg Cancer

#### *International Cancer Leads* (*denotes specialty Principal Investigators)

James C Glasbey (Chair); Colorectal: Neil J Smart*, Ana Minaya-Bravo*, Jonathan P Evans, Gaetano Gallo, Susan Moug, Francesco Pata, Peter Pockney, Salomone Di Saverio, Abigail Vallance, Dale Vimalchandran; Oesophagogastric: Ewen A Griffiths*, Sivesh K Kamarajah, Richard PT Evans, Philip Townend; Hepatopancreatobilary: Keith Roberts*, Siobhan McKay*, John Isaac, Sohei Satoi; Thoracic: John Edwards*, Aman S Coonar, Adrian Marchbank, Edward J Caruana, Georgia R Layton, Akshay Patel, Alessandro Brunelli; Sarcoma: Samuel Ford*, Anant Desai*, Alessandro Gronchi*, Marco Fiore*, Max Almond, Fabio Tirotta, Sinziana Dumitra; Neurosurgery: Angelos Kolias*, Stephen J Price, Daniel M Fountain, Michael D Jenkinson, Peter Hutchinson, Hani J Marcus, Rory J Piper, Laura Lippa, Franco Servadei, Ignatius Esene, Christian Freyschlag, Iuri Neville, Gail Rosseau, Karl Schaller, Andreas K Demetriades, Faith Robertson, Alex Alamri; Head and neck: Richard Shaw*, Andrew G Schache, Stuart C Winter, Michael Ho, Paul Nankivell, Juan Rey Biel, Martin Batstone, Ian Ganly; Breast: Raghavan Vidya*, Alex Wilkins, Jagdeep K Singh, Dinesh Thekinkattil; Gynaecology: Sudha Sundar*, Christina Fotopoulou*, Elaine YL Leung, Tabassum Khan, Luis Chiva, Jalid Sehouli, Anna Fagotti, Paul Cohen, Murat Gutelkin, Rahel Ghebre, Thomas Konney, Rene Pareja, Rob Bristow, Sean Dowdy, Shylasree TS, Rajkumar Kottayasamy Seenivasagam, Joe Ng, Keiichi Fujiwara; Urology: Grant D Stewart*, Benjamin Lamb, Krishna Narahari, Alan McNeill, Alexandra Colquhoun, John S McGrath, Steve Bromage, Ravi Barod, Veeru Kasivisvanathan*, Tobias Klatte.

### Dissemination Committee

Joana FF Simoes (Chair); Tom EF Abbott, Sadi Abukhalaf, Michel Adamina, Adesoji O Ademuyiwa, Arnav Agarwal, Murat Akkulak, Ehab Alameer, Derek Alderson, Felix Alakaloko, Markus Albertsmeier, Osaid Alser, Muhammad Alshaar, Sattar Alshryda, Alexis P Arnaud, Knut Magne Augestad, Faris Ayasra, José Azevedo, Brittany K Bankhead-Kendall, Emma Barlow, David Beard, Ruth A Benson, Ruth Blanco-Colino, Amanpreet Brar, Ana Minaya-Bravo, Kerry A Breen, Chris Bretherton, Igor Lima Buarque, Joshua Burke, Edward J Caruana, Mohammad Chaar, Sohini Chakrabortee, Peter Christensen, Daniel Cox, Moises Cukier, Miguel F Cunha, Giana H Davidson, Anant Desai, Salomone Di Saverio, Thomas M Drake, John G Edwards, Muhammed Elhadi, Sameh Emile, Shebani Farik, Marco Fiore, J Edward Fitzgerald, Samuel Ford, Tatiana Garmanova, Gaetano Gallo, Dhruva Ghosh, Gustavo Mendonça Ataíde Gomes, Gustavo Grecinos, Ewen A Griffiths, Magdalena Gruendl, Constantine Halkias, Ewen M Harrison, Intisar Hisham, Peter J Hutchinson, Shelley Hwang, Arda Isik, Michael D Jenkinson, Pascal Jonker, Haytham MA Kaafarani, Debby Keller, Angelos Kolias, Schelto Kruijff, Ismail Lawani, Hans Lederhuber, Sezai Leventoglu, Andrey Litvin, Andrew Loehrer, Markus W Löffler, Maria Aguilera Lorena, Maria Marta Modolo, Piotr Major, Janet Martin, Hassan N Mashbari, Dennis Mazingi, Symeon Metallidis, Ana Minaya-Bravo, Helen M Mohan, Rachel Moore, David Moszkowicz, Susan Moug, Joshua S Ng-Kamstra, Mayaba Maimbo, Ionut Negoi, Milagros Niquen, Faustin Ntirenganya, Maricarmen Olivos, Kacimi Oussama, Oumaima Outani, Marie Dione Parreno-Sacdalanm, Francesco Pata, Carlos Jose Perez Rivera, Thomas D Pinkney, Willemijn van der Plas, Peter Pockney, Ahmad Qureshi, Dejan Radenkovic, Antonio Ramos-De la Medina, Toby Richards, Keith Roberts, April C Roslani, Martin Rutegård, Juan José Segura-Sampedro, Irène Santos, Sohei Satoi, Raza Sayyed, Andrew Schache, Andreas A Schnitzbauer, Justina O. Seyi-Olajide, Neil Sharma, Catherine A Shaw, Richard Shaw, Sebastian Shu, Kjetil Soreide, Antonino Spinelli, Grant D Stewart, Malin Sund, Sudha Sundar, Stephen Tabiri, Philip Townend, Georgios Tsoulfas, Gabrielle H van Ramshorst, Raghavan Vidya, Dale Vimalachandran, Oliver J Warren, Duane Wedderburn, Naomi Wright, EuroSurg, European Society of Coloproctology (ESCP), Global Initiative for Children’s Surgery (GICS), GlobalSurg, GlobalPaedSurg, ItSURG, PTSurg, SpainSurg, Italian Society of Colorectal Surgery (SICCR), Association of Surgeons in Training (ASiT), Irish Surgical Research Collaborative (ISRC), Transatlantic Australasian Retroperitoneal Sarcoma Working Group (TARPSWG), Italian Society of Surgical Oncology (SICO).

*Patient advisory group:* Lesley Booth (UK, patient involvement lead), Margaret Barker (UK), Neil Barker, Shirley Cooke (UK), Suzanne Doré (UK), Nigel Horwood (UK), Emmy Runigamugabo (Rwanda), Carrie Tierney Weir (UK).

#### *Collaborators* (*denotes hospital lead(s))

Albania: Dajti I (University Hospital Center Nene Tereza, Tirana).

Argentina: Allemand C, Boccalatte LA*, Figari M, Lamm M, Larrañaga J, Marchitelli C, Noll F*, Odetto D, Perrotta M, Saadi J, Zamora L (Hospital Italiano De Buenos Aires, Buenos Aires); Ballester AM, Tapper KE, Zeff N* (Hospital Universitario Cemic, Buenos Aires); Valenzuela JI* (Hospital Velez Sarsfield, City Of Buenos Aires); Alurralde C, Anastasio J, Apas Perez de Nucci A, Caram EL, Eskinazi D*, Mendoza JP, Usandivaras M (Sanatorio 9 De Julio Sa, Tucuman); Badra R, Esteban A, García JS, García PM, Gerchunoff JI, Lucchini SM*, NIgra MA, Vargas L (Sanatorio Allende, Cordoba).

Armenia: Hovhannisyan T*, Stepanyan A* (Nairi Medical Center, Yerevan).

Australia: Vasey CE*, Watson EGR (Ballarat Base Hospital, Ballarat); Ip C*, Kealey J, Lim CSH, Sengupta S*, Ward S*, Wong E* (Box Hill Hospital, Melbourne); Gould T, Gourlay R*, Griffiths B (Calvary Mater Newcastle, Newcastle); Gananadha S*, McLaren M (Canberra Hospital, Canberra); Cecire J, Joshi N, Salindera S*, Sutherland A (Coffs Harbour Health Campus, Coffs Harbour Nsw); Ahn JH, Charlton G, Chen S, Gauri N, Hayhurst R, Jang S, Jia F, Mulligan C, Yang W, Ye G, Zhang H (Concord Repatriation General Hospital, Concord West, Sydney); Ballal M, Gibson D, Hayne D, McMillan H, Moss J*, Pugliese MJ, Richards T, Seow YTN, Thian A, Viswambaram P, Vo UG* (Fiona Stanley Hospital, Perth); Bennetts J*, Bright T*, Brooke-Smith M*, Fong R, Gricks B, Huang L, Lam YH, Nathan A, Ong BS, Ooi E, Szpytma M, Watson D (Flinders Medical Centre, Adelaide); Bagraith K, Caird S, Chan E, Dawson C, Ho D, Hui N, Izwan S, Jeyarajan E, Jordan S, Liang R, Lim A, Nolan GJ, Oar A, Parker D, Puhalla H, Quennell A, Rutherford L, Sommerville C, Townend P*, Von Papen M, Wullschleger M (Gold Coast University Hospital, Southport); Dawson AC*, Drane A (Gosford Hospital, Gosford); Blatt A*, Cope D, Egoroff N, Fenton M, Gani J, Lott N, Pockney P*, Shugg N (John Hunter Hospital, Newcastle); Elliott M, Phung D (Lifehouse, Sydney); Phan D, Townend D* (Lismore Base Hospital, Lismore); Bong C, Gundara J* (Logan Hospital, Brisbane); Frankel A* (Princess Alexandra Hospital, Brisbane); Bowman S*, Guerra GR (Queen Elizabeth II Jubilee Hospital, Brisbane); Gerns N, McGeorge S, Riddell A*, Roberts M*, Rukin N (Redcliffe Hospital, Redcliffe); Bolt J, Buddingh K, Dudi-Venkata NN, Jog S, Kroon HM*, Sammour T, Smith R, Stranz C (Royal Adelaide Hospital, Adelaide); Batstone M*, Lah K*, McGahan W*, Mitchell D*, Morton A, Pearce A, Roberts M*, Sheahan G*, Swinson B (Royal Brisbane And Women’s Hospital, Brisbane); Waldron A, Walker P* (St John Of God Midland Public And Private Hospital, Perth); Alam N, Banting S, Chong L, Choong P*, Clatworthy S, Foley D, Fox A, Hii MW, Knowles B, Mack J, Read M, Rowcroft A, Ward S*, Wright G* (St Vincent’s Hospital, Melbourne); Dawson AC*, Drane A*, Lun EWY* (Wyong Public Hospital, Wyong).

Austria: Lanner M* (Kardinal Schwarzenberg Klinikum, Schwarzach Im Pongau); Burtscher J, Trivik- Barrientos F* (Landesklinikum Wiener Neustadt, Wiener Neustadt); Königsrainer I* (Landeskrankenhaus Feldkirch, Feldkirch); Bauer M, Freyschlag C, Kafka M, Messner F, Öfner D*, Tsibulak I (Medical University Of Innsbruck, Innsbruck); Holawe S, Zimmermann M* (Medical University Of Vienna, Vienna); Emmanuel K, Grechenig M, Gruber R, Harald M, Öhlberger L, Presl J*, Wimmer A (Paracelsus Medical University Salzburg, Salzburg).

Azerbaijan: Namazov İ, Samadov E (Leyla Medical Centerl, Baku).

Barbados: Barker D, Boyce R, Corbin S, Doyle A, Eastmond A, Gill R, Haynes A, Millar S, O’Shea M, Padmore G*, Paquette N, Phillips E, St. John S, Walkes K (Queen Elizabeth Hospital, Bridgetown).

Belgium: Abeloos J, De Backer T, De Ceulaer J*, Dick C*, Diez-Fraile A, Lamoral P, Spaas C (Az Sint-Jan Brugge-Oostende Av, Brugge); Ceelen W, Pattyn P, Van de putte D, Van Nieuwenhove Y, Van Ramshorst G*, Willaert W (Ghent University Hospital, Ghent);.

Botswana: Bazzett-Matabele L*, Chiyapo SP, Ramogola-Masire D*, Ramontshonyana G, Seiphetlheng A, Vuylsteke P (Princess Marina Hospital, Gaborone).

Brazil: Abdallah EA, Aguiar Júnior S*, Baiocchi G*, Carvalho GB, Coimbra FJF*, Kowalski LP*, Makdissi F, Marques N, Marques T, Soares Dos Santos S, Tirapelli Gonçalves B, Vartanian JG (A.C. Camargo Cancer Center, São Paulo); Dos Reis R* (Barretos Cancer Hospital, Barretos); Camara P*, De Lima RK, Della Giustina E, Hoffmann PV (Fundação Hospitalar De Blumenau, Blumenau); Gatti A*, Nardi C, Oliva R (Hospital Geral De Pirajussara, Taboão Da Serra); Nacif L* (Hospital Nove De Julho, Sao Paulo); Carvalho Ferro C, Gomes Mendonça Ataíde G, Lima Buarque I, Lira dos Santos Leite A, Pol-Fachin L, Santos Bezerra T, Maylson Ramos da Silva A, Windson de Araújo Silvestre D, Vieira Barros A* (Hospital Santa Casa De Misericordia De Maceio, Maceio); Campbell L* (Hospital Santa Helena, Brasília); De Cicco R* (Instituto De Câncer Dr Arnaldo Vieira De Carvalho, São Paulo); Cecconello I, Gregorio P, Pontual Lima L, Ribeiro Junior U, Takeda FR*, Terra RM* (Instituto Do Câncer De Estado De São Paulo, São Paulo); Faccini Teixeira M, Kowalski LP, Kulcsar MAV, Matos LL*, Nunes KS (Instituto Do Cancer Do Estado De São Paulo, Sao Paulo); Laporte G*, Salem M (Irmandade Da Santa Casa De Misericórdia De Porto Alegre, Porto Alegre); Barakat Awada J, Ijichi TR, Kim NJ, Marreiro A, Muller B, Nunes R* (Notre Dame Intermédica - Hospital Salvalus, São Paulo); Bodanese B, Eidt ER, Isoton JC, Lemos Vieira da Cunha M*, Regina de Sampaio L, Vendrame C*, Zeni M*, Zortéa JA, Zortéa MR* (Supera Oncologia - Hospital Regional Do Oeste, Chapeco).

Bulgaria: Sokolov M* (University Hospital Alexandrovska, Sofia).

Canada: Kidane B*, Srinathan S (Health Sciences Centre, Winnipeg); Munro A*, Helyer L, McKeen D (IWK Health Centre, Halifax); Boutros M*, Caminsky NG, Ghitulescu G, Jamjoum G, Moon J, Pelletier J, Vanounou T, Wong S (Jewish General Hospital, Montreal); Cheng D, MacNeil SD, Martin J* (London Health Sciences Centre And St Josephs Health Care London, London); Boutros M, Dumitra S*, Kouyoumdjian A, Schmid S, Spicer J (Mcgill University Health Center, Montreal); Agarwal A, Brar A, Dada J, Dare A, Hameed U*, Osman F (North York General Hospital, Toronto); Johnston B*, Russell C (Saint John Regional Hospital, Saint John); Groot G*, Persad A, Pham H, Wood M (Saskatoon City Hospital/Royal University Hospital/St. Paul’s Hospital, Saskatoon Sk); Brar A, Ko M*, Rajendran L (St. Joseph’s Health Centre, Toronto); Boutros M*, Demyttenaere S*, Garfinkle R (St. Mary’s Hospital, Montreal); Brown C*, Karimuddin A, Lee N, Liu J, Madani Kia T, Phang PT, Raval M, Tom K (St. Paul’s Hospital, Vancouver, Bc); Abou-Khalil J, Martel A, Nessim C*, Stevenson J (The Ottawa Hospital, Ottawa); Al Riyami S, Bali K, Bigam D*, Dajani K, Dell A (University Of Alberta Hospital, Edmonton).

Chile: Modolo MM*, Ramirez Nieto P, Sepulveda R, Molero A, Bolbaran A, Ruiz I (Barros Luco Trudeau, Santiago); Heredia F* (Clínica Universitaria De Concepción, Concepción); Bellolio F*, Besser N, Grasset E*, Guaman JO, Inzunza M, Irarrázaval MJ, Jarry C, Quintana Martinic M, Riquoir Altamirano C, Romero Manqui CA, Ruiz Esquide M, Vargas Añazco C (Hospital Clinico Universidad Católica, Santiago).

Colombia: Almeciga A*, Fletcher A*, Merchan A* (Centro De Investigaciones Oncológicas Clínica San Diego - Ciosad, Bogotá); Merchan A* (Clinaltec: Clinica Internacional De Alta Tecnologia En Cancer, Ibague); Quijano T, Sanabria D* (Clínica Los Nogales, Bogota); Arias-Amézquita F*, Cétares C, Cortes Murgueitio N, Gomez-Mayorga JL, Herrera-Almario G*, Rodriguez J*, Sanabria D (Fundacion Santa Fe De Bogota, Bogota); Iglesias P, Puentes LO* (Hospital San José, Bogotá); Calvache JA*, Orozco-Chamorro CM, Rojas DA, Sánchez-Gómez A (Hospital Universitario San José, Popayán); Abadia M, Acosta J, Almeciga A, Angel Aristizabal J, Bonilla A, Caicedo L, Calderon Quiroz PH, Cervera Bonilla S, Diaz S*, Facundo H, Garcia Mora M, Guevara O*, Guzman L, Herrera Mora DR, Jimenez Ramirez LJ, Lehmann C, Manrique E, Mariño I, Medina M, Pinilla Morales RE*, Puerto A, Puerto Horta J, Quintero M, Rey Ferro M, Rodriguez J, Saénz A, Santana D, Serrano W, Suescun O, Trujillo Sanchez LM*, Velasquez Cuasquen BG (Instituto Nacional De Cancerologia, Bogota); Mendoza Quevedo J* (Subred Sur Occidente De Kennedy (Hospital De Kennedy), Bogota).

Croatia: Bačić G, Karlović D, Kršul D, Zelić M* (University Hospital Center Rijeka, Rijeka); Luksic I*, Mamic M (University Hospital Dubrava, Zagreb); Bacic I, Bakmaz B, Ćoza I, Dijan E, Katusic Z, Mihanovic J*, Morović D, Rakvin I (Zadar General Hospital, Zadar).

Cyprus: Almezghwi H, Arslan K, Besim H, Özant A, Özçay N* (Near East University Hospital, Nicosia); Frantzeskou K, Gouvas N*, Kokkinos G, Papatheodorou P, Pozotou I, Stavrinidou O, Yiallourou A* (Nicosia General Hospital, Nicosia).

Czechia: Martinek L, Skrovina M*, Straka M, Szubota I (Hospital & Oncological Centre Novy Jicin, Novy Jicin); Peteja M, Žatecký J* (Slezská Nemocnice V Opavě, P.o., Opava); Javurkova V, Klat J* (University Hospital Ostrava, Ostrava).

Denmark: Antony S, Avlund T, Berg KD, Borre M, Christensen P*, Elkjær MC, Ernst A, Fensman SK, Haldrup M, Harbjerg JL, Iversen LH, Jensen PT*, Jeppesen TD, Kjaer DW, Kristensen HØ, Lund N, Maigaard Axelsen S, Mekhael M, Mikic N, Ostenfeld EB (Aarhus University Hospital, Aarhus); Ebbehøj AL, Krarup P, Schlesinger N, Smith H* (Bispebjerg Hospital, Copenhagen).

Dominican Republic: Batista S*, Crespo A, Díaz PJ, Rivas R, Rodriguez-Abreu J*, Tactuk N (Cedimat - Centro De Diagnóstico, Medicina Avanzada, Laboratorio Y Telemedicina, Santo Domingo).

Egypt: El Kassas M*, Omar W, Tawheed A (Helwan University, Cairo); Talaat M (Ain Shams University Specialized Hospital, Cairo); Abdelsamed A, Azzam AY*, Salem H*, Seleim A (Al Azhar University Hospitals, Cairo); Abdelmajeed A, Abdou M, Abosamak NE, AL Sayed M, Ashoush F*, Atta R, Elazzazy E, Elnemr M, Elsayed Hewalla ME, Elsherbini I, Essam E, Ewedah M, Ghallab I, Hassan E, Ibrahim M, Metwalli M, Mourad M, Qatora MS, Ragab M, Sabry A*, Saifeldin H, Samih A, Samir Abdelaal A, Shehata S*, Shenit K (Alexandria Main University Hospital, Alexandria); Attia D, Kamal N, Osman N* (Alexandria Medical Research Institute, Alexandria); Abbas AM*, Abd Elazeem HAS, Abd-Elkariem AY, Abdelkarem MM, Alaa S, Ashraf M, Ayman A, Azizeldine MG, Elkhayat H*, Emad Mashhour A, Gaber M, Hamza HM, Hawal I*, Hetta HF, K. Ali A, M.elghazaly S, Mohammed MM*, Monib FA, Nageh MA, Saad A, Saad MM*, Shahine M*, Yousof EA, Youssef A (Assiut University Hospital, Assiut); El-Deeb M, Fawzy M, Ghaly G, Ibraheem M* (Baheya Foundation For Treatment Of Breast Cancer, Giza); Eldaly A* (El-Menshawy Hospital, Tanta); Esmail E (Kafr Elzyat Hospital, Tanta); ElFiky M*, Nabil A (Kasr Alainy Faculty Of Medicine, Cairo University, Cairo); Alrahawy M*, Sakr A*, Soliman H*, Soltan H* (Menofiya University Hospital, Menoufia); Amira G, Sallam I*, Sherief M, Sherif A (Misr Cancer Center, Al Jizah); Abdelrahman A, Aboulkassem H, Ghaly G*, Hamdy R, Morsi A, Salem H*, Sherif G (National Cancer Institute, Cairo); Abdeldayem H, Abdelkader Salama I*, Balabel M, Fayed Y, Sherif AE* (National Liver Institute, Menoufia University, Shibin Elkom); Elmorsi R*, Emile S, Refky B* (Oncology Center Mansoura University, Mansoura); Abd-elsalam S, Badr H, Elbahnasawy M*, Elzoghby M, Essa M, Gamal Badr S, Ghoneim A*, Hamad O, Hamada M, Hammad M, Hawila A, Morsy MS, Salman S, Sarsik S (Tanta University Hospital, Tanta).

Ethiopia: Bekele K* (Maddawalabu University Goba Referral Hospital, Goba).

Finland: Kauppila JH*, Sarjanoja E (Länsi-Pohja Central Hospital, Kemi); Helminen O, Huhta H, Kauppila JH* (Oulu University Hospital, Oulu).

France: Beyrne C, Jouffret L*, Lugans L, Marie-Macron L (Centre Hospitalier Avignon, Avignon); Chouillard E*, De Simone B* (Centre Hospitalier Intercommunal Poissy Saint Germain En Laye, Poissy); Fredon F*, Roux A (Centre Hospitalier Roland Mazoin, Saint-Junien); Bettoni J, Dakpé S, Devauchelle B, Lavagen N, Testelin S* (Chu Amiens, Amiens); Boucher S*, Breheret R, Gueutier A, Kahn A, Kün-Darbois J (Chu Angers, Angers); Barrabe A, Lakkis Z*, Louvrier A, Manfredelli S, Mathieu P (Chu Besançon, Besancon); Chebaro A*, Drubay V, El amrani M, Eveno C, Lecolle K, Legault G, Martin L, Piessen G*, Pruvot FR, Truant S, Zerbib P (Chu Lille, Lille); Ballouhey Q*, Barrat B, Fourcade L, Laloze J, Salle H, Taibi A, Tricard J, Usseglio J (Chu Limoges, Limoges); Bergeat D, Merdrignac A (Chu Rennes - General Surgery, Rennes); Le Roy B, Perotto LO, Scalabre A* (Chu Saint Etienne, Saint Etienne); Gornes H, Vaysse C*, Vergriete K (Chu Toulouse, Toulouse); Aimé A, Ezanno A*, Malgras B (Hia Begin, St Mande); Arnaud AP*, Fustec E, Lavoue V, Tesson C (Hopital Anne De Bretagne Chu Rennes - General Surgery, Rennes); Bouche P*, Tzedakis S* (Hôpital Cochin - Aphp, Paris); Cotte E, Glehen O, Lifante J (Hopital Lyon Sud, Pierre Bénite); Bendjemar L, Braham H, Charre L, El Arbi N, Morel-chevillet L, Police A*, Villefranque V, Volpin E (Hôpital Simone Veil, Eaubonne); D’Urso A, Felli E, Mutter D, Pessaux P, Seeliger B* (Strasbourg University Hospitals, IHU-Strasbourg, Strasbourg); Barbé Y, Bardet J, Barret E, Berry R, Boddaert G, Bonnet S, Brian E, Cathala N, Cathelineau X, Denet C, Fuks D, Gossot D, Grigoroiu M, Laforest A, Levy-Zauberman Y, Louis-Sylvestre C, Macek P, Mombet A, Moumen A, Pourcher G, Rozet F, Sanchez Salas R, Seguin- givelet A*, Tribillon E (Institut Mutualiste Montsouris, Paris); Crenn V, De Vergie S, Duchalais E*, Espitalier F, Ferron C, Fragnaud H, Malard O*, Regenet N*, Rigaud J*, Varenne Y, Waast D* (Nantes University Hospital, Nantes).

Germany: Bork U*, Distler M, Fritzmann J, Kirchberg J, Praetorius C, Riediger C, Weitz J, Welsch T, Wimberger P* (University Hospital Carl Gustav Carus, TU Dresden, Dresden); Beyer K, Kamphues C*, Lauscher J, Loch FN, Schineis C (Charité University Medicine, Campus Benjamin Franklin, Berlin); Albertsmeier M*, Angele M, Kappenberger A, Niess H, Schiergens T, Werner J (Department Of General, Visceral And Transplantation Surgery, Ludwig-Maximilians-Universität Munich); Becker R*, Jonescheit J (Heilig-Geist Hospital Bensheim, Bensheim); Doerner J*, Seiberth R (Helios Universitätsklinikum Wuppertal – Universität Witten/Herdecke, Wuppertal); Pergolini I, Reim D* (Klinikum Rechts Der Isar TUM School Of Medicine, Munich); Herzberg J*, Honarpisheh H*, Strate T* (Krankenhaus Reinbek St. Adolf-Stift, Reinbek); Boeker C, Hakami I*, Mall J* (KRH Nordstadt-Siloah Hospitals, Hannover); Liokatis P*, Smolka W (LMU Klinikum Campus Innenstadt); Vassos N* (Mannheim University Medical Center (Universitätsmedizin Mannheim), Mannheim); Nowak K*, Reinhard T* (Romed Klinikum Rosenheim, Rosenheim); Hölzle F, Modabber A*, Winnand P (University Hospital Aachen, Aachen); Anthuber M, Shiban E, Sommer B, Sommer F, Wolf S* (University Hospital Augsburg, Augsburg); Howaldt H, Knitschke M* (University Hospital Giessen And Marburg, Giessen); Kauffmann P, Wolfer S* (University Hospital Goettingen / Universitätsmedizin Goettingen, Goettingen); Kleeff J, Lorenz K, Michalski C, Ronellenfitsch U*, Schneider R (University Hospital Halle (Saale), Saale); Bertolani E, Königsrainer A*, Löffler MW, Quante M*, Steidle C, Überrück L, Yurttas C (University Hospital Tübingen, Tübingen); Betz CS, Bewarder J, Böttcher A, Burg S, Busch C, Dreimann M, Frosch KH, Gosau M*, Heuer A, Izbicki J, Klatte TO, Koenig D, Moeckelmann N, Nitschke C, Perez D, Priemel M, Reiter A, Smeets R, Speth U, Stangenberg M, Thole S, Uzunoglu FG*, Viezens L, Vollkommer T, Zeller N (University Medical Center Hamburg-Eppendorf, Hamburg); Battista MJ*, Gillen K, Hasenburg A, Krajnak S, Linz VC, Schwab R (University Of Mainz, Department Of Gynaecology And Obstetrics, Mainz).

Ghana: Amo-Antwi K, Appiah-kubi A, Konney T*, Tawiah A (Komfo-Anokye Teaching Hospital, Kumasi); Boatey S, Issaka A, Korsah MA, Sheriff M* (Tamale Teaching Hospital, Tamale).

Greece: Angelou K, Haidopoulos D*, Rodolakis A (Alexandra General Hospital, Athens); Antonakis P, Bramis K, Chardalias L, Contis I, Dafnios N, Dellaportas D, Fragulidis G, Gklavas A, Konstadoulakis M, Memos N*, Papaconstantinou I*, Polydorou A, Theodosopoulos T, Vezakis A (Aretaieion Hospital, Athens); Antonopoulou MI, Manatakis DK*, Tasis N (Athens Naval And Veterans Hospital, Athens); Arkadopoulos N, Danias N, Economopoulou P, Frountzas M, Kokoropoulos P, Larentzakis A, Michalopoulos N*, Nastos C, Parasyris S, Pikoulis E, Selmani J, Sidiropoulos T, Vassiliu P (Attikon University General Hospital, Athens); Bouchagier K*, Klimopoulos S, Paspaliari D, Stylianidis G (Evaggelismos General Hospital, Athens); Akrivou D, Baxevanidou K, Bouliaris K, Chatzikomnitsa P, Delinasios G, Doudakmanis C, Efthimiou M, Giaglaras A, Kalfountzos C*, Kolla C, Koukoulis G, Zervas K, Zourntou S (General Hospital Of Larissa “Koutlimpaneio And Triantafylleio”, Larissa); Baloyiannis I, Diamantis A, Gkrinia E, Hajiioannou J*, Korais C, Koukoura O, Perivoliotis K, Saratziotis A, Skoulakis C, Symeonidis D, Tepetes K, Tzovaras G*, Zacharoulis D (General University Hospital Of Larissa, Larrisa); Alexoudi V, Antoniades K*, Astreidis I, Christidis P, Deligiannidis D, Grivas T, Ioannidis O*, Kalaitsidou I, Loutzidou L, Mantevas A, Michailidou D, Nikolaidou E, Papadopoulou S, Paraskevopoulos K, Politis S, Stavroglou A, Tatsis D, Tilaveridis I, Vahtsevanos K, Venetis G (George Papanikolaou General Hospital Of Thessaloniki, Thessaloniki); Karaitianos I*, Tsirlis T (Henry Dunant Hospital Center, Athens); Dinas K*, Margioula-Siarkou C, Petousis S (Hippocratio Hospital, Thessaloniki); Baili E, Charalabopoulos A, Liakakos T, Schizas D*, Spartalis E, Syllaios A, Zografos C (Laiko University Hospital, Athens); Anthoulakis C, Christou CD, Papadopoulos V, Tooulias A, Tsolakidis D*, Tsoulfas G*, Zouzoulas D (Papageorgiou General Hospital, Thessaloniki); Athanasakis E, Chrysos E, Tsiaoussis I, Xenaki S*, Xynos E* (University Hospital Of Heraklion Crete And Interclinic Hospital Of Crete, Heraklion Crete).

Guatemala: Barrios Duarte A*, Lopez Muralles I, Lowey MJ, Portilla AL, Recinos G (Hospital General De Enfermedades, Guatemala City).

Hong Kong: Chan JYK, Chan SM, Chong CCN, Futaba K*, Ho MF, Hon SF, Lau RWH, Mak TWC, Ng CF, Ng CSH, Ng KKC, Ng SSM, Teoh AYB, Teoh JY (Prince Of Wales Hospital, Sha Tin); Foo CC* (Queen Mary Hospital, Pok Fu Lam).

Hungary: Banky B*, Suszták N (Szent Borbála Kórház, Tatabánya).

India: Misra S*, Pareek P, Vishnoi JR* (All India Institute of Medical Sciences Jodhpur); Ambre S, Balasubiramaniyan V, Chappity P, Chaudhary I, Colney L, Das MK*, Imaduddin M, Jain A, Jena SK, Kar M*, Mandal S, Mishra A, Mishra SS, Mishra TS, Mitra JK, Mittal Y, Muduly DK*, Nayak P, Parida PK, Pradhan P, Rajan DK, Rebba E, Samal DK, Singh A, Sultania M* (All India Institute Of Medical Sciences, Bhubaneswar); Agarwal SP, Agrawal A, Arora RK*, Chaturvedi J, Garg PK, Gaurav A, Gupta A, Kottayasamy Seenivasagam R*, Maharaj DD, Majumdar KS, Mishra N, Mittal A*, Narain TA, Nirjhar R, Poonia DR*, Sadhasivam S, Singh MP, Tiwari AR* (All India Institute Of Medical Sciences, Rishikesh); Akula AK, Bandegudda SK, Bindlish RP, Chaitanya A, Chandrasekhara Rao LM, Dalakoti P, Dasu S, Giridhar A, Gorijavolu NB, Iyer RR, Jayakarthik Y, Jonathan GT, Kalla MB, Kheni Y, Kumar CS, Murtuza SA, Naidu CCK, Nalukurthi RK, Nemade HO, Nusrath S, Patnaik SC, Raju KVVN, Ramalingam PR, Rao KV, Rayani BK, Reddy Kallam SM, Reddy SRR, Saksena AR, Sebastian JA, Sharma RM, Thammineedi SR* (Basavatarakam Indo American Cancer Hospital & Research Institute, Hyderabad); Krishnamurthy A, Madhupriya S, Raja A, Ramakrishnan AS* (Cancer Institute (WIA) Chennai); Dutt UK, Ghosh DN, Grewal S, Hans P, Haque PD*, Jain R, Kingsley PA, Mahajan A, Mandrelle K*, Michael V, Mukherjee P, Varghese A, Varghese SS, Veetil SK (Christian Medical College & Hospital, Ludhiana); Gaikwad P, George AJ, James SM, Jesudason MR, Mittal R*, Moorthy M, Riju J, Sebastian A, Sen S, Singh S, Sreekar D, Thomas V, Titus DK, Yezzaji HS (Christian Medical College & Hospital, Vellore); Aggarwal M, Dhamija P, Kumar A* (Government Medical College Patiala, Patiala); Chisthi MM, Gejoe G, Gopakumar D, Kollengode VV*, Kuttanchettiyar KG, Yadev I* (Government Medical College Thiruvananthapuram, Thiruvananthapuram); Balasubramanian A, Chaturvedula L, Dharanipragada K*, Kalayarasan R*, Manikandan R, Penumadu P* (Jawaharlal Institute Of Postgraduate Medical Education And Research, Pondicherry); Lakshminarayana B, Mathew S* (Kasturba Medical College Hospital, Manipal, Manipal); Reddihalli PV*, Shivdas S* (Kidwai Memorial Institute Of Oncology, Bengaluru); Akhtar N, Chaturvedi A, Gupta S, Kumar V, Rajan S* (King George’s Medical University, Lucknow); Agrawal N, Ahluwalia P, Arora A, Batra-Modi K, Biswas M, Chaturvedi A, Chaturvedi H, Gautam G, Jain M, Jain S, Kumar S*, Nayyar R, Singh S, Tiwari A (Max Superspeciality Hospital, New Delhi); Bhushan Rangappa V, Kadapathri A, Kolur T, Pethkar R, Pillai V*, Popli G, Sharma J, Shetty V, Subramaniam N, Williams J (Mazumdar Shaw Cancer Centre, Narayana Health, Bengaluru); Agarwal P, Agarwal V, Baghel A, Sharma DB, Silodia A, Singh KN, Yadav SK* (Netaji Subhash Chandra Bose Medical College, Jabalpur); Aziz G, Chowdri N, Mehraj A*, Parray FQ, Shah ZA, Wani RA (Sher-I-Kashmir Institute Of Medical Sciences, Srinagar); Ahmed Z, Bali RS, Bhat MA, Laharwal AR, Mahmood M, Mir IS, Muzamil J, Najar FA, Rashid A*, Rather MH, Zaieem M (SMHS Hospital, Government Medical College, Srinagar); Aggarwal G*, Agrawal V, Ahmed A, Ahmed R, Bhaumik J, Ghosh A, Gupta S, Jain D, Jain PV, Kewlani V, Pipara A, Shakya S, Sharma A*, Thambudorai R (Tata Medical Center, Kolkata); Badwe RA, Bakshi G, Chandankhede U, Chaudhari V, Chaukar D, Chitkara G, Dash B, Deshmukh A, deSoouza A, Gulia A, Maheshwari A, Moiyadi A, Nair D, Nair NS, Niyogi D, Pal M, Pandey D, Patkar S, Poddar P, Pramesh CS*, Puri A, Saklani A, Shetty P, Shrikhande SV, Shylasree TS, Singh V, Thiagarajan S (Tata Memorial Hospital, Mumbai).

Indonesia: Islam AA*, Kembuan G, Pajan H (Rsud Wahidin Sudirohusodo, Makassar).

Iran: Rahim F (Baghaei Hospital, Ahvaz); Brouki Milan P*, Mozafari M, Rezaei Tavirani M, Tizmaghz A (Firoozabadi Hospital, Tehran); Safari H (Golestan Hospital, Ahvaz).

Ireland: Aremu M*, Canas-Martinez A, Cullivan O, Murphy C, Owens P, Pickett L (Connolly Hospital Blanchardstown, Dublin); Akmenkalne L, Byrne J, Corrigan M*, Cullinane C, Daly A, Fleming C*, Jordan P, Kayyal MY, Killeen S, Lynch N, McCarthy A, Mustafa H, O’Brien S, O’Leary P, Syed WAS, Vernon L (Cork University Hospital, Cork); O'Duffy F, McHugh A*, Moran T (Mater Misericordiae University Hospital, Dublin); Callanan D, Dias A, Huang L, Ionescu A, Sheahan P* (South Infirmary Victoria University Hospital, Cork); Balasubramanian I, Boland M, Carrington E, Conlon K, Cullinane C, Evoy D, Fagan J, Fearon N, Gallagher T, Geary E, Geraghty J, Hanly A, Heneghan H*, Kennedy N, Kennelly R, Maguire D, Martin ST, McCartan D, McDermott EW, McPartland D, Ng KC, Prichard RS, Stafford T, Winter D* (St Vincent’s University Hospital, Dublin); Alazawi D, Barry C*, Boyle T, Butt W, Connolly E, Donlon N, Donohue C, Fahey BA, Farrell R, Fitzgerald C, Kinsella J, Larkin J*, Lennon P, Maguire PJ*, Mccormick P, Mehigan BJ, Mohan H, Nugent TS, O’Sullivan H, Ravi N, Reynolds JV*, Rogers A, Shokuhi P, Smith J, Smith LA, Timon C (St. James’s Hospital, Dublin); Bashir Y, Bass G, Conlon K, Connelly T, Creavin B, Earley H, Elliott JA*, Gillis A, Kavanagh D, Madden A, Manecksha RP, Neary P, O’Connell C, O’riordan J, Reynolds IS, Rice D, Ridgway P, Thomas A, Umair M, Whelan M (Tallaght Hospital, Dublin); Carroll P, Collins C, Corless K, Finnegan L, Fowler AL, Hogan A, Kerin M, Lowery A*, McAnena P, McKevitt K*, Nugent E, Ryan É (University Hospital Galway, Galway); Coffey JC, Cunningham RM, Devine M, Nally DM*, Peirce C, Tormey S (University Hospital Limerick, Limerick); Hardy N, Neary P, O’Malley S*, Ryan M (University Hospital Waterford/University College Cork, Waterford).

Israel: Gaziants V, Gold- Deutch R, Lavy R, Kalmovich-Muallem L, Zmora O* (Shamir Medical Center, Be’er Ya’akov).

Italy: Macina S* (Asst Mantua, Mantova); Mariani NM*, Opocher E, Pisani Ceretti A (Asst Santi Paolo E Carlo, Milan); Ferrari F*, Odicino F, Sartori E* (Asst Spedali Civili, Ospedale Di Brescia, Brescia); Cotsoglou C*, Granieri S (Asst Vimercate, Vimercate); Bianco F*, Camillo’ A, Colledan M*, Tornese S, Zambelli MF (Asst-Papa Giovanni Xxiii- Bergamo, Bergamo); Bissolotti G, Fusetti S, Lemma F* (Azienda Ospedaliera Di Padova, Padova); Marino M*, Marino MV*, Mirabella A, Vaccarella G (Azienda Ospedaliera Ospedali Riuniti Villa Sofia-Cervello, Palermo, Palermo); Sena G* (Azienda Ospedaliera Pugliese-Ciaccio Di Catanzaro, Catanzaro); Agostini C, Alemanno G, Bartolini I, Bergamini C, Bruscino A, Checcucci C, De Vincenti R, Di Bella A, Fambrini M, Fortuna L, Maltinti G, Muiesan P*, Petraglia F, Prosperi P*, Ringressi MN, Risaliti M, Sorbi F*, Taddei A*, Tucci R (Azienda Ospedaliera Universitaria Careggi, Firenze); Bassi C, Bortolasi L*, Campagnaro T*, Casetti L, Conci S, De Pastena M, Esposito A, Fontana M, Guglielmi A, Landoni L, Malleo G, Marchegiani G, Nobile S, Paiella S, Pedrazzani C, Rattizzato S, Ruzzenente A, Salvia R*, Turri G, Tuveri M (Azienda Ospedaliera Universitaria Integrata Di Verona, Verona); Altomare DF, Papagni V, Picciariello A* (Azienda Ospedaliero Universitaria Consorziale Policlinico Di Bari, Bari); Bellora P, D’Aloisio G, Ferrari M, Francone E, Gentilli S*, Nikaj H (Azienda Ospedaliero Universitaria Maggiore Della Carità, Novara); Andreani L, Bianchini M, Capanna R, Caretto M, Chiarugi M, Coccolini F, Cremonini C, Di Franco G, Domenici L*, Furbetta N, Gadducci A, Garibaldi S, Gianardi D, Giannini A, Guadagni S, Morelli L*, Musetti S, Palmeri M, Perutelli A, Simoncini T*, Tartaglia D* (Azienda Ospedaliero Universitaria Pisana, Pisa); Anania G*, Carcoforo P*, Chiozza M, De Troia A, Koleva Radica M, Portinari M, Sibilla MG, Urbani A (Azienda Ospedaliero Universitaria Sant’anna, Ferrara); Fabbri N, Feo CV*, Gennari S, Parini S, Righini E (Azienda Unità Sanitaria Locale di Ferrara - University of Ferrara); Ampollini L*, Arcuri MF, Bellanti L, Bergonzani M, Bertoli G, Bocchialini G, Cattelani L*, D’Angelo G*, Gussago F, Lanfranco D, Manigrasso E, Musini L, Poli T, Polotto S, Santoro GP, Varazzani A* (Azienda Ospedaliero-Universitaria Di Parma, Parma); Aguzzoli L, Annessi V, Borgonovo G, Castro Ruiz C, Coiro S, Falco G*, Mandato VD*, Mastrofilippo V, Montella MT, Zizzo M* (Azienda Unità Sanitaria Locale - IRCCS Di Reggio Emilia, Reggio Emilia); Grossi U, Novello S, Romano M, Rossi S, Zanus G* (Ca’ Foncello Treviso, Università Di Padova); Esposito G, Frongia F, Pisanu A, Podda M* (Cagliari University Hospital, Cagliari); Belluco C, Lauretta A*, Montori G, Moras L, Olivieri M (Centro Di Riferimento Oncologico Di Aviano (Cro) Irccs, Aviano); Bussu F, Carta AG, Cossu ML, Cottu P, Fancellu A, Feo CF, Ginesu GC, Giuliani G, Madonia M, Perra T*, Piras A, Porcu A*, Rizzo D, Scanu AM, Tedde A, Tedde M (Cliniche San Pietro, A.o.u. Sassari, Sassari); Aversano A, Carbone F, Delrio P*, Di Lauro K, Fares Bucci A, Rega D*, Spiezio G (Colorectal Surgical Oncology Unit- Istituto Nazionale Tumori Fondazione, Pascale-I.r.c.c.s., Naples); Pirozzolo G*, Recordare A, Vignotto C (Dell’angelo Hospital, Venezia); Badalamenti G, Campisi G, Cordova A, Franza M, Maniaci G, Rinaldi G, Toia F* (Department Of Surgical, Oncological And Oral Sciences. University Of Palermo, Palermo); Calabrò M*, Farnesi F, Lunghi EG, Muratore A*, Pipitone Federico NS (Edoardo Agnelli, Pinerolo); D’Andrea G, Familiari P*, Picotti V (Fabrizio Spaziani, Frosinone 03100); De Palma G, Luglio G*, Pagano G, Tropeano FP (Federico II University Hospital, Naples); Antonelli B, Baldari L*, Beltramini GA, Boni L*, Cassinotti E*, Gianni’ A*, Pignataro L*, Rossi G*, Torretta S (Fondazione IRCCS Ca’ Granda - Ospedale Maggiore Policlinico, Milan); Abatini C, Baia M, Biasoni D, Bogani G, Cadenelli P, Capizzi V*, Cioffi SPB, Citterio D*, Comini LV, Cosimelli M, Fiore M*, Folli S, Gennaro M, Giannini L*, Gronchi A, Guaglio M*, Macchi A*, Martinelli F*, Mazzaferro V, Mosca A, Pasquali S, Piazza C, Raspagliesi F, Rolli L*, Salvioni R, Sarpietro G, Sarre C, Sorrentino L (Fondazione IRCCS Istituto Nazionale dei Tumori - Milan); Agnes A, Alfieri S, Belia F, Biondi A, Cauteruccio M, Cozza V, D’Ugo D, De Simone V, Fagotti A*, Gasparini G, Gordini L*, Litta F, Lombardi CP, Lorenzon L, Maccauro G, Marra AA, Marzi F, Moro A, Parello A, Perrone E, Persiani R, Ratto C, Rosa F, Saponaro G, Scambia G*, Scrima O, Sganga G, Tudisco R, Vitiello R, Ziranu A (Fondazione Policlinico Universitario Agostino Gemelli IRCCS, Rome); Belli A*, Granata V, Izzo F, Palaia R, Patrone R (Hpb Surgical Oncology Unit - Istituto Nazionale Tumori Fondazione, Pascale-I.r.c.c.s., Naples); Carrano FM, Carvello MM, De Virgilio A, Di Candido F, Ferreli F, Gaino F, Mercante G*, Rossi V, Spinelli A*, Spriano G (Humanitas Clinical And Research Center – Irccs, Rozzano (Mi) & Humanitas University, Department Of Biomedical Sciences, Pieve Emanuele, Milan); De Nardi C (Iov - Istituto Oncologico Veneto, Padova); Donati DM*, Frisoni T, Palmerini E (Irccs Istituto Ortopedico Rizzoli, Bologna); Aprile A, Barra F*, Batistotti P, Ferrero S, Fregatti P*, Massobrio A, Pertile D, Scabini S*, Soriero D, Sparavigna M (Irccs Ospedale Policlinico San Martino, Genoa); Adamoli L, Ansarin M*, Cenciarelli S, Chu F, De Berardinis R, Fumagalli Romario U*, Mastrilli F, Pietrobon G, Tagliabue M (Istituto Europeo Di Oncologia - Irccs -Milano, Milan); Badellino E, Biglia N, Chiado’ Piat F, Ferrero A*, Massobrio R (Mauriziano Hospital Torino, Italy); De Manzoni Garberini A* (Ospedale Civile Spirito Santo, Pescara); Mazzotti F*, Pasini F, Ugolini G (Ospedale Degli Infermi Di Faenza, Faenza); Barone R, Birolo SL*, Caccetta M, Deirino A, Garino M, Grasso M, Marafante C, Masciandaro A, Moggia E, Mungo S, Murgese A, Raggio E (Ospedale Degli Infermi Di Rivoli, Rivoli); Federico P, Maida P, Marra E, Marte G, Petrillo A, Tammaro T, Tufo A* (Ospedale Del Mare, Naples); Berselli M*, Borroni G*, Cocozza E, Conti L, Desio M, Livraghi L*, Marchionni V, Quintodei V, Rizzi A (Ospedale Di Circolo, University Of Insubria, University Hospital Of Varese, Asst Sette Laghi, Regione Lombardia, Varese Lombardy); Baldi C*, Corbellini C, Sampietro GM (Ospedale Di Rho - Asst Rhodense, Rho); Bordoni P, Clarizia G, Fleres F*, Franzini M, Grechi A, Longhini A, Spolini A (ASST Valtellina e Alto Lario, Sondrio Hospital, Chirurgia Generale - Sondrio); Cellerino P*, Galfrascoli E, Iacob G (Ospedale Fatebenefratelli E Oftalmico, Milan); Baldini E*, Capelli P, Conti L, Isolani SM, Ribolla M (Ospedale Guglielmo Da Saliceto – Piacenza, Piacenza); Bondurri A, Colombo F*, Ferrario L, Guerci C, Maffioli A (Ospedale Luigi Sacco Milano, Milan); Armao T, Ballabio M*, Bisagni P, Gagliano A, Longhi M, Madonini M, Pizzini P (Ospedale Maggiore Di Lodi, Lodi); Baietti AM, Biasini M, Maremonti P, Neri F, Prucher GM*, Ricci S, Ruggiero F, Zarabini AG (Ospedale Maggiore/Bellaria Carlo Alberto Pizzardi Ausl Bologna, Bologna); Impellizzeri H, Inama M*, Moretto G (Ospedale Pederzoli, Verona); Barmasse R, Mochet S*, Morelli L, Usai A (Ospedale Regionale Umberto Parini, Aosta); Bianco F*, Incollingo P (Ospedale S. Leonardo - Asl Napoli 3 Sud, Castellammare Di Stabia, Naples); Giacometti M*, Zonta S (Ospedale San Biagio, Asl Vco, Domodossola); Marino Cosentino L*, Sagnotta A* (Ospedale San Filippo Neri, Rome); Dell’Oro C, Fruscio R*, Grassi T, Negri S, Nespoli LC, Tamini N*, Zambetti B (Ospedale San Gerardo, Monza); Anastasi A, Bartalucci B, Bellacci A, Canonico G*, Capezzuoli L, Di Martino C, Ipponi P, Linari C, Montelatici M, Nelli T, Spagni G, Tirloni L, Vitali A (Ospedale San Giovanni Di Dio, Firenze); Abate E, Casati M*, Casiraghi T, Laface L, Schiavo M (Ospedale Vittorio Emanuele III - Carate Brianza, Carate Brianza (Mb)); Arminio A, Cotoia A, Lizzi V*, Vovola F (Ospedali Riuniti Azienda Ospedaliera Universitaria Foggia, Foggia); Vergari R* (Ospedali Riuniti Di Ancona, Ancona); D’Ugo S*, Depalma N, Spampinato MG (UOC Chirurgia Generale, Ospedale “Vito Fazzi”, Lecce); Annicchiarico A, Catena F*, Giuffrida M, Perrone G (Parma University Hospital, Parma); Baronio G, Carissimi F, Montuori M, Pinotti E* (Policlinico San Pietro, Ponte San Pietro); Bartolucci P, Binda B, Brachini G, Bruzzaniti P, Chiappini A, Chiarella V, Ciccarone F, Cicerchia PM, Cirillo B, Crocetti D, De Toma G, Di bartolomeo A, Duranti G, Fiori E, Fonsi GB, Franco G, Frati A, Giugliano M, Iannone I, La Torre F, Lapolla P*, Leonardo C, Marruzzo G, Meneghini S, Mingoli A, Ribuffo D, Salvati M, Santoro A, Sapienza P, Scafa AK, Simonelli L, Zanacana G, Zambon M, Zuppi E (Policlinico Umberto I Sapienza University Of Rome, Rome); Capolupo GT*, Carannante F, Caricato M*, Mascianà G, Mazzotta E (Policlinico Universitario Campus Bio Medico Of Rome, Rome); Gattolin A, Migliore M, Rimonda R, Sasia D*, Travaglio E (Regina Montis Regalis Hospital, Mondovì); Chessa A*, Fiorini A, Norcini C (San Giovanni Di Dio, Orbetello); Colletti G, Confalonieri M, Costanzi A*, Frattaruolo C, Mari G, Monteleone M (San Leopoldo Mandic, Merate (Lc)); Bandiera A, Bocciolone L, Bonavina G, Candiani M*, Candotti G, De Nardi P*, Gagliardi F, Medone M, Mortini P*, Negri G*, Parise P, Piloni M, Sileri P, Vignali A (San Raffaele Scientific Institute, Milan, Milan); Belvedere A, Bernante P, Bertoglio P, Boussedra S, Brunocilla E, Cervellera M, Cescon M, Cipriani R, Cisternino G, De Crescenzo E, De Iaco P*, Del Gaudio M, Dondi G, Droghetti M, Germinario G, Gori A, Frio F*, Jovine E, Mineo Bianchi F, Morezzi D, Neri J, Parlanti D, Perrone AM, Pezzuto AP, Pignatti M*, Pinto V, Poggioli G, Ravaioli M, Rottoli M*, Russo IS, Sartarelli L, Schiavina R, Serenari M*, Serra M, Solli P*, Tonini V, Taffurelli M*, Tanzanu M, Tesei M, Violante T, Zanotti S (IRCCS Azienda Ospedaliero - Universitaria di Bologna); Borghi F, Cianflocca D, Di Maria Grimaldi S, Donati D, Gelarda E, Geretto P, Giraudo G, Giuffrida MC, Maione F, Marano A*, Palagi S, Pellegrino L, Peluso C, Giaccardi S, Testa V* (Santa Croce E Carle Hospital, Cuneo, Cuneo); Agresta F*, Prando D*, Zese M* (Santa Maria Degli Angeli Hospital Ulss5 - Adria, Adria); Aquila F, Gambacciani C, Lippa L, Pieri F, Santonocito OS* (Spedali Riuniti Di Livorno, Livorno); Armatura G*, Bertelli G, Frena A, Marinello P, Notte F, Patauner S, Scotton G* (St. Moritz Hospital, Bolzano); Fulginiti S, Gallo G*, Sammarco G, Vescio G (University ‘Magna Graecia’ Of Catanzaro, Catanzaro); Balercia P, Catarzi L, Consorti G* (University Hospital Umberto I Ancona, Ancona); Asti ELG, Bernardi D, Bonavina L*, Lovece A (University Of Milan, Irccs Policlinico San Donato, San Donato); Di Marzo F* (Valtiberina, Sansepolcro).

Japan: Fujiwara H* (Jichi Medical University Hospital, Shimotuske); Hashimoto D*, Yamaki S, Yamamoto T (Kansai Medical University, Hirakata); Daiko H*, Ishikawa M, Ishiyama K, Iwata S, Kanematsu K, Kanemitsu Y*, Kato T*, Kawai A*, Kobayashi E, Kobayashi Kato M, Moritani K, Nakagawa M, Nakatani F, Oguma J, Tanase Y, Uno M (National Cancer Center Hospital, Tokyo); Hada T, Iwahashi H, Miyamoto M, Suminokura J, Takano M* (National Defense Medical College, Tokorozawa); Fujiwara K*, Fujiwara N, Kurosaki A (Saitama Medical University International Medical Center, Hidaka-City).

Jordan: Ababneh H, Al Abdallah M*, Ayasra F, Ayasra Y, Hammad F, Qasem A (Al-Basheer Hospital, Amman); Abu Za’nouneh FJ, Al-Shraideh AA, Fahmawee T, Hmedat A, Ibrahim A, Obeidat K* (King Abdullah University Hospital, Ar Ramtha); Abdel Al S, Abdel Jalil R, Abou Chaar MK, Al-Masri M*, Al-Najjar H, Alawneh F, Alsaraireh O, Elayyan M, Ghanem R, Lataifeh I (King Hussein Cancer Center, Amman).

Kazakhstan: Fakhradiyev I*, Saliev T, Tanabayeva S (City Clinical Hospital No. 4, Almaty).

Kuwait: Almahmeed H*, Almazeedi S*, Alsabah S*, Jamal M* (Jaber Al Ahmad Al Sabah Hospital, Kuwait).

Libya: Aldokali N, Senossi O, Subhi MT (Alkhadra Hospital, Tripoli); Algallai M, Alwarfly S, AlZAEDE S, Gahwagi M*, Moftah M (Benghazi Medical Center, Benghazi); Abusannoga M, Alawami A, Alawami M*, Albashri M, Malek A (Medical Care Clinic, Tripoli); Burgan D*, Kamoka E, Kilani AI, Salamah A, Salem M, Shuwayyah A (National Cancer Institute, Sabratha - Libya, Sabratha); Abdelkabir M*, Altomi I, Altoumi M (Sabha Medical Center, Tripoli); Bouhuwaish A*, Elmabri A, Omar M, Taher AS (Tobruk Medical Center, Tobruk); Abdulwahed E*, Alshareea E, Aribi N, Aribi S, Biala M, Ghamgh R, Morgom M (Tripoli Central Hospital, Tripoli); Alansari SAA, Aldayri Z, Alsoufi A, Elhadi A, Elhajdawe F, Ellojli I*, ERgebi AAD, Kredan A, Msherghi A*, Nagib T (Tripoli University Hospital, Tripoli); Abudher A*, Alshareef K*, Elamin F (Yashfeen Clinic, Tajora-Tripoli).

Lithuania: Bradulskis S, Dainius E, Kubiliute E, Kutkevičius J, Parseliunas A, Subocius A, Venskutonis D* (Lithuanian University Of Health Sciences Kaunas Clinical Hospital, Kaunas).

Madagascar: Rasoaherinomenjanahary F*, Razafindrahita JB, Samison LH (Joseph Ravoahangy Andrianavalona Hospital, Antananarivo).

Malaysia: Ong EC (Bintulu, Bintulu); Abdul Maei N, Ngo CW*, Ramasamy S (Hospital Enche’ Besar Hajjah Khalsom, Kluang, Johor); Hamdan KH, Ibrahim MR, Tan JA, Thanapal MR* (Hospital Kuala Lumpur, Kuala Lumpur); Choong E, Lim RZM* (Hospital Sultanah Aminah, Johor); Amin Sahid N, Hayati F*, Jayasilan J, Sriram RK*, Subramaniam S (Queen Elizabeth Hospital & Universiti Malaysia Sabah, Kota Kinabalu, Sabah, Malaysia, Kota Kinabalu); Ibrahim AF* (Sarawak General Hospital, Kuching, Sarawak); Che jusoh A, Hussain AH, Mohamed Sidek AS, Mohd Yunus MF, Soh JY, Wong MP, Zakaria AD*, Zakaria Z (School Of Medical Sciences & Hospital, Universiti Sains Malaysia, Kelantan); Kampan N, Mohd Azman ZA, Nur Azurah AG, Zainuddin AA (Universiti Kebangsaan Malaysia Medical Centre, Malaysia); Fadzli AN*, Fathi NQ, Koh PS*, Liew YT*, Roslani AC*, Tang CY, Teoh LY*, Wong WJ*, Xavier R, Yahaya AS (University Malaya Medical Centre, Kuala Lumpur).

Mexico: Alvarez MR, Arrangoiz R, Cordera F*, De la Rosa Abaroa MA, Gómez-Pedraza A, Hernandez R, Maffuz-Aziz A, Posada JA (Abc Medical Center, Mexico City); Lupián-Angulo AI, Soulé Martínez CE* (Hospital Central Norte Pemex, Mexico City); Aboharp Hasan Z, ALvarado Silva C, Bazan Soto A, Hernández Rubio A, Jiménez Villanueva X, Otoniel LR, Sosa Duran EE* (Hospital Juárez De México, Ciudad De México); Becerra García FC* (Hospital San Angel Inn Patriotismo, Mexico City); Melchor-Ruan J*, Romero Bañuelos E, Vilar-Compte D (Instituto Nacional De Cancerologia, Mexico City); Alfaro- Goldaracena A, Buerba GA, Castillejos-Molina RA, Chan C, Dominguez-Rosado I, Medina-Franco H, Mercado MÁ*, Oropeza-Aguilar M, Peña Gómez Portugal E, Posadas-Trujillo OE, Rodriguez-Covarrubias F, Salgado-Nesme N, Sarre C, Vilatoba M (Instituto Nacional De Ciencias Médicas Y Nutrición “Salvador Zubirán”, Mexico City).

Morocco: Arkha Y, Bechri H, El Ouahabi A, Oudrhiri MY* (Centre Hospitalier Universitaire Ibn Sina Rabat, Rabat); El Azhari A, Louraoui SM*, Rghioui M (Cheikh Khalifa International University Hospital, Casablanca City); Bougrine M, Derkaoui hassani F*, El abbadi N (Cheikh Zaid International University Hospital, Rabat); Amrani L, Belkhadir ZH, Benkabbou A, Chakib O, El Ahmadi B, El Bouazizi Y, Essangri H, Ghannam A*, Majbar AM, Mohsine R, Souadka A* (Institut National D’oncologie, Rabat).

Netherlands: Borgstein ABJ, Gisbertz SS*, Van Berge Henegouwen MI* (Amsterdam UMC, Cancer Center Amsterdam, University of Amsterdam); Hompes R*, Meima-van Praag EM, Pronk A, Sharabiany S (Amsterdam Umc, University Of Amsterdam, Amsterdam); Grotenhuis B*, Hartveld L, Reijers S, Van Houdt W* (Antoni Van Leeuwenhoek Ziekenhuis, Amsterdam); Baaij J, Bolster-van Eenennaam M*, De Graaff M, Sloothaak D, Van Duijvendijk P* (Gelre Ziekenhuis, Apeldoorn); Ebben LDA, Kuiper SZ*, Melenhorst J, Poeze M, Sluijpers NRF, Vaassen LAA (Maastricht University Medical Centre +, Maastricht); Posma- Bouman L* (Slingeland Ziekenhuis, Doetinchem); Derksen T, Franken J, Oosterling S* (Spaarne Gasthuis, Haarlem); De Bree R* (University Medical Center Utrecht, Utrecht); Konsten J*, Van Heinsbergen M (Viecuri Medisch Centrum, Venlo).

Nigeria: Adeyeye A, Akinmade A*, Enoch E, Fayose S (Afe Babalola University Multi-System Hospital, Ido Ekiti); Abur P, Fidelis L, Nwabuoku SE, Oyelowo N, Sholadoye TT*, Tolani MA* (Ahmadu Bello University Teaching Hospital, Zaria); Olaogun J* (Ekiti State University Teaching Hospital, Ado-Ekiti); Abiyere H, Adebara I, Adeniyi A, Adeyemo O, Babalola O, Bakare A, Banjo O, Okunlola A* (Federal Teaching Hospital, Ado Ekiti, Ado Ekiti); Adeniran A, Atobatele K, Eke G, Faboya O, Ogunyemi A, Omisanjo O, Oshodi O, Oshodi Y, Williams O* (Lagos State University Teaching Hospital, Ikeja); Ademuyiwa A, Afolabi B, Akinajo O, Alakaloko F, Atoyebi O, Balogun O, Belie O, Bode C, Chibuike George I, Elebute O, Ladipo- Ajayi O, Ohazurike E, Okunowo A, Olajide TO, Seyi-Olajide J* (Lagos University Teaching Hospital, Idi Araba); Daniel A, Egbuchulem IK*, Lawal TA*, Nwaorgu O*, Ogundoyin O*, Olulana D, Onakoya P, Oyelakin O (University College Hospital, Ibadan); Abdullahi H, Agida E, Aisuodionoe-Shadrach O, Ajibola H, Akaba G, Sani AS, Chinda J, Dawang Y, Garba S, Mshelbwala P, Obande J, Olori S*, Olute A, Osagie O, Pius Ogolekwu I, Umar A (University Of Abuja Teaching Hospital, Gwagwalada); Abdur-Rahman L*, Adeleke N, Adeyeye A*, Aremu I, Bello J, Olasehinde O, Popoola A, Raji HO (University Of Ilorin Teaching Hospital, Ilorin).

Oman: Massoud JG*, Massoud R, Sorour TM (Khoula Hospital, Muscat).

Pakistan: Abassy J*, Ahmed K, Alvi A, Arshad M, Khan S*, Pirzada A, Saleem A, Siddiqui T, Turk K (Aga Khan University, Karachi); Hanif F*, Haroon M, Khan MI (Bahria International Hospital, Bahria Orchard, Lahore); Jamal A, Kerawala AA* (Cancer Foundation Hospital, Karachi); Memon AS*, Nafees Ahmed R, Rai L* (Dr Ruth K.m. Pfau Civil Hospital, Karachi); Javed S*, Mahmood U, Shabbir RK, Yaqoob E* (Holy Family Hospital, Rawalpindi); Afzal A*, Ahmed Riaz S, Akbar A, Ali AA*, Ali G, Janjua A*, Mohsin M*, Naqi SA*, Saleem I*, Shaukat A*, Sohail M (King Edward Medical University, Mayo Hospital, Lahore, Lahore); Afzal MF, Khokhar MI*, Latif F (Lahore General Hospital, Pgmi, Amc, Lahore); Ayub B, Hassan N*, Martins RS, Ramesh P, Sayyed R* (Patel Hospital, Karachi); Ayyaz M*, Butt U*, Kashif M, Khan WH*, Qureshi AU*, Umar M, Waris Farooka M*, Wasim T* (Services Hospital Lahore, Lahore); Bhatti ABH* (Shifa International Hospital, Islamabad); Ayubi A, Rashid I, Waqar SH* (The Pakistan Institute Of Medical Sciences, Islamabad).

Palestine: Al-Slaibi I, I. A. Alzeerelhouseini H, Jobran F* (Al-Ahli Hospital, Hebron, West Bank); Abukhalaf SA (Palestine Medical Complex, Ramallah, West Bank).

Panama: Arrue E, Cukier M*, Rodriguez-Zentner H (Pacifica Salud Hospital, Panama).

Peru: Borda-Luque G*, León Palacios JL, Lizzetti G, Vasquez Ojeda XP (Cayetano Heredia National Hospital, Lima); Falcon Pacheco GM*, Robles R (Instituto Regional De Enfermedades Neoplásicas Del Sur, Arequipa).

Philippines: Jocson R, Teh C*, Uy Magadia E (National Kidney & Transplant Institute, Quezon City).

Poland: Major P (Jagiellonian University Medical College, Krakow); Bąk M, Dubieńska K, Ławnicka A, Murawa D* (Karol Marcinkowski University Hospital, Zielona Gora); Bobiński M*, Kotarski J*, Rasoul- Pelińska K* (Medical University Of Lublin, Ist Chair And Department Of Gynaecological Oncology And Gynaecology, Lublin); Brociek A, Chloupek A, Janik M, Kowalewski P, Kwiatkowski A, Panasiewicz P, Roszkowski R, Rot P, Sroczyński P, Walędziak M* (Military Institute Of Medicine, Warsaw).

Portugal: Azevedo C, Machado D, Mendes F* (Centro Hospitalar Cova Da Beira, Covilha); De Sousa X* (Centro Hospitalar De Setúbal, Setúbal); Fernandes U, Ferreira C*, Guidi G, Leal C, Marçal A, Marques R, Martins D, Melo A, Tenreiro N, Vaz Pereira R, Vieira B (Centro Hospitalar De Trás-Os-Montes E Alto Douro, E.p.e., Vila Real); Almeida JI, Almeida-Reis R*, Correia de Sá T, Costa MJMA, Fernandes V, Ferraz I, Lima da Cruz L, Lima da Silva C, Lopes L, Machado N, Marialva J, Nunes Coelho M, Pedro J, Pereira C, Ribeiro A, Ribeiro CG, Santos R, Saraiva P, Silva RL, Tavares F, Teixeira M, Valente P (Centro Hospitalar Do Tamega E Sousa, Penafiel); Almeida AC, Amaral MJ, Andrade R, Athayde Nemésio R, Breda D, Camacho C, Canhoto C, Colino M, Correia S, Costa M, De Barros J, De Oliveira López AL, Duque M, Garrido S, Guerreiro P, Guimarães A, Lázaro A*, Lopes C, Martins R, Nogueira O, Oliveira A, Oliveira JM, Rodrigues M, Ruivo A, Santos E, Silva M, Simões J, Valente da Costa A (Centro Hospitalar E Universitário De Coimbra, Coimbra); Almeida A, Castanheira Rodrigues S, Cavaleiro Leitão de Carvalho AS, Devezas V, Faria CS, Jácome F, Magalhães Maia M, Nogueiro J, Pereira A, Pereira-Neves A, Pina-Vaz T, Santos- Sousa H*, Silveira H, Vaz S, Vieira P (Centro Hospitalar E Universitário De São João, Porto); Gomes da Costa A, Lobo Antunes I* (Centro Hospitalar Lisboa Norte, Lisbon); Pinto J, Tojal A* (Centro Hospitalar Tondela-Viseu, Viseu); Cardoso N, Cardoso P*, Domingues JC, Henriques P, Manso MI, Martins dos Santos G, Martins R, Morais H*, Pereira R, Revez T, Ribeiro R, Ribeiro VI, Soares A, Sousa S, Teixeira J (Centro Hospitalar Universitário Do Algarve - Unidade De Faro, Faro); Amorim E, Baptista VH, Cunha MF*, Dias B, Fazenda A, Melo Neves JP, Policarpo F, Sampaio da Nóvoa Gomes Miguel II, Veiga D (Centro Hospitalar Universitario Do Algarve - Unidade De Portimão, Portimao); Bandovas JP, Borges N*, Branquinho A, Chumbinho B, Correia J, Fidalgo H, Figueiredo de Barros I, Frade S, Gomes J, Kam da Silva Andrade A, Maciel J, Pereira Rodrigues A, Pina S, Silva N*, Silveira Nunes I, Sousa R (Centro Hospitalar Universitário Lisboa Central, Lisbon); Ascensão J, Azevedo P, Costeira B, Cunha C, Garrido R*, Gomes H, Lourenço I, Mendinhos G*, Miranda P, Nobre Pinto A, Peralta Ferreira M, Ribeiro J, Rio Rodrigues L, Sousa Fernandes M (Hospital Beatriz Angelo, Loures); Azevedo J* (Hospital Da Horta, E.p.e., Horta); Galvão D, Soares AC, Vieira A*, Vieira B (Hospital De Santo Espirito Da Ilha Terceira, Angra Do Heroismo); Patrício B, Santos PMDD*, Vieira Paiva Lopes AC (Hospital De Torres Vedras - Centro Hospitalar Do Oeste, Torres Vedras); Cunha R, Faustino A, Freitas A, Jacob Oliveira B, Martins AB, Mendes JR*, Parreira R, Rosa J, Teves M (Hospital Do Divino Espírito Santo, Ponta Delgada); Abreu da Silva A*, Claro M, Costa Santos D, Deus AC, Grilo JV (Hospital Do Litoral Alentejano, Santiago Do Cacém); Castro Borges F*, Corte Real J, Henriques S, Lima MJ, Matos Costa P (Hospital Garcia De Orta, Almada); Alagoa Joao A, Azevedo P, Camarneiro R, Capunge I, Fragoso M, Frazão J, Martins A, Pedro V, Pera R, Ramalho de Almeida F, Sampaio Soares A*, Vale R, Vasconcelos M (Hospital Prof. Doutor Fernando Fonseca, E.p.e., Amadora); Brito da Silva F, Caiado A*, Fonseca F (Instituto Português De Oncologia De Lisboa Francisco Gentil, Lisboa); Ângelo M, Baiao JM, Martins Jordão D*, Vieira Caroço T (Ipo Coimbra, Coimbra); Messias J, Millan A, Salgado I, Santos P* (Ipo Lisboa, Lisboa); Baía C, Canotilho R, Correia AM, Ferreira Pinto AP, Peyroteo M, Videira JF* (IPO Porto, Porto).

Puerto Rico: Escobar P*, Maldonado Santiago M (Instituto Gineco Oncologico, San Juan). Réunion: Kassir R*, Sauvat F (Chu Reunion, Saint Denis).

Romania: Bonci E*, Gata V*, Titu S* (“Prof. Dr. Ion Chiricuta” Institute Of Oncology, Cluj-Napoca); Bezede C, Chitul A, Ciofic E, Cristian D, Grama F* (Coltea Clinical Hospital, Bucharest); Pirtea L*, Secosan C (Emergency Clinical City Hospital, Timisoara); Ciubotaru C, Negoi I*, Negoita VM, Stoica B (Emergency Clinical Hospital Bucharest, Bucharest); Ginghina O*, Iordache N, Iosifescu RV, Mardare M, Mirica RM, Spanu A, Văcărașu AB, Zamfir-Chiru-Anton M (Saint John Emergency Hospital, Bucharest).

Russia: Garmanova T, Kazachenko E, Markaryan D, Rodimov S, Tsarkov P*, Tulina I (Clinic Of Coloproctology And Minimally Invasive Surgery, Sechenov Medical State University, Moscow); Abelevich A, Bazaev A, Kokobelyan AK, Yanishev A* (Privolzhsky Research Medical University, Nizhny Novgorod Regional Clinical Hospital, Sechenov Medical State University, Nizhny Novgorod); Litvin A*, Litvina Y, Provozina A (Immanuel Kant Baltic Federal University, Regional Clinical Hospital, Kaliningrad); Agapov M*, Galliamov E, Kakotkin V, Kubyshkin V, Semina E, Камалов А (Moscow Research And Educational Center, Lomonosov Moscow State University, Moscow); Novikova A, Zakharenko A* (Pavlov First State Medical University Of St. Petersburg, Saint Petersburg).

Saudi Arabia: Alshahrani M*, Alsharif F, Eskander M (Aseer Central Hospital, Abha); Al Raddadi R, Majrashi S*, Mashat A (East Jeddah General Hospital, Jeddah); Akeel N, Alharthi M, Aljiffry M, Basendowah M, Farsi A, Ghunaim M, Khoja A, Maghrabi A, Malibary N*, Nassif M, Nawawi A, Saleem A, Samkari A, Trabulsi N* (King Abdulaziz University Hospital, Jeddah); A Azab M* (King Abdullah Medical City Makkah, Makkah); Aldosri M, Alghanem A, Alguraigari A, ALjohani K, Alqahtani D, Alzaidi TM, Basyouni A, Elhussain E, Jaloun H*, Mudawi I, Shafei M (King Fahad Armed Forces Hospital, Jeddah); Al Awwad S*, Alghamdi M*, Alnumani T*, Nasser M*, Said bayazeed A* (King Fahad General Hospital, Jeddah); Abdelrhman S, Awad S*, Ghedan S, I Sharara M, Mashaly A (King Faisal Medical Complex, Taif City); Aburahmah M, Al Otaibi F, Al-alem I, Al-Badawi IA, AlDahash H, Alhazzaa N, Alhefdhi A*, Alhelal B, AlKattan K, Almalik O, Alomair A, Alomar O, Alotaibi NH, Alresaini F, Alrifai O, Alsakka M, Alsalamah R, Alsemari M, Alsobhi S, AlSumai T, Farrash F, Khan P, Mahasin Z, Othman E, Pant R, Robaidi H, Saleh W, Salem H, Shaheen M, Spangenberg P, Velagapudi S (King Faisal Specialist Hospital, Riyadh); Al Habes H, Alamri A, Alkarak S, Alqannas M*, Alyami M*, Alzamanan M, Cortés-Guiral D*, Elawad A (King Khalid Hospital, Najran); Adi H, Al ahmad F, Al Ayed A, Al zahrani A, Alalawi Y*, Alishi Y, Alqahtani B (King Salman Armed Forces Hospital, Tabouk); AlAamer O, Alriyees L, Alselaim N* (King Saud Bin Abdulaziz University For Health Sciences, King Abdullah International Medical Research Center, Ministry Of National Guard, Health Affairs, General Surgery Department., Riyadh); Alfaifi J, Alkreedees N, Almutrafi SN*, Alramadhan M, Alshitwi A, D’Souza J (King Saud Medical City, Riyadh); Abdulkareem A, Ajlan A, Akkour K, Al-Habib A, Al-Khayal K, Alatar A, Alburakan A, Alhalal H, Alhassan B, Alhassan N, Aljassir F, Alobeed O, Alsaif A, Alsaif F, Alshammari S, Alshaygy I, Barry M, Bin Nasser A*, Bin Traiki T, Bokhari A, Elwatidy S, Helmi H, Madkhali A, Nouh T*, Rabah PD, Zubaidi A (King Saud University, Riyadh); Al Amri A* (Najran University Hospital, Najran); Abdulfattah F, Al Hasan I*, Al-Kharashi E*, Alanazi F, Albaqami F, Alghamdi A, Alghuliga A, Aljaber F, Alsowaina K, Alsuhaibani A, Arab N*, Badahdah F (Prince Sultan Military Medical City, Riyadh); Alobaysi S, Alshahrani A, Alzahrani A* (Security Forces Hospital, Riyadh).

Serbia: Paunovic I*, Slijepcevic N (Centre For Endocrine Surgery, Clinical Centre Of Serbia, Belgrade); Aleksić L, Antic A, Barisic G*, Ceranic M, Ebrahimi K, Galun D*, Grubač Ž, Ivanović N, Jelenkovic J, Kecmanović D, Kmezić S, Knezevic D*, Krivokapic Z*, Latinčić S, Markovic V*, Matić S*, Miladinov M, Pavlov M*, Pejovic I, Radenkovic D*, Sabljak P*, Skrobić O*, Šljukić V, Tadic B, Vasljević J, Velickovic D, Zivanovic M (Clinic For Digestive Surgery, Clinical Centre Of Serbia, Belgrade); Doklestic K, Gregoric P*, Ivancevic N, Loncar Z, Micic D (Clinic For Emergency Surgery, Emergency Centre, Clinical Centre Of Serbia, Belgrade); Perovic M, Srbinovic L (Clinic For Gynecology And Obstetrics Narodni Front, Belgrade); Andrijasevic S, Bozanovic T, Cerovic Popovic R, Dokic M, Janjic T, Jeremic K, Kadija S, Ladjevic Likic I, Mirkovic L, Pantovic S, Pilic I, Radojevic M, Stefanovic A*, Vidakovic S, Vilendecic Z (Clinic For Gynecology And Obstetrics, Clinical Center Of Serbia, Belgrade); Antic S, Dunđerović D, Jelovac D*, Jezdic Z, Konstantinovic V, Kotlar B, Kuzmanovic C, Lazić M, Pajić S, Petrovic M, Popovic F, Pucar A, Romic M, Sumrak S, Vujanac V (Clinic For Maxillofacial Surgery, School Of Dental Medicine, University Of Belgrade, Belgrade); Bascarevic V, Bogdanovic I, Grujičić D*, Ilic R*, Jokovic M, Milićević M, Milisavljević F, Miljković A, Paunovic A, Šćepanović V, Stanimirovic A, Todorovic M (Clinic For Neurosurgery, Clinical Center Of Serbia, Belgrade); Folic M, Jotic A, Krejovic Trivic S, Milovanovic J*, Trivic A (Clinic For Otorhinolaryngology And Maxillofacial Surgery, Clinical Center Of Serbia, Belgrade); Bumbasirevic U*, Dzamic Z, Kajmaković B, Prijović N, Zivkovic M (Clinic Of Urology, Clinical Center Of Serbia, Belgrade); Buta M, Cvetkovic A, Djurisic I, Gacic S, Goran M, Inic Z, Jeftic N, Jevric M, Jokic V, Markovic I*, Milanović M, Nikolic S, Pejnovic L, Savković N, Spurnic I, Stevic D, Stojiljkovic D, Vucic N, Zegarac M (Institute For Oncology And Radiology Of Serbia, Belgrade); Karamarkovic A, Kenic M, Kovacevic B, Krdzic I*, Milutinović V, Savic G (Zvezdara University Medical Center, Belgrade).

Singapore: Chan CW, Lieske B* (National University Hospital, Singapore). Slovakia: Gális B, Šimko K (University Hospital Bratislava, Bratislava).

Slovenia: Cokan A*, Crnobrnja B, Dovnik A, Knez J, Pakiž M (University Medical Centre, Maribor). South Africa: Almgla N*, Bernon M, Boutall A, Cairncross L*, Chinnery G, Herman A, Hilton T, Jonas E, Kloppers C*, Malherbe F, Mugla W*, Nel D, Rayamajhi S, Scriba M, Van Wyngaard T, Vogel J (Groote Schuur Hospital, Cape Town).

Spain: Castaño-Leon AM*, Delgado Fernandez J, Eiriz Fernandez C, Espino Segura-Illa M, Esteban Sinovas O, Garcia Perez D, Gomez P, Jimenez-Roldan L, Lagares A, Moreno-Gomez L, Paredes I, Pérez Núñez A, Sánchez Aniceto G*, Santas Alegret M (12 De Octubre University Hospital, Madrid); Fernández Rodríguez P, Paniagua García Señorans M*, Sanchez-Santos R, Vigorita V (Álvaro Cunqueiro Hospital, Vigo); Acrich E, Baena Sanfeliu E, Barrios O, Golda T*, Santanach C, Serrano-Navidad M, Sorribas Grifell M, Vives RV (Bellvitge University Hospital, Hospitalet De Llobregat); Arce Gil J*, Escolà D, Jiménez A* (Comarcal Alt Penedés, Barcelona); Alcázar JA, Angoso-Clavijo M, Blanco-Antona F, Carabias-Orgaz A, Díaz Maag R, Eguía Larrea M, Esteban Velasco C, Garcia J, García-Plaza AGP, Gonzalez-Muñoz JI, Muñoz-Bellvis L, Parreño-Manchado FC, Sánchez Tocino JM, Sanchez-Casado AB, Trebol J* (Complejo Asistencial Universitario De Salamanca, Salamanca); Hernandez Gutierrez J, Tébar Zamora A* (Complejo Hospitalario De Toledo, Toledo); Sánchez Mozo A* (Complejo Hospitalario Universitario De Albacete, Albacete); Cayetano Paniagua L, Gomez Fernandez L* (Consorci Sanitari De Terrassa, Barcelona); Artigues E, Bernal-Sprekelsen JC*, Catalá Bauset JC, Gilabert-Estellés J (Consorcio Hospital General Universitario, Valencia); Collera P, Diaz Del Gobbo R, Farre Font R, Flores Clotet R, Gómez Díaz CJ*, Guàrdia N, Guariglia CA, Osorio A, Sanchez Jimenez R, Sanchon L, Soto Montesinos C (Fundació Althaia-Xarxa Assistencial Universitària De Manresa, Manresa); Albi Martin B, García Villayzán JE* (Fundación Jimenez Diaz University Hospital, Madrid); Alonso-Lamberti L, Assaf M, Baeza Pintado N, Carabias A, García-Quijada J, Huertas Fernandez MA, Jimenez Miramón J, Jimenez V*, Jover JM, Landeo Agüero SA, Leon R, Martín Salamanca MB*, Pérez Simón V, Ponce S, Rodriguez JL, Salazar A, Valle Rubio A (Getafe University Hospital, Getafe); Aguado H* (Hellín Hospital, Albacete); Aldecoa Ansorregui I, Bravo Infante R, De Lacy FB, Di Somma A*, Díaz-Feijoo B*, Enseñat Nora J*, Fabregas N, Ferrés A, Gil Ibañez B, Gonzalez Sanchez JJ*, Gracia I, Hoyos Castro JA, Lacy AM*, Langdon C, Momblán D, Morales X, Oleaga L, Otero A, Pedrosa L, Poblete Carrizo J, Reyes Figueroa LA, Roldan Ramos P, Rumia-Arboix J, Tercero- Uribe AI, Topczewski TE, Torales J, Torne A, Torné R, Turrado-Rodriguez V*, Valero R, Valverde S (Hospital Clinic Barcelona, Barcelona); Anula R, Avellana R, Camarero Rodríguez E, Catalán Garza V, Dziakova J, García Alonso M, Lasses Martínez B, López Antoñanzas L, Muguerza JM*, Ochagavía S, Peña Soria MJ, Rivera-Alonso D, Saez Carlin P, Sánchez del Pueblo C, Sanz Ortega G, Sanz-Lopez R, Torres A (Hospital Clínico San Carlos, Madrid); Garcés-Albir M*, Lopez F*, Martín-Arévalo J, Moro- Valdezate D*, Pla-Marti V (Hospital Clínico Universitario De Valencia, Valencia); Beltrán de Heredia J, De Andrés-Asenjo B*, Gómez Sanz T, Jezieniecki C, Nuñez Del Barrio H, Ortiz de Solórzano Aurusa FJ, Romero de Diego A, Ruiz Soriano M, Trujillo Díaz J, Vazquez Fernandez A (Hospital Clínico Universitario De Valladolid, Valladolid); Lora-Cumplido P, Sosa MV* (Hospital De Cabueñes, Gijón); Balague C, Ballester E, Moral A, Sánchez López A*, Targarona EM (Hospital De La Santa Creu I Sant Pau, Barcelona); Galvan-Perez A, Gonzalez-Gonzalez E, Minaya Bravo AM*, San Miguel-Mendez C (Hospital Del Henares, Madrid); Alonso de la Fuente N, Cazador Labat M, Cecchini L, Espinosa CA*, Jimenez Toscano M*, López Campillo A, Mancebo G, Martorell P, Munarriz M (Hospital Del Mar, Barcelona); Grau- Talens EJ, Martin-Perez B* (Hospital Don Benito-Villanueva, Don Benito (Badajoz)); Benavides Buleje JA, Carrasco Prats M*, Fernández PV, Fernández-López A*, García Escudero D*, García Porcel VJ, Garcia Soria V*, Giménez Francés C*, González Valverde FM, Gurrea-Almela E, López-Morales P, Marco Garrido A, Martínez Alonso JA, Medina E, Muñoz Camarena JM, Parra Baños PA, Peña Ros E, Ramirez Faraco M, Ruiz-Marín M*, Sanchez Rodriguez C, Valero Soriano M (Hospital General Reina Sofía, Murcia); Allué Cabañuz M, Colsa Gutiérrez P, García Domínguez M, Gimenez Maurel T, Martín Anoro LF, Ponchietti L, Rodriguez Artigas JM, Roldón Golet M*, Utrilla Fornals A (Hospital General San Jorge, Huesca); Estaire Gómez M*, Fernández Camuñas Á, Garcia Santos EP, Jimenez Higuera E, López de la Manzanara Cano CA, Martínez-Pinedo C, Moreno Pérez A, Muñoz-Atienza V, Padilla-Valverde D*, Picón Rodríguez R, Redondo Calvo FJ, Sánchez-García S, Sanchez-Pelaez D (Hospital General Universitario De Ciudad Real, Ciudad Real); Curtis Martínez C, Fernández-Candela A, Sánchez-Guillén L* (Hospital General Universitario De Elche, Elche); Colombari RC, Del valle E, Fernández M, Lozano Lominchar P*, Martín L, Rey Valcarcel C, Steiner MA, Tudela M, Zorrilla Ortúzar J (Hospital General Universitario Gregorio Marañón, Madrid); Alcaide Matas F, García Pérez JM, Troncoso Pereira P* (Hospital Mateu Orfila, Mahon); Blas Laina JL, Cros B, Escartin J*, Garcia Egea J, Nogués A, Talal El-Abur I, Yánez C (Hospital Royo Villanova, Zaragoza); Mora-Guzmán I* (Hospital Santa Bárbara, Puertollano); Cárdenas Puiggrós L* (Hospital Universitari De Girona Dr. Josep Trueta, Girona); Abellán M, Achalandabaso Boira M*, Jorba R, Memba Ikuga R, Olona C, Sales Mallafré R (Hospital Universitari De Tarragona Joan Xxiii, Tarragona); Aguilo O, Cavallé Busquets P, Gavalda Pellice MGP*, Jorda Sole M*, Mateu I, Miralles Curto M, Salinas Peña J (Hospital Universitari Sant Joan, Reus); Fernández Martínez D, García Flórez LJ*, Solar-Garcia L (Hospital Universitario Central De Asturias (Huca), Oviedo); Aragon Achig EJ*, Barbier L, Caja Vivancos P*, Gainza A, García Gutierrez JJ, García-Operé G, Gómez-Suárez J, Jiménez-Jiménez M, Mallabiabarrena Ormaechea G*, Marín H, Martin Playa P*, Melchor Corcóstegui I, Municio-Martín JA, Oñate M, Pascua- Gómez LA, Pesántez Peralta MA*, Prieto Calvo M, Rodriguez Fraga A, Villalabeitia Ateca I* (Hospital Universitario Cruces, Barakaldo); De Andres Olabarria U, Durán Ballesteros M, Fernández Pablos FJ, Ibáñez-Aguirre FJ, Sanz Larrainzar A, Ugarte-Sierra B* (Hospital Universitario De Galdakao, Galdakao- Usansolo); Acosta Mérida MA*, Ortiz López D, Yepes Cano AF (Hospital Universitario De Gran Canaria Doctor Negrín, Las Palmas De Gran Canaria); Correa Bonito A, De la Hoz Rodríguez Á, Delgado Búrdalo L, Di Martino M*, García Sánz I, García Septiem J*, Maqueda González R, Martin-Perez E, Muñoz de Nova JL (Hospital Universitario De La Princesa, Madrid); Calvo Espino P*, Guillamot Ruano P (Hospital Universitario De Móstoles, Móstoles); Colao García L, Díaz Pérez D*, Esteban Agustí E, Galindo Jara P, Gutierrez Samaniego M*, Hernandez Bartolome MA*, Serrano González J (Hospital Universitario De Torrejón De Ardoz, Madrid); Alonso Poza A, Diéguez B, García-Conde M, Hernández-García M, Losada M* (Hospital Universitario Del Sureste, Madrid); Chiesa-Estomba CM, González García JÁ, Larruscain E, Sistiaga-Suárez JA* (Hospital Universitario Donostia, San Sebastian); Alvarez E, Chavarrias N, Frías L, García Pineda V, Gegúndez Simón A, Gómez Rivas J, Gortázar de las Casas S, Gracia M, Guevara J, Hernández Gutierrez A, Loayza A, María Dolores DT, Martí C, Melendez M, Moreno-Palacios E, Perez Y, Prieto Nieto MI, Ramos-Martín P, Rubio-Perez I*, Saavedra J, Sánchez Méndez JI, Siegrist Ridruejo J, Toribio Vazquez C, Urbieta A, Yebes A, Zapardiel I* (Hospital Universitario La Paz, Madrid); Aparicio- López D, Cantalejo diaz M, De Miguel Ardevines MDC, Dobón Rascón MÁ, Duque-Mallén V*, Gascon Ferrer I, González-Nicolás Trébol MT, Gracia-Roche C, Herrero Lopez M, Jariod Ferrer UM*, Kälviäinen H, Lanzon A, Martinez German A, Matute M, Redondo C, Sánchez Fuentes N, Santero-Ramirez MS, Saudí S, Simón Sanz MV, Uson T (Hospital Universitario Miguel Servet, Zaragoza); Blazquez Martin A, Diez Alonso M*, García Rico E, Garcia-Loarte Gomez E, Garcia-Moreno Nisa F, Gutierrez Calvo A, Hernandez P, Lasa I, Mendoza-Moreno F, Morales Palacios N*, Ovejero Merino E, Vera Mansilla C (Hospital Universitario Principe De Asturias, Madrid); Acero J*, Haddad A (Hospital Universitario Ramon Y Cajal, Madrid); Barranquero AG, Caballero Silva U, Cabañero Sánchez A*, Cavestany Garcia-Matres C, Cerro Zaballos C, Fra Fernández S, Moreno Mata N, Muñoz Molina GM, Núñez J, Ocaña J, Ramos D* (Hospital Universitario Ramón y Cajal, Madrid); Acebes García F, Bailón M, Bueno Cañones AD, Choolani Bhojwani E, Marcos- Santos P, Miguel T, Pacheco Sánchez D, Pérez-Saborido B, Sanchez Gonzalez J, Tejero-Pintor FJ* (Hospital Universitario Río Hortega, Valladolid); Alconchel F*, Conesa A, Gil Martínez J*, Gutiérrez Fernández AI, Lopez Abad A, Nicolás-López T*, Ramirez Romero P, Roca Calvo MJ, Rodrigues K*, Ruiz Manzanera JJ, Soriano AI (Hospital Universitario Virgen De La Arrixaca, Murcia); Cano A, Capitan-Morales L, Cintas Catena J, Gomez-Rosado J*, Oliva Mompean F, Pérez Sánchez MA, Río Lafuente FD, Torres Arcos C, Valdes-Hernandez J (Hospital Universitario Virgen Macarena, Seville); Bruna Esteban M, Cholewa H, Domingo S*, Frasson M, Lago V*, Marina Martin T*, Martínez Chicote C, Sancho-Muriel J* (Hospital Universitario Y Politécnico La Fe, Valencia); Estraviz-Mateos B, Fernández Gómez Cruzado L, González de Miguel M, Landaluce-olavarria A*, Lecumberri D (Hospital Urduliz, Bizkaia); Abad Gurumeta A, Abad-Motos A, Martínez-Hurtado E, Ripollés-Melchor J*, Ruiz Escobar A (Infanta Leonor University Hospital, Madrid); Cuadrado-García A*, Garcia-Sancho Tellez L*, Heras Aznar J*, Maté Mate P, Ortega Vázquez I*, Picardo AL, Rojo López JA, Sanchez Cabezudo Noguera F*, Serralta de Colsa D* (Infanta Sofía University Hospital, San Sebastian De Los Reyes); Anchuelo Latorre J, Cagigas Fernandez C, Caiña Ruiz R, Fernandez Diaz MJ, Gomez Ruiz M, Hernanz F, Jimeno Fraile J*, Martínez-Pérez P, Poch C, Santarrufina Martinez S*, Valbuena Jabares V (Marqués De Valdecilla University Hospital, Santander); Moliner-Sachez C, Pingarron-Martin L, Rey-Biel J*, Ruiz Martin I (Rey Juan Carlos University Hospital, Móstoles); Cagigal Ortega EP, Cervera I, Díaz Peña P, Garcia de Castro Rubio E , Enjuto D*, Fernández Bernabé P, Garcés García R, Gonzalez J, Hernández I, Herrera-Merino N, Marqueta De Salas M, Martinez Pascual P, Perez Gonzalez M*, Ramos Bonilla A, Rodríguez Gómez L (Severo Ochoa University Hospital, Leganés); Alfonso Garcia M, Craus-Miguel A, Fernández Vega L, Ferrer-Inaebnit E, Gil Catalán A, González Argente FX, Jeri S, Oseira A, Pujol Cano N, Segura-Sampedro JJ*, Soldevila Verdeguer C, Villalonga B (Son Espases University Hospital, Palma De Mallorca); Bescós C*, Blanco-Colino R, Brana I, Caimari B, De Pablo García-Cuenca A, Duran-Valles F, Espin-Basany E*, Giralt López de Sagredo J, Pamias J, Pellino G, Prat N, Pujol Pina R, Saez barba M (Vall D’hebron University Hospital, Barcelona).

Sri Lanka: Arulanantham A, Bandara GBKD, Jayarajah U*, Ravindrakumar S, Rodrigo VSD (District General Hospital Chilaw, Chilaw).

Sudan: Ali Adil AA (Al-Rajhi, Omdurman); Elhafiz MHY* (Best Care Hospital, Khartoum); Ali EE, Awadelkarim M, Bakheit I*, Elbahri H, Hamid HKS (Ibrahim Malik Teaching Hospital, Khartoum); Essa MEA*, Ahmed AA, Abubakr Hassan, Momin Majed Yousuf Hilles (Medical and Cancer Research Institute at Nyala); Saleh M (University Of Gezira Hospital, Wad Madani).

Sweden: Arkani S*, Freedman J* (Danderyds Hospital, Stockholm); Elbe P*, Lindqvist EK* (Karolinska University Hospital, Stockholm); Angenete E*, Park J, Taflin H* (Sahlgrenska University Hospital, Gothenburg); Greiff L*, Hagander L* (Skane University Hospital, Lund); Älgå A*, Heinius G, Nordberg M, Pieniowski E (South General Hospital, Stockholm); Gkekas I, Löfgren N, Rutegård M*, Sund M* (Umea University Hospital, Umea).

Switzerland: Arigoni M, Bernasconi M, Christoforidis D*, Di Giuseppe M, La Regina D, Mongelli F (Ente Ospedaliero Cantonale, Ticino (Lugano, Bellinzona, Locarno, Mendrisio)); Chevallay M, Dwidar O, Gialamas E, Sauvain M* (Hopital De Pourtales, Neuchatel); Giger R*, Hool S, Klenke F, Kollàr A*, Kurze C, Mueller SA (Inselspital, Bern University Hospital, University Of Bern, Bern); Kiessling S, Stoeckli S* (Kantonsspital St. Gallen, St. Gallen); Adamina M*, Bächler T, Crugnale AS, Giardini M, Guglielmetti L, Peros G, Solimene F (Kantonsspital Winterthur, Winterthur); Gass M*, Metzger J, Scheiwiller A (Luzerner Kantonsspital, Luzern); Gutschow C*, Turina M* (Universitätsspital, Zürich).

Syrian Arab Republic: Al Asadi T, Alkhateb S, Altom R, Bakkar B, Maa Albared S*, Melhem S (Damascus Hospital, Damascus); Hamdan A, Hammed A*, Hammed S, Hossain M, Mahfoud M, Moussa A (Tishreen University Hospital, Latakia); Alsayyad R, Alsrouji S, Ashour G, Hareth Al-Nahr M, Slitin A, Tanos C (Al- Hilal Hospital).

Tunisia: Kacem MJ, Maghrebi H, Sebai A* (La Rabta Hospital, Tunis).

Turkey: Aghayeva A*, Hamzaoglu I, Sahin I (Acibadem Altunizade Hospital, Istanbul); Akaydin E, Aliyeva Z, Aytac E, Baca B, Dülgeroğlu O, Ozben V*, Ozmen BB, Uras C (Acibadem Atakent Hospital, Istanbul); Arikan AE*, Bilgin IA*, Bozkırlı B*, Ceyhan GO, Kara H, Karahasanoğlu T, Uras C (Acibadem Maslak Hospital, Istanbul); Celik H* (Adana Baskent University, Adana); Meydanli MM* (Ankara City Hospital, Ankara); Akbas A, Altinel Y*, Calikoglu F, Ercan G, Ercetin C, Hacım NA, Meriç S, Tokocin M, Vartanoglu T, Yigitbas H (Bagcilar Research And Training Hospital, Istanbul); Akilli H*, Ayhan A*, Kuscu E* (Baskent University, Ankara); Doğangün M, Iflazoğlu N, Yalkın Ö* (Bursa City Hospital, Bursa); Turna A* (Cerrahpasa Medical Faculty Istanbul University, Istanbul); Onan MA* (Gazi University Medical Faculty Hospital, Ankara); Akgor U*, Cennet O, Dincer HA, Erol T, Gultekin M*, Orhan N, Ozgul N*, Salman MC*, Soyak B* (Hacettepe University Hospital, Ankara); Aydemir L, Başaran B, Kara H, Sen C*, Ulusan M (Istanbul University Faculty Of Medicine, Istanbul); Açıkgöz AS, Alhamed A, Aykanat Y, Bese T, Cebi S, Demirkıran F, Ergün S*, Kayan B, OZcelık MF, Sanli AN, Uludağ SS*, Velidedeoglu M*, Zengin AK (Istanbul Universty - Cerrahpaşa Medical Faculty, Istanbul); Bozkurt MA, Kara Y*, Kocatas A (Kanuni Sultan Suleyman Training And Research Hospital, Istanbul); Candas Altinbas B, Çekiç AB, Eyuboglu K, Guner A*, Türkyılmaz S, Usta MA (Karadeniz Technical University, Farabi Hospital, Trabzon); Cimenoglu B, Demirhan R, Saracoglu K* (Kartal Dr. Lutfi Kirdar Training And Research Hospital, Istanbul); Azamat İF, Balik E*, Buğra D, Giray B, Kulle CB, Taskiran C*, Vatansever D (Koç University Medical School, Istanbul); Güler SA, Güreşin A, Tatar OC*, Utkan NZ, Yildirim A, Yüksel E (Kocaeli University Teaching Hospital, Kocaeli); Abbasov A*, Yanar H (Liv Hospital Ulus, Istanbul); Ugurlu MU* (Marmara University, School Of Medicine, Istanbul); Akin E, Altintoprak F*, Bayhan Z, Cakmak G, Çapoğlu R, Çelebi F, Demir H, Dikicier E, Firat N, Gönüllü E, Kamburoğlu MB, Kocer B, Küçük IF, Mantoglu B (Sakarya University Faculty Of Medicine, Sakarya); Çolak E*, Kucuk GO, Uyanik MS (Samsun Training And Research Hospital, Samsun); Goksoy B* (Sehit Prof.dr. İlhan Varank Training And Research Hospital, Istanbul); Bozkurt E, Citgez B, Mihmanli M, Tanal M*, Yetkin G (Sisli Hamidiye Etfal Training And Research Hospital, Istanbul); Akalin M, Arican C, Avci EK, Aydin C, Demirli Atıcı S*, Emiroglu M, Kaya T*, Kebabçı E, Kilinc G, Kirmizi Y, Öğücü H, Salimoğlu S, Sert İ, Tugmen C, Tuncer K, Uslu G, Yeşilyurt D (University Of Health Sciences Tepecik Training And Research Hospital, İzmir); Karaman E*, Kolusarı A (Van Yuzuncu Yil University, Medical Faculty, Van); Yildiz A* (Yildirim Beyazit University Yenimahalle Training And Research Hospital, Ankara); Gultekin FA* (Zonguldak Bulent Ecevit University School Of Medicine Research And Training Hospital, Zonguldak).

Uganda: Lule H*, Oguttu B* (Kampala International University Teaching Hospital, Ishaka). United Arab Emirates: Abdelgalil K* (Tawam Johns Hopkins Hospital, Al-Ain, Abu Dhabi).

United Kingdom: Agilinko J, Ahmeidat A, Barabasz M, Bekheit M*, Cheung LK, Colloc T, Cymes W, Elhusseini M, Gradinariu G, Hannah A, Kamera BS, Mignot G, Shaikh S*, Sharma P (Aberdeen Royal Infirmary, Aberdeen); Abu-Nayla I, Agrawal A*, Al-Mohammad A, Ali S, Ashcroft J, Azizi A, Baker O, Balakrishnan A*, Byrne M, Colquhoun A, Cotter A, Coughlin P, Davies RJ*, Durrani A, Elshaer M, Fordington S, Forouhi P*, Georgiades F, Grimes H, Habeeb A, Hudson V, Hutchinson P*, Irune E, Jah A*, Khan DZ, Kolias A, Kyriacou H, Lamb B, Liau S*, Luke L, Mahmoud R, Mannion R, Masterson L*, Mitrofan C, Mohan M, Morris A, Murphy S, O’Neill JR*, Price S, Pushpa-rajah J, Raby-Smith W, Ramzi J, Rooney SM, Santarius T, Singh AA, Stewart GD*, Tan XS, Townson A, Tweedle E, Walker C, Waseem S, Yordanov S (Addenbrooke’s Hospital, Cambridge); Jones T, Kattakayam A, Loh C, Lunevicius R, Nunes Q, Pringle S, Schache A, Shaw R*, Sheel A, Sud A, Sundhu M (Aintree University Hospital, Liverpool); Rossborough C (Altnagelvin Area Hospital, London); Angelou D, Choynowski M, McAree B*, McCanny A, Neely D (Antrim Area Hospital- Northern Health And Social Care Trust, Antrim); Kamel F, Kumar L, Madani R*, Nisar P (Ashford And St Peter’s Hospital, Chertsey); Tutoveanu G* (Barnsley Hospital Nhs Foundation Trust, Barnsley); Ali S, Bittar MN*, Creanga M*, Elniel M, Law J, Youssef M* (Blackpool Victoria Hospital, Blackpool); Ahad S, De La Cruz Monroy MFI, Hashem M, Langlands F, Mosley F*, Oktseloglou V*, Omar I, Patel F* (Bradford Royal Infirmary, Bradford); Alanbuki A*, Patel M, Shabana A (Brighton And Sussex Nhs Trust, Princess Royal Hospital, Hayward’s Heath); Rathinaezhil R* (Brighton And Sussex University Hospitals Nhs Trust, Brighton); Perera E, Raveendran D, Ravi-Shankar K, Thiruchelvam J* (Broomfield Hospital, Chelmsford); Che Bakri NA, Jawad Z, Jiao L, Nazarian S, Vashisht R* (Bupa Cromwell Hospital, London); Arrowsmith L*, Campbell W* (Causeway Hospital, Coleraine); Grove T, Kontovounisios C, Warren O* (Chelsea And Westminster Hospital, London); Rolland P* (Cheltenham General Hospital, Cheltenham); Aggarwal A, Brown S, Jelley C, Neal N* (Churchill Hospital) Kaur R, Leung E*, Sundar S* (City Hospital, Dudley Road, Birmingham); Doulias T*, Li M, Martin E, Rodwell H (Colchester Hospital University, Colchester); Clifford R, Eardley N, Krishnan E, Manu N, Martin E, Roy Mahapatra S, Serevina OL, Smith C, Vimalachandran D* (Countess Of Chester Hospital, Chester); Bordenave M*, Houston R, Putnam G, Robson A*, Tustin H (Cumberland Infirmary, Carlisle); Emslie K*, Labib PL*, Marchbank A, Miller D, Minto G, Natale J, Nwinee H, Panahi P, Rogers L* (Derriford Hospital, Plymouth); Abubakar A*, Akhter Rahman MM, Chan E, O’Brien H, Sasapu K* (Diana Princess Of Wales Hospital Grimsby, Grimsby); Inglis R, Ng HJ* (Dumfries And Galloway Royal Infirmary, Dumfries); De Gea Rico A, Ghazali N*, Lambert J, Markose G, Math S, Sarantitis I, Shreshtha D, Simpson R*, Sonanis S, Sultana A*, Taggarsi M, Timbrell S, Vaz OP, Vitone L* (East Lancashire Hospitals Nhs Trust, Blackburn); Day A*, Dent H, Fahim M, Waheed S* (East Surrey Hospital, Redhill); Hunt A, Laskar N* (East Sussex Healthcare (Conquest Hospital And Eastbourne District General Hospital), Hastings); Gupta A*, Steinke J, Thrumurthy S (Epsom & St Helier University Hospitals Nhs Trust, Epsom); Massie E, McGivern K, Rutherford D, Wilson M* (Forth Valley Royal Hospital, Larbert); Hardie J, Kazzaz S* (Frimley Health Nhs Ft- Frimley Park, Camberley); Bacarese-Hamilton T, Ip M*, James A, Salerno G*, Stockdale T (Frimley Health Nhs Ft - Wexham Park, Slough); Handa S, Kaushal M, Kler A, Patel P*, Redfern J, Tezas S (Furness General Hospital, Barrow In Furness); Aawsaj Y, Amonkar S, Barry C, Blackwell L, Blake D, Carter J, Emerson H, Fisher A*, Katory M, Korompelis P, McCormick W, Mustafa A, Pearce L, Ratnavelu N*, Reehal R (Gateshead Health Nhs Foundation Trust, Gateshead); Damola A*, Kretzmer L*, Lalou L, Lim P, Manku B, Parwaiz I, Sandher M, Stafford J (George Eliot Hospital, Nuneaton); Abdelkarim M, Asqalan A, Gala T, Ibrahim S, Maw A*, Mithany R, Morgan R*, Sundaram Venkatesan G (Glan Clwyd Hospital, Rhyl); Holroyd D, Jamieson N*, Jones C, Shin JS (Glasgow Royal Infirmary, Glasgow); Ang K, Caruana EJ*, Chandarana K, Chowdhry MF, Mohammad A, Nakas A, Rathinam S (Glenfield Hospital, Leicester); Banfield D, Boal M*, Brown O, Dean H, Dwerryhouse S*, Higgs S, Vallance A (Gloucestershire Royal Hospital, Gloucester); Boyd E, Irvine V, Kirk A* (Golden Jubilee National Hospital, Glasgow); Bakolas G, Boulton A, Chandock A*, Khan T, Kumar M* (Good Hope Hospital, Sutton Coldfield); Agoston P, Billè A, Challacombe B*, Fraser S, Harrison-Phipps K, King J, McCrindle S, Mehra G, Mills L, Najdy M, Nath R, Okiror L, Pilling J, Rizzo V, Routledge T, Sayasneh A*, Stroman L*, Wali A (Guy’s Hospital, London); Fehervari M*, Fotopoulou C*, Habib N, Hamrang-Yousefi S, Jawad Z, Jiao L, Pai M, Ploski J, Rajagopal P, Saso S, Sodergren M, Spalding D (Hammersmith Hospital, London); Laws S* (Hampshire Hospitals Nhs Trust, Wincheter & Basingstoke); Hardie C, McNaught C* (Harrogate District Hospital, Harrogate); Alam R, Budacan A, Cahill J, Kalkat M*, Karandikar S*, Kenyon L, Naumann D, Patel A (Heartlands Hospital, Birmingham); Chen F, Cheung J* (Hinchingbrooke Hospital, Huntingdon); Ayorinde J, Chase T, Cuming T, Ghanbari A, Humphreys L, Tayeh S* (Homerton University Hospital, London); Aboelkassem Ibrahim A, Bichoo R, Cao H, Chai AKW, Choudhury J, Evans C, Fitzjohn H, Ikram H, Khalifa E, Langstroth M, Loubani M*, McMillan A, Nazir S, Qadri SSA, Robinson A, Ross E, Sehgal T, Wilkins A (Hull University Teaching Hospitals Nhs Trust, Hull); Dixon J*, Dunning J, Freystaetter K, Jha M, Kusuma VRM, Lester S*, Madhavan A, Thulasiraman SV, Viswanath Y* (James Cook University Hospital, Middlesbrough); Curl-Roper T, Delimpalta C, Liao CCL*, Velchuru V, Westwood E (James Paget Univeristy Nhs Foundation Trust Hospital, Great Yarmouth); Belcher E*, Bond-Smith G*, Chidambaram S, Di Chiara F, Fasanmade K, Fraser L, Fu H, Ganau M*, Gore S, Goricar M, Graystone J, Jeyaretna D, Khatkar H, Lami M, Maher M, Mastoridis S, McVeigh J, Mihai R, Myatt R, Piper R*, Prabhu S, Risk OBF, Selbong U, Shah K, Silva P, Smillie R, Soleymani majd H, Sravanam S, Stavroulias D, Tebala GD, Vatish M*, Verberne C, Wallwork K, Williams MA, Winter SC* (John Radcliffe Hospital, Oxford); Ahmed I, Djouani A, Eddy B, Folkard S, Hassan F, Kommu S, Papadopoulos G, Simoes A, Streeter E, Tait-Bailey J, Thomas M, Wang W, Yao M* (Kent And Canterbury Hospital, Canterbury); Anscomb N*, Baldwin-Smith R, Davies M, Grainger C, Haji A, Haq A, Nunoo-Mensah JW, Rizk M (King’s College Hospital, London); Bhatti MI, Boyd-Carson H, Elsey E, Gemmill E, Herrod P*, Jibreel M, Lenzi E, Saafan T, Sapre D, Sian T, Watson N (King’s Mill Hospital, Sutton-In-Ashfield); Athanasiou A*, Borg E, Bourke G, Bradshaw L, Brunelli A*, Burke J, Coe P*, Costigan F, Elkadi H, Ho M*, Johnstone J, Kanatas A, Kantola V, Kaufmann A, Laios A, Lam S, MacInnes E*, Munot S, Nahm C, Otify M, Pompili C, Raslan M, Salminen H, Smith I, Theophilou G*, Toogood G, Wade R*, Ward D, West C (Leeds Teaching Hospitals Trust, Leeds); Al-Harbawee A, Alharawee A, Annamalai S, Ashmore C, Boddy A, Hossain T*, Irvine E, Kassam K*, Kourdouli A (Leicester Royal Infirmary, Leicester); Chean CS, Dharamavaram S, Gvaramadze A, Jibril A, Kulkarni N, Pereira I, Prusty L, Shanthakunalan K, Srikumar B, Thekkinkattil D* (Lincoln County Hospital, Lincoln); Adegbola S, Menakaya C, Noel J (Lister Hospital, Stevenage); Harky A, Shackcloth M* (Liverpool Heart And Chest Hospital, Liverpool); Askari A, Chan C*, Cirocchi N, Kudchadkar S, Patel K, Sagar J*, Shaw S, Talwar R* (Luton And Dunstable University Hospital, Luton); Abdalla M, Edmondson R, Ismail O, Jones D, Newton K, Stylianides N* (Manchester Royal Infirmary, Manchester); Aderombi A, Andaleeb U, Bajomo O, Beatson K, Garrett W*, Mehmood M, Ng V (Medway Hospital, Gillingham); Al-Habsi R, Brimioulle M*, Divya GS, Keeler B*, Soulsby RE*, Taylor A (Milton Keynes University Hospital, Milton Keynes); Al-Sarireh B*, Clancy R, Cripps P, Dobbs T*, Egan R*, Fabre I, Harries R*, Henry A, Kittur M*, Li Z, Parkins K, Soliman F, Spencer N, Thompson D (Morriston Hospital Swansea, Swansea); Burgess C*, Gemmell C, Grieco C, Hollyman M*, Hunt L*, Morrison J*, Ojha S, (Musgrove Park Hospital, Taunton); Abbadessa F, Barnard S, Chan C, Dawe N, Hammond J, Koshy RM, Mahmoud Ali F, McPherson I, Mellor C, Moir J, Pandanaboyana S*, Powell J, Rai B*, Rogers A*, Roy C, Sachdeva A, Saleh C, Tingle S, Williams T (Newcastle Upon Tyne Hospitals Nhs Foundation Trust, Newcastle Upon Tyne); Manickavasagam J*, McDonald C*, McGrath N, McSorley N, Ragupathy K*, Ramsay L, Solth A (Ninewells Hospital, Dundee); Aristotelous C*, Kakisi O, Seebah K, Shaikh I*, Sreedharan L, Touil L, Youssef M* (Norfolk And Norwich University Hospital, Norwich); Shah J* (North Manchester General Hospital, Manchester); Ameerally P, Baguley M, Gnanachandran C*, Heer B, Rogers M, Woods R* (Northampton General Hospital, Northampton); Aujayeb A, Mills S* (Northumbria Nhs Hospital Trust, North Shields); Abu J, Addae-Boateng E, Bratt D, Brock L, Burnside N*, Cadwell-Sneath S, Gajjar K*, Gan C, Grundy C, Hallam K, Hassell K, Hawari M, Joshi A, Khout H, Konstantinidi K, Lee RXN, Nunns D, Schiemer R, Walton T*, Weaver H, Whisker L*, Williamson K (Nottingham City Hospital, Nottingham); McVeigh J, Myatt R*, Williams MA (Nuffield Orthopaedic Centre, Oxford); Ahmed ME, Bukhari SI, Illingworth B, Kanthasamy S, Knights E, Ong SL, Pujari R, Tan KHM, Vanker R* (Peterborough City Hospital, Peterborough); Michel M, Patil S, Ravindran S, Sarveswaran J*, Scott L (Pinderfields Hospital, Wakefield); Biliatis I*, Edmond M, King E* (Poole Hospital, Poole); Babawale O, Hodgson D, Ismail M, Khan J*, Lokman U, Phan YC* (Queen Alexandra Hospital, Portsmouth); Almond M*, Bhangu A*, Breik O, Cato LD, Chowdhury YA*, Desai A*, Ford S*, Griffiths E*, Idle M, Kamal M, Karia K, Kisiel A, Kulkarni R, Mak JKC, Martin T, Nankivell P*, Parente A, Parmar S, Pathanki AM, Phelan L, Praveen P, Saeed S, Sharma N, Singh J*, Solomou G, Soon WC, Stevens A, Tirotta F, Topham C, Ughratdar I, Vijayan D (Queen Elizabeth Hospital Birmingham, Birmingham); Ballantyne K*, Barker L, Chapman K, Charalambous M*, Chianakwalam C, English C, Evans J*, Fell A*, Frimpong D, Halkias C, Iyer R, Merh R, Neagu G, Nikolaou S, Poddar A, Pronisceva V, Reddy V, Williams N (Queen Elizabeth The Queen Mother Hospital Margate, Margate); Alakandy L, Bhattathiri P, Brown J, Canty M, Day E*, Geddes A, Grivas A, Hassan S, Lammy S*, Littlechild P, Maseland C, Mathieson C, McCaul J*, McMahon J, O’Kane R, St. George E, Suttner N, Taylor W, Tilling E (Queen Elizabeth University Hospital, Glasgow); English W, Kaul S, Khan AH, Khan F, Mansuri A, Mukherjee S*, Patel M, Sarigul M, Singh S, Smith C, Tan KL, Vulliamy P, Woodham A, Yang YH (Queen’s Hospital Romford, Romford); Adiamah A, Brewer H, Chowdhury A*, Evans J, Humes D*, Jackman J, Koh A, Lewis-Lloyd C, Navarro A*, Oyende O, Reilly J, Vohra R*, Worku D (Queens Medical Centre, Nottingham); Cool P, Cribb G, Shepherd K* (Robert Jones And Agnes Hunt Orthopaedic Hospital, Oswestry); Bisset C, Moug S* (Royal Alexandra Hospital, Paisley); Chadha R* (Royal Berkshire Hospital, Reading); Elson N, Galleano R, Faulkner G*, Langone A, Panayi Z, Saleh P, Tuminello F, Underwood C (Royal Bolton Hospital, Farnworth); Brixton G, Findlay L, Klatte T*, Majkowska A, Manson J*, Potter R (Royal Bournemouth Hospital, Bournemouth); Al-Khyatt W, Awad S*, Bhalla A*, Chia Z, Daliya P, Goyal A*, Grimley E, Hamad A, Kumar A*, Malcolm FL, Ng JCK, Phillips A, Theophilidou E, Williams S (Royal Derby Hospital, Derby); Bowden J, Campain N, Daniels I, Evans C, Fowler G, John J, Massey L, McDermott F*, McGrath J*, McLennan A, Ng M, Pascoe J, Rajaretnam N (Royal Devon And Exeter Hospital, Exeter); Angamuthu N, Bulathsinhala S, Chowdhury S, Davidson B, Fusai G, Gilliland J, Hart C, Hidalgo Salinas C, Knowles J, Machairas N, Mirnezami R, Pissanou T, Pollok JM*, Raptis DA, Soggiu F, Tzerbinis H, Varcada M*, Xyda S (Royal Free Hospital, Hampstead); Beamish A, Davies E, Foulkes R*, Magowan D, Nassa H, Ooi R, Price C, Smith L, Solari F, Tang A, Williams G* (Royal Gwent Hospital, Newport); Abd Kahar NN, Al-Tamimi Y, Bacon A, Beasley N, Catto J, Chan LH, Chew D, Crank M, Ilenkovan N, Macdonald M, Narice B, Rominiyi O*, Saad S, Sinha S, Thompson A, Varley I* (Royal Hallamshire Hospital, Sheffield); Brennan P, Drake T*, Harrison EM*, Linder G, Mayes J, McGregor R, Pasricha R, Skipworth RJE*, Zamvar V* (Royal Infirmary Of Edinburgh, Edinburgh); Davies E*, Hawkin P, Raymond T, Ryska O (Royal Lancaster Infirmary, Morecambe); Baron R*, Dunne D, Gahunia S, Halloran C, Howes N*, McKinney R, McNicol F*, Rajput K, Russ J, Sutton R, Szatmary P, Tan JR, Thomas A, Whelan P (Royal Liverpool University Hospital, Liverpool); Anzak A, Banerjee A, Fuwa O, Hughes F*, Jayasinghe JD, Knowles C, Kocher HM*, Leal Silva I, Ledesma FS, Minicozzi A*, Navaratne L, Patki P*, Rahman R, Ramamoorthy R, Sohrabi C, Tanabalan C, Thaha M*, Thakur B*, Venn M, Yip V* (Royal London Hospital, London); Baumber R, Parry J* (Royal National Orthopaedic Hospital, Stanmore); Evans S, Jeys L, Morris G, Parry M*, Stevenson J (Royal Orthopaedic Hospital, Birmingham); Ahmadi N, Aresu G, Barrett-Brown ZM, Coonar A*, Durio Yates H, Gearon D, Hogan J, King M, Peryt A, Pradeep IS, Smith C (Royal Papworth Hospital, Cambridge); Adishesh M*, Atherton R*, Baxter K, Brocklehurst M, Chaudhury M, Krishnamohan N*, McAleer J, Owens G, Parkin E*, Patkar P, Phang I* (Royal Preston Hospital, Preston); Aladeojebi A, Ali M, Ali S, Barmayehvar B, Gaunt A*, Gowda M, Halliday E, Kitchen M*, Mansour F, Nanjaiah P*, Thomas M*, Zakai D* (Royal Stoke University Hospital, Stoke-On-Trent); Abbassi-Ghadi N, Assalaarachchi H, Currie A, Flavin M, Frampton A*, Hague M, Hammer C, Hopper J, Horsnell J*, Humphries S, Kamocka A, Madhuri TK*, Preston S, Singh P*, Stebbing J, Tailor A, Walker D* (Royal Surrey County Hospital, Guildford); Coomber E, Jaunoo S, Kennedy L*, Williams O (Royal Sussex County Hospital, Brighton); Airey A, Bunni J, Crowley R, Fairhurst K*, Frost J*, George R, Lee S, Mitchell S, Phull J, Richards S (Royal United Hospital Bath, Bath); Aljanadi F, Campbell A, Glass A, Hraishawi I, Jones M*, McIlmunn C, McIntosh S*, Mhandu P, O’Donnell C, Turkington R* (Royal Victoria Hospital, Belfast); Al- Ishaq Z, Bhasin S, Bodla AS, Burahee A, Crichton A, El-Ghobashy A*, Fossett R, Pigadas N*, Rahman E, Snee D, Vidya R, Yassin N* (Royal Wolverhampton Nhs Trust, Wolverhampton); Colombo F, Fountain D, Hasan MT, Karabatsou K*, Laurente R, Pathmanaban O* (Salford Royal Hospital, Salford); Barlow C, Ding D, Foster J, Longstaff L (Salisbury Nhs Foundation Trust, Salisbury); Brett-Miller C, Buruiana FE, Kaur R, Leung E*, Sundar S* (Sandwell General Hospital, Birmingham); Al-mukhtar A, Brown S*, Edwards J*, Giblin A, Kelty C, Lee M, Lye G, Newman T, Sharkey A, Steele C, Sureshkumar Shah N, Whitehall E (Sheffield Teaching Hospital Nhs Foundation Trust, Sheffield); Blair J, Lakhiani A, Parry-Smith W, Sahu B* (Shrewsbury And Telford Hospitals, Shrewsbury); Athwal R*, Baker A, Jones L, Konstantinou C, Ramcharan S*, Singh S, Vatish J, Wilkin R (South Warwickshire Nhs Foundation Trust, Warwick); Alzetani A*, Amer K, Badran A*, Colvin HV, Ethunandan M, Sekhon GK, Shakoor Z, Shields H, Singh R*, Talbot T, Wensley F (Southampton General Hospital, Southampton); Lawday S, Lyons A* (Southmead Hospital, Bristol); Newman S (Southport And Formby District General Hospital, Southport); Chung E, Hagger R, Hainsworth A, Hunt I, Karim A, Owen H, Ramwell A, Santhirakumaran G, Smelt J, Tan C, Vaughan P, Williams K* (St George’s Hospital, London); Baker C, Davies A, Gossage J, Kelly M*, Knight W (St Thomas’ Hospital, London); Bromage S, Hall J, Kaushik V, Rudic M*, Vallabh N, Zhang Y (Stepping Hill Hospital, Stockport); Harris G, James G, Kang C, Lin DJ, Rajgor AD, Royle T*, Scurrah R, Steel B, Watson LJ (Sunderland Royal Hospital, Sunderland); Kocher HM* (The London Clinic, London); Choi D, Hutchison R, Jain A, Luoma V, Marcus HJ*, May R, Menon A, Pramodana B, Webber L (The National Hospital For Neurology And Neurosurgery, London); Hayes A, Jones R, Sivarajah G, Smith M, Smrke A, Strauss D* (The Royal Marsden Nhs Foundation Trust, London); Abouelela FAM, Aneke IA, Asaad P, Brown B, Collis J, Duff S*, Khan A, Moura F, Taylor M, Wadham B, Warburton H (The University Hospital Of South Manchester, Manchester); Elmoslemany T, Jenkinson MD*, Millward CP, Zakaria R (The Walton Centre Nhs Foundation Trust, Liverpool); Mccluney S, Parmar C*, Shah S (The Whittington Hospital, London); Allison J, Babar MS*, Bowen J, Collard B, Goodrum S, Lau K, Patel A, Sargent M, Scott R*, Thomas E, Whitmore H (Torbay And South Devon Nhs Trust, Torquay); Balasubramaniam D*, Jayasankar B*, Kapoor S, Ramachandran A (Tunbridge Wells Hospital, Maidstone); Semple C* (Ulster Hospital Dundonald, Dundonald); Elhamshary A, Imam SMB, Kapriniotis K, Kasivisvanathan V*, Lindsay J, Rakhshani- Moghadam S (University College London Hospital At Westmoreland Street, London); Beech N, Chand M*, Green L, Kalavrezos N*, Kiconco H, McEwen R, Schilling C, Sinha D (University College London Hospital, London); Pereca J*, Singh J (University Hospital Ayr, Ayr); Chopra S, Egbeare D*, Thomas R (University Hospital Llandough, Cardiff); Arumugam S, Ibrahim B*, Khan K* (University Hospital North Durham, Durham); Combellack T*, Hill G*, Jones S*, Kornaszewska M, Mohammed M, Tahhan G, Valtzoglou V, Williams J (University Hospital Of Wales, Cardiff); Blencowe N, Eskander P, Gash K, Gourbault L, Hanna M, Maccabe TA, Main B, Olivier J*, Newton C*, Roswadowski S, Ryan N, Teh E, West D*(University Hospitals Bristol Nhs Foundation Trust, Bristol and Weston NHS Foundation Trust); Al-omishy H, Baig M, Bates H, Di Taranto G, Dickson K, Dunne N, Gill C, Howe D*, Jeevan D, Khajuria A, Martin-Ucar A*, McEvoy K, Naredla P, Ng V, Robertson S*, Sait M, Sarma DR, Shanbhag S*, Shortland T, Simmonds S, Skillman J, Tewari N*, Walton G (University Hospitals Coventry And Warwickshire Nhs Trusts, Coventry); Akhtar MA*, Brunt A, McIntyre J, Milne K, Rashid MM, Sgrò A, Stewart KE, Turnbull A (Victoria Hospital Kirkcaldy, Kirkcaldy); Abou-Foul AK* (Walsall Manor Hospital, Walsall); Gossedge G*, O’Donnell S, Oldfield F (Warrington & Halton Teaching Hospitals Nhs Trust, Warrington); Thomson S* (West Hertfordshire Hospitals Nhs Trust, Hertfordshire); Aguilar Gonzalez M*, Talukder S* (West Suffolk Hospital, Bury St Edmunds); Boyle C, Fernando D, Gallagher K, Laird A*, Tham D (Western General Hospital, Edinburgh); Bath M, Patki P*, Sohrabi C, Tanabalan C (Whipps Cross University Hospital, London); Basnyat P*, Davis H, Montauban P, Shrestha A (William Harvey Hospital, Ashford); Agarwal K*, Arif T, Magee C*, Nambirajan T*, Powell S*, Vinayagam R* (Wirral University Teaching Hospital, Wirral); Flindall I, Hanson A, Mahendran V (Worcestershire Royal Hospital, Worcester); Green S, Lim M, MacDonald L, Miu V, Onos L, Sheridan K, Young R* (York Teaching Hospitals Nhs Trust, York); Alam F, Griffiths O, Houlden C, Jones R*, Kolli VS, Lala AK, Leeson S, Peevor R, Seymour Z* (Ysbyty Gwynedd, Bangor, North Wales).

United States of America: Consorti E, Gonzalez R, Grolman R, Kwan-Feinberg R*, Liu T, Merzlikin O (Alta Bates Summit Medical Center (Sutter Health), San Francisco); Brown A, Cooper Z*, Hirji S, Jolissaint J, Mahvi D, Okafor B, Raut CP*, Roxo V, Salim A (Brigham And Women’s Hospital, Boston); Bessen S, Chen L, Dagrosa L, Fay K, Fleischer C, Hasson R, Henderson E, Leech M, Loehrer A*, Markey C, Paydarfar J, Rosenkranz K, Telma K, Tocci N, Wilkinson-Ryan I, Wilson M (Dartmouth-Hitchcock Medical Center, Lebanon, Nh); Bokenkamp M, Brown K*, Fleming D*, Haynes A*, Heron C, Hill C, Kay H, Leede E, McElhinney K, Olson KA, Osterberg EC*, Riley C, Srikanth P (Dell Seton Medical Center At The University Of Texas, Austin); Barbour J, Blazer D*, DiLalla GA*, Fayanju O, Hwang ES*, Kahmke R, Kazaure H, Lazarides A, Lee W*, Lidsky M*, Menendez C, Moris D, Plichta J, Pradhan MC, Puscas L, Rice HE, Rocke D, Rosenberger L*, Scheri R, Smith BD, Stang MT, Tolnitch L, Turnage K, Visgauss J, Walton FS, Watts T, Zani S (Duke University Medical Center, Durham, Ma); Farma J* (Fox Chase Cancer Center, Philadelphia); Cardona K, Russell MC (Emory University, Atlanta, GA); Clark J, Kwon D* (Henry Ford Hospital, Detroit, Michigan); Goel N*, Kronenfeld J (Jackson Memorial Hospital, Miami); Bigelow B, Etchill E*, Gabre-Kidan A*, Jenny H, Kent A, Ladd MR, Long C, Malapati H, Margalit A, Rapaport S, Rose J, Stevens K, Tsai L, Vervoort D, Yesantharao P (Johns Hopkins Hospital, Baltimore, Md); Dehal A* (Kaiser Permanente Panorama City Medical Center, Panorama City); Klaristenfeld D* (Kaiser Permanente San Diego Medical Center, San Diego); Huynh K (Kaiser Permanente West Los Angeles, Los Angeles); Kaafarani H*, Naar L, Qadan M (Massachusetts General Hospital, Boston, Ma); Brown L, Ganly I* (Memorial Sloan Kettering Cancer Center, New York); Mullinax JE* (Moffitt Cancer Center, Tampa); Alpert N, Gillezeau C, Miles DDS MD FACS BA*, Taioli E (Mount Sinai Hospital, New York); Cha DE, Gleeson E, Horn C, Sarpel U* (Mount Sinai, New York); Gusani N, Hazelton J*, Maines J, Oh JS, Ssentongo A, Ssentongo P (Pennsylvania State University, Hershey); Bhama A (Rush University Medical Centre, Chicago, Il); Colling K*, Najarian M (Saint Mary’s Medical Center-Essentia Health, Duluth); Azam M, Choudhry A*, Marx W (Suny Upstate University Hospital, Syracuse); Abedin Y, Arzumanov G, Chokshi R, Gabrilovich S, Glass N*, Kalyoussef E, Parvin- Nejad FP, Roden D, Stein J, Suarez-Ligon A, Tsui G, Zhao K (The University Hospital, Newark, Nj); Fleming J, Fuson A, Gigliotti J, Ovaitt A, Ying Y* (University Of Alabama Birmingham, Birmingham); Abel MK, Andaya V, Bigay K, Boeck MA, Chen L, Chern H, Corvera C, El-Sayed I, Glencer A, Ha P, Hamilton BCS, Heaton C, Hirose K, Jablons DM, Kirkwood KS, Kornblith LZ*, Kratz JR, Lee RH, Miller PN, Nakakura EK, Nunez-Garcia B, O’Donnell RJ, Ozgediz D, Park P, Robinson B, Sarin A, Sheu B, Varma MG, Wai KC, Wustrack R, Xu MJ, Zimel M (University of California San Francisco (UCSF), San Francisco, CA) Beswick D*, Goddard J, Manor J, Song J (University Of Colorado Hospital/Memorial Hospital/Medical Center Of The Rockies (All Within Uchealth System), Denver/Colorado Springs/Loveland); Cioci A, Pavlis W, Rakoczy K, Ruiz G, Saberi R (University Of Miami Hospital, Miami, Fl); Fullmer T, Gaskill C, Gross N*, Kiong K, Roland CL*, Zafar SN (University Of Texas Md Anderson Cancer Center, Houston); Abdallah M, Abouassi A, Aigbivbalu E, Almasri M, Eid J, George B, Kulkarni G, Marwan H*, Mehdi M, San Andrés M, Sundaresan J (University Of Texas Medical Branch, Galveston); Aoun SG, Ban VS*, Batjer HH, Bosler K, Caruso J, Sumer B* (University Of Texas Southwestern, Dallas); Abbott D, Acher A, Aiken T, Barrett J, Foley E, Schwartz PB, Zafar SN* (University Of Wisconsin, Madison); Hawkins AT*, Maiga A (Vanderbilt University Medical Center, Nashville, TN); Ruzgar NM, Sion M, Ullrich S (Yale New Haven Hospital, New Haven, Ct). Uruguay: Laufer J*, Scasso S* (Hospital Pereira Rossell, Montevideo, Montevideo).

Yemen: Al-Naggar H*, Al-Shehari M*, Almassaudi A, Alsayadi M, Alsayadi R, Nahshal M, Shream S (Al- Thawra Modern General Hospital, Sana’a); AL-Ameri S, Aldawbali M (Royal Hospital, Sana’a).

### GlobalSurg-3

#### National Leads

Albania: Arben Gjata; Argentina: Maria Marta Modolo; Australia: Sebastian King, Erick Chan; Bangladesh: Sayeda Nazmun Nahar; Barbados: Ade Waterman; Belgium: Dominique Vervoort; Benin: Ismaïl Lawani; Botswana: Alemayehu Ginbo Bedada; Brazil: Bernardo De Azevedo, Ana Gabriela Figueiredo; Bulgaria: Manol Sokolov; Burundi: Venerand Barendegere; Cameroon: Gerald Ekwen; Canada: Arnav Agarwal, Anna Dare; China: Qinyang Liu; Colombia: Juan Camilo Correa; Congo, Dem. Rep.: Kalisya Luc Malemo, Jacques Bake; Croatia: Jakov Mihanovic; Czech Republic: Kamila Kunčarová, Julius Orhalmi; Egypt: Hosni Salem; Estonia: Jyri Teras; Finland: Aristotelis Kechagias; France: Alexis P Arnaud; Germany: Judith Lindert; Ghana: Stephen Tabiri; Greece: Vasileios Kalles; Guatemala: Maria-Lorena Aguilera-Arevalo, Gustavo Recinos; Hungary: Zsolt Baranyai; India: Basant Kumar, Harish Neelamraju Lakshmi, Sanoop Koshy Zachariah, Philip Alexander, Sunil Kumar Venkatappa, C Pramesh; Indonesia: Radhian Amandito; Ireland: Christina Fleming; Italy: Luca Ansaloni, Francesco Pata, Gianluca Pellino; Jordan: Ahmed M. Altibi, Ibrahim Nour; Kenya: Intisar Hamdun; Libya: Muhammed Elhadi, Ali M. Ghellai; Lithuania: Donatas Venskutonis, Tomas Poskus, Justas Zilinskas; Madagascar: John Whitaker; Malawi: Precious Malemia; Malaysia: Yong Yong Tew; Malta: Elaine Borg, Sarah Ellul; Mexico: Antonio Ramos-De la Medina; Morocco: Fatima Zahraa Wafqui; Namibia: David W Borowski; Netherlands: Anne Sophie van Dalen; New Zealand: Cameron Wells; Niger: Harissou Adamou; Nigeria: Adesoji Ademuyiwa, Adewale Adisa; Norway: Kjetil Søreide; Pakistan: Ahmad Uzair Qureshi; Palestine: Ibrahim Al-Slaibi, Sara Al Saqqa, Osaid Alser, Haya Tahboub; Paraguay: Helmut Alfredo Segovia Lohse; Peru: Sebastian Shu Yip; Philippines: Marie Carmela Lapitan; Poland: Piotr Major; Portugal: Joana Simões, António Sampaio Soares; Romania: Matei Razvan Bratu; Russian Federation: Andrey Litvin, Armen Vardanyan; Rwanda: JC Allen Ingabire, Ainhoa Costas-Chavarri; Saudi Arabia: Ahmad Gudal, Naif Albati; Serbia: Jovan Juloski; Singapore: Bettina Lieske; Slovenia: Miran Rems; South Africa: Sarah Rayne, Stephanie Van Straten, Yoshan Moodley, Kathryn Chu, Rachel Moore; Spain: Irene Ortega Vázquez, Jaime Ruiz-Tovar; Sri Lanka: Kithsiri Janakantha Senanayake, Sujeewa Priyantha Bandara Thalgaspitiya; Sudan: Omer Abdelbagi Omer, Anmar Homeida; Sweden: Yucel Cengiz; Switzerland: Daniel Clerc; Syrian Arab Republic: Muhammad Alshaar; Tunisia: Hanen Bouaziz; Turkey: Yuksel Altinel; Uganda: Matthew Doe; Ukraine: Maryna Freigofer;

United Kingdom: Ella Teasdale, Rakan Kabariti, Joshua Michael Clements, Stephen Richard Knight, Ahsan Ashfaq; United States: Ijeoma Azodo; Uruguay: Gabriela Wagner, Ivan Trostchansky; Zambia: Mayaba Maimbo, David Linyama.

#### *Local collaborators* (*denotes hospital lead(s))

Albania: Helidon Nina, Amanda Zeko (University Hospital Center Nene Tereza).

Argentina: Claudio Gabriel Fermani, Maria Marta Modolo, Santiago Villalobos (Hospital Luis Lagomaggiore); Federico Carballo, Pablo Farina, Sebastian Guckenheimer (Ignacio Pirovano).

Australia: Marilla Dickfos (Bundaberg Base Hospital); Ankit Ajmera, Chester Chong, Ralph Gourlay, Sikandar Hussaini, Yi Jia Lee, Adeeb Majid, Peter Martin, Rebecca Miles, Owen James Morris, Jamie Phua, William Ridley, Tarunpreet Saluja, Ryan Renxin Tan, Jen Teh, Anna Wells (Calvary Mater Newcastle); Bharti Arora, Qaasim Dollie, Debbie Ho*, Yanru Ma, Omattage Mahasha Perera, Anthony Truong (Gold Coast University Hospital); Amanda Caroline Dawson*, Bryan Lim, Upuli Pahalawatta, Jacqueline Phan, Xiao-Ming Sarah Woon-Shoo-Tong, Andrea Yeoh (Gosford Hospital); Lillian Charman, Andrew Drane, Sharon Laura, Charmaine Chu Wen Lo, Amy Mozes, Rita Poon*, Hao Han Tan, Ellen Wall (Gosford Private Hospital); Prakshi Chopra, Jasmine De Giovanni, Bal Dhital, Brian Draganic, Alexander Duller, Jonathan Gani, Yao Kuan Goh, Jun Young Jeong, Brendan McManus, Prakash Nagappan, Peter Pockney, Anya Rugendyke, Mahsa Sarrami, Stephen Smith, Vanessa Wills, Hsu Ven Wong, Geoffrey Ye, Geoffrey Zhang (John Hunter Hospital); Ethan Brooker, Daniel Feng, Bonnie Lau, Carlin Ngai (Manning Base Hospital); Sarah Birks, David Gyorki, Jaime Otero de Pablos (Peter MacCallum Cancer Centre); Ali Abbosh, Chris Gillespie, Ahmed Mahmoud* (Princess Alexandra Hospital); Bianca Kwan, Joshua Lawson, Andrea Warwick (Redcliffe Hospital); Janne Bingham, Andrew J Cockbain, Nagendra Naidu Dudi-Venkata, Jordan Ellaby-Hall, Ben Finlay, Emily Humphries, Jade Pisaniello, Monique Pisaniello, Salma Salih, Tarik Sammour* (Royal Adelaide Hospital); Haidar Hadri Abd Wahab, April De Silva, Nicola Hayward, Kartik Iyer, Guy Maddern*, Gian Andrea Prevost (The Queen Elizabeth Hospital); Naga Annapureddy, Krishna Pranathi Settipalli, Jeremy Yeo (The Wesley Hospital); Lucy Hempenstall, Lily Pham, Shaun Purcell (Toowoomba Hospital); Cherry Talavera, Ashish I Vaska (University Hospital Geelong); Gurpreet Chaggar, Phillip Chrapko, Annelise Cocco, Sarah Michelle Crystal Jade Coulter-Nile, Grahame Ctercteko, James French, Houchen Gong, Martijn Gosselink, Thuvarahan Jegathees, Ivan Jin, Michelle Kalachov, Kathryn Kiefhaber, Katherine Lee, Jason Luong, Steven Phan, Henry Pleass, Kelly Veale, Zhi Zeng (Westmead Hospital); Angela Au, Ashe DeBiasio, Idy Deng, Jananee Myooran, Amrita Nair, Peter Stewart* (Wyong Public Hospital).

Austria: Anton Stift, Lukas Walter Unger, Kerstin Wimmer (General Hospital of Vienna).

Bangladesh: Nabila Ahmed, Syed Hasan, Saber Rahman (Bangladesh Medical College Hospital).

Barbados: Margaret O’Shea, Greg Padmore, Adrian Peters (Queen Elizabeth Hospital).

Belgium: Pietro Perduca, Guenda Pulcina, Nicolas Tinton (Grand Hopital de Charleroi - Site Saint-Joseph); Frederic Buxant, Elsa Dabin, Giulia Garofalo (Hôpitaux Iris Sud - Etterbeek-Ixelles).

Benin: Francis Dossou, Ismaïl Lawani (Centre Hospitalier Universitaire et Departemental Oueme Plateau); Freddy Houehanou Rodrigue Gnangnon, Yacoubou Imorou Souaibou (Centre National Hospitalier et Universitaire Hubert Koutoukou Maga).

Botswana: Alemayehu Ginbo Bedada*, Pako Motlaleselelo, Omphile Tlhomelang (Princess Marina Hospital).

Brazil: Igor Lima Buarque, Gustavo Mendonça Ataíde Gomes, Aldo Vieira Barros (Hospital Santa Casa de Misericordia de Maceio).

Bulgaria: Ilia Batashki, Nikolai Damianov, Vladislav Stoyanov (Medical Institute of Ministry of Interior); Dragomir Dardanov, Svilen Maslyankov, Plamen Petkov, Manol Sokolov, George Todorov, Evgeni Zhivkov (University Hospital Alexandrovska); Aygulya Akisheva, Miguel Angel Castilla Moreno, Geno Genov, Ivelina Ilieva, Tsvetomir Ivanov, Martin Karamanliev, Azhar Khan, Emil Mitkov, Tsanko Yotsov (University Hospital Dr Georgi Stranski); Boyko Atanasov, Nikolay Belev, Mihail Slavchev (University Hospital Eurohospital).

Burundi: Carlos Nsengiyumva (Kamenge Military Hospital).

Cambodia: Elgan Jones, Simon Stock (World Mate Emergency Hospital).

Cameroon: Gerald Ekwen, Steve Kyota (Baptist Hospital); James Brown, Tresor Mabanza K., Lemery Nigo Samuel, Chidi Otuneme, Ngwang Prosper, Franklin Umenze (Mbingo Baptist Hospital).

Canada: Marylise Boutros, Natasha Caminsky, Sinziana Dumitra, Richard Garfinkle, Dominique Morency, Ebram Salama (Jewish General Hospital); Alexander Banks, Lorenzo Ferri, Haitian He, Amit Katz, Alexander Sender Liberman, Sarkis Meterissian, Allison Pang, Elena Parvez (McGill University Health Center); Arnav Agarwal, Anna Dare, Usmaan Hameed, Fahima Osman, Sangita Sequeira (North York General Hospital); Natalie Coburn, Anna Dare, Alisha Jaffer, Paul Karanicolas (Sunnybrook Hospital); Matthew Mosseler, Reilly Musselman (The Ottawa Hospital).

China: Xinyuan Liu, Ching Wan Yip (Huashan Hospital affiliated to Fudan University).

Colombia: Juan Sebastian Garces-Otero, Carolina Guzman, Sebastian Sierra, Andres Uribe Valencia (CES Clinic); Paulo Andrés Cabrera Rivera, Saul Camelo, Andrea Gonzalez, Alejandro González-Orozco, Manuel Santiago Mosquera Paz, Carlos J- Perez Rivera (Fundacion Cardioinfantil-IC); Felipe Gonzalez, Andres Isaza-Restrepo, Laura Nino- Torres (Hospital Universitario Mayor Méderi); Natalia Arias Madrid, Maria Clara Mendoza Arango, Sebastian Sierra (Hospital Universitario San Vicente Fundacion).

Congo, Dem. Rep.: Jacques Bake, Justin Tsandiraki (HEAL Africa Hospital).

Croatia: Damir Jemendžić, Branislav Kocman, Oliver Šuman (Clinical Hospital Merkur); Renata Canic, Darko Jurišić, Ivana Karakas, Ana Krizanovic Rupcic, Vlatka Pitlovic, Josip Samardžić (General Hospital Dr. Josip Bencevic); Mario Kopljar (University Hospital Center Sestre milosrdnice); Ivan Bacic, Edgar Domini, Robert Karlo, Jakov Mihanovic*, Danijela Miljanić, Andrea Simic (Zadar General Hospital).

Czech Republic: Mariam Ahmed, Majdi Al Nassrallah, Rabiya Altaf, Talal Amjad*, Ruba Eltoum, Heba Haidar, Alhassan Hassan, Omar Khalil, Marwan Qasem, Rommel Ramesh, Gautham Sajith, Maham Wisal (Charles University Hospital); Jan Žatecký* (Slezská nemocnice v Opavě, p.o.); Michele Bujda, Katerina Jirankova, Ales Paclik (The General University Hospital in Prague).

Egypt: Aya Abdallah, Mariam Abdulgawad Almogy, Esraa Ayman El-sawy, Ahmed Moustafa ElFayoumy, Nourhan Elghareeb, Nourhan Ahmed Esmat, Ahmed Fadel, Abdullah Habater, Heba Hamdy, Amr Hefni, Marwa Kamal, Norhan Mohamed Abobakr, Ahmed Sayed, Nancy Shaker, Ehab Taha, Hoda Tharwat, Omar Zakaria (Ain Shams University Specialized Hospital); Ibrahem Abdelmotaleb, Ali Al-Dhufri, Hamza S. Al-Himyari, Enas El sheikh, Asmaa Eldmaty, Aya Elkhalawy, Ahmed M.Elkhashen, Kithara Magdy, Safa Mostafa, Habib Doutoum Sadia, Mohamed mahmoud Saleh, Dina Samir, Mohamed Yahia Mohamed Ali (Al-Tagamoh Hospital); Mahmoud A. Nassar, Samar Abdelhady, Aly Abdelrazek, Israa Abdelsalam, Aya El-Sawy, Eman Essam, Mohamed Gadelkarim, Khaled Ghaly, Mohamed Hassabalnaby, Rana Masarani, Nourhan Mohamed Shaaban, Ahmed Sabry, Menatalla Salem, Nourhan Akram Soliman, Diaaaldin Zahran (Alexandria Main University Hospital); Moustafa Ramadan Abou El.soud, Esraa Tarek Badr, Hala Borham, Nehal Elmeslemany, Mohammad Elsayed, Fawzia Elsherif, Sara Eslam, Gehad Gaber, Sondos Ibrahim, Yara Kamh, Abdelrahman Mahmoud, Shimaa gamal Mohamed, Eman Morshedy, Cinderella Omar, Fatima Salem Soliman (Alexandria Medical Research Institute); Shaza Abdelkawy, Naglaa Abdelmohsen, Mahmoud Abdelshakour, Ahmed Dahy, Norhan Gamal, Mohammed Gamal, Ahmad Hasan, Helal Hetta, Nehad Mousa, Mohamed Omar, Somia Rabie, Mahmoud Saad, Bakeer Saleh, Marwa Sayed Mohamed, Muhammad Shawqi (Assiut University Hospital); Heba Abdelhady Mousa, Mostafa Alnoury, Mohamed Elbealawy, Ahmed Elshafey, Muhammad Essam Ibrahim El Desouki Muhammad Ahmed, Mennatullah Ghonaim, Fawzy Hgag, Mohamed Ibrahim, Mahmoud Morsy, Mohamed Reda Loaloa, Ahmed Refaat, Hadeer Samir, Fatma Shahien, Mohamed Sobhy, Fathy Sroor (Banha University Hospital); Esraa Abdellatif, Marina Adel, Amr Abdelghani Afifi, Eman Afifi, Marco Antaky, Amr Dawoud, Naira El Zoghby, Amira El-remaily, Ali Abdelazez Elzanfaly, Ahmed Gadallah, Fatma Alzahraa Gamal, Omar Hashem, Shrouk Medhat Youssef, Aliaa Muhammad Attyah, Malak Munir, Omar Shazly, Esraa Taha, Karim Wilson (El Demerdash University Hospital); Sawsan Adel, Asmaa Ali, Esraa Eid, Esraa Elhelow, Marwa Elmahdy, Bassant Elshatby, Amany Hossam el-din Zakaria, Ahmad Hossny, Eman Ibrahim, Ahmed M.Yonis, Maram Metwalli, Basant Yousry, Esraa Zid (Gamal Abd El Nasser Hospital); Mina A Yacoub, Ahmed Abdelhakim, Nervana Abouelsoad, Mo’min Alkhatib, Ahmed Ashraf, Alaa Ashraf, Yasmin Elazab, Mahmoud Elfanty, Osama Elkabir, Mai Elsayed, Ahmed Elshimy, Hager Elsobky, John Eskander, Ahmed Gad, Ward Hamsho, Noura Khaled Abdelwahed, Menna Magdy, Dalia Moharam, Abeer Osama, Shereen Ramadan, Radwa Roum, Taqwa Sayed, Tarneem Shehada, Ahmed Mohy Zidan (Kasr Alainy Hospital, Faculty of Medicine, Cairo University); Khalid Abbas, Amr Ali, Mohamed Attia, Mohamed Balata, Ayman El Nakeeb, Mohamed Ibrahim Elsayed Elewaily, Ahmed Elfallal, Hossam Elfeki, Ahmed Elkhadragy, Sameh Emile, Helmy Ezzat, Hasnaa Hosni, Islam Mansour, Waleed Omar, Gehad Othman, Kareem Sadek, Mostafa Shalaby, Noura Shehab-Eldeen (Mansoura University Hospital); Rawda Anas khalifa, Helmy Badr, Mostafa Eldeep, Ahmed Eldeep, Amany Eldoseuky mohammed, Salwa Khallaf, Eman Magdy Hegazy, Rokia Mahmoud, Pola Mikhail, Mahmoud Morsi, Sara Mowafy, Dina Raafat, Amina Safy, Marwa Sera, Ahmed shible Sera (Menofiya University Hospital); Mostafa Salim Mohamed AbdAllah, Muhammad Abdelkader, Abdulrahman Osama Abdou, Ahmedgaber Ahmed, Shireen Gaafar, Fatma Ibrahim negm, Mina Lapic, Ahmed Maher, Hagar Mahmoud, Ahmed Mostafa, Mohamed Samir, Fatma Samy, Nourhan Semeda, Hind I. Shalaby (National Cancer Institute); Alaa El-taweel, Ahmed Galal Elnagar, Ahmed Gamal Hemidan, Mohamed Hussein, Ahmed.A. Kandil, Mf Moawad, Ayat Allah Nasser Hamamah, Mostafa Soliman (National Institute of Diabetes and Endocrinology Hospital); Mohamed Abdelkhalek, Noura Abdelmaksoud Tawakel, Ahmed Mohamed Abdelwahed, Alrawy Abdou, Khalid Atallah, Mohammed Yasser Elsherbeny, Eman Emara, Mohamed Hamdy, Omar Hamdy, Amira Haron, Salma Ismail, Islam Hany Metwally, Nihal Mohamed Hamed Elgaml, Ahmed Nassar, Basel Refky, Mirna Sadek, Mahmoud Saleh, Asmaa Yunes, Mai Zakaria, Mohammed Zuhdy (Oncology Center Mansoura University); Notila Fayed, Mohammed Mustafa Hassan Mohammed (Zagazig University Hospitals).

Estonia: Sander Kütner, Priit Melnik, Indrek Seire, Jyri Teras, Toomas Ümarik (The North Estonia Medical Centre).

Finland: Eppu Ainoa, Verner Eerola, Hanna Koppatz, Laura Koskenvuo, Ville Sallinen, Sini Takala (Helsinki University Hospital); Jevgeni Katunin, Aristotelis Kechagias, Arto Turunen (Kanta-Hame Central Hospital).

France: Niki Christou, Muriel Mathonnet (CHU Limoges); Vincent Lavoue, Krystel Nyangoh Timoh, Lucie Soulabaille (CHU Rennes - Breast Surgery); Romain Lesourd, Aude Merdrignac, Laurent Sulpice (CHU Rennes - General Surgery); Benoît André, Elodie Chantalat, Charlotte Vaysse (CHU Toulouse); Bertrand Dousset, Sebastien Gaujoux, Gregory Martin (Hôpital Cochin - APHP).

Germany: Octavian Clonda, Domantas Juodis, Klaus Kienle, Andras Mravik, Samuel Palmer, Gabor Szabadhegyi (Rottal-Inn- Kliniken).

Ghana: Anita Eseenam Agbeko, Solomon Gyabaah, Frank Enoch Gyamfi, Nuhu Naabo, Atta Owusu senior, Joseph Yorke (Komfo- Anokye Teaching Hospital); Frank Owusu (St. Patrick’s Hospital); Francis Abantanga, Theophilus Teddy Kojo Anyomih, Abdul-Jalilu Mohammed Muntaka, Emmanuel Owusu Abem, Mohammed Sheriff, Stephen Tabiri, Paul M. Wondoh (Tamale Teaching Hospital).

Greece: Dimitrios Balalis*, Dimitrios Korkolis (Agios Savvas Anticancer Hospital); Georgios Gkiokas, Eirini Pantiora*, Theodosios Theodosopoulos (Aretaieion Hospital); Argyrios Ioannidis*, Konstantinos Konstantinidis, Sofia Konstantinidou (Athens Medical Center); Nikolaos Machairas, Anna Paspala, Anastasia Prodromidou* (Attikon University General Hospital); Christos Chouliaras, Konstantinos Papadopoulos* (General Hospital of Nikaia); Ioannis Baloyiannis, Ioannis Mamaloudis, George Tzovaras* (General University Hospital of Larissa); Ioanna Akrida, Maria-Ioanna Argentou, Stylianos Germanos, Evangelos Iliopoulos, Ioannis Maroulis, George Skroubis, George Theofanis (General University Hospital of Patras); Christos Chatzakis, Orestis Ioannidis*, Lydia Loutzidou (George Papanikolaou General Hospital of Thessaloniki); Vasileios Kalles, Panagiotis Karathanasis, Nikolaos Michalopoulos, Charalampos Theodoropoulos, Dimitrios Theodorou, Tania Triantafyllou* (Hippocratio General Hospital); Zoe Garoufalia, Natasha Hasemaki, Michalis Kontos, Gregory Kouraklis, Stylianos Kykalos, Theodore Liakakos, Eustratia Mpaili, Alexandros Papalampros, Dimitrios Schizas, Athanasios Syllaios, Ekaterini Christina Tampaki*, Antonios Tsimpoukelis (Laiko University Hospital); Maria Ioanna Antonopoulou, Eirini Deskou, Dimitrios K. Manatakis*, Dimitrios Papageorgiou, Menelaos Zoulamoglou (Naval And Veterans Hospital); Christos Anthoulakis, Michalis Margaritis, Nikolaos Nikoloudis* (Serres General Hospital).

Guatemala: Veronica Campo, André Ceballos, Mario-Andrés Flores, Waleska Giron, Donghyun Ko, Gabriel Martinez, Gustavo Recinos*, Verónica Rivera Lara, Nataly Rueda, Andres Sanchez, Jorge Carlos Guillermo Tejeda Garrido (Hospital General De Enfermedades); Maria-Lorena Aguilera-Arevalo, Alvaro Eduardo Alvarez Rivera, Elvis Benjamin Bamaca Ixcajoc, Lilian Elizabeth Barreda Zelaya, Patricia Chacòn-Herrera, Ligia Margarita Corea Ruiz, Guillermo Echeverria-Davila, Mario Garcia, Danilo García, Edgar Fernando Gutiérrez Mayen, Noriega José, Nery Mazariegos, Diego Méndez, Michael Paniagua Espinoza (Hospital General San Juan De Dios).

Hungary: Zsolt Baranyai, David Bardos, Marton Benke, Kristof Illes, Balint András Kokas, Réka Szabó (1st Department of Surgery - Semmelweis University).

India: Akhila Appukuttan, Anjitha Asok, Vijaykumar D.k (Amrita Institute of Medical Sciences Hospital); Kapil Malik, Praveen Ravishankaran, Ritesh Tapkire (Cachar Cancer Hospital and Research Centre); Guru Moorthy, Joyner Abraham, Ramesh Muthuvel (Government Rajaji Hospital); John Alapatt, Abhay Kattepur, Nizamudheen Pareekutty (Malabar Cancer Centre); Mebanshanbor Garod, Caleb Harris, Cliff Wanniang (North Eastern Indira Gandhi Regional Institute of Health and Medical Sciences (NEIGRIHMS)); Ashish Gupta, Deepak Nehra, Sanjeev Parshad (Pandit Bhagwat Dayal Sharma Post Graduate Institute of Medical Sciences); Rajgopal Acharya, Rajendra Badwe, Manish Bhandare, Urvashi Jain, Karishma Kirti, Nita Nair, Shailesh Shrikhande, Purvi Thakkar (Tata Memorial Centre); Premkumar Anandan, Archana C S, Arun Holenarasipur Narasannaiah, Tejaswi Jagarlamudi, Sunil Kumar Venkatappa, Rashmi M R, Mallikarjuna Manangi, Abhishek Raghavendra, K. Seshagiri Rao, Vinay S, Vinay Sajjan, Aneesh Shenoy, Santhosh Shivashankar Chikkanayakanahalli, Kavya Tharanath, Sushmita V (Victoria Hospital).

Indonesia: Peter Adidharma, Raksheeth Agarwal, Radhian Amandito, Phebe Anggita Gultom, Ghafur Rasyid Arifin, Matthew Billy, Zatira Elfizri, Alessa Fahira, Devi Felicia, Triana Hardianti Gunardi*, Nadya Johanna, Nadia Rahmadiani Nugrahadi, Sonar Soni Panigoro, Siti Rahmayanti, Retta Catherina Sihotang (Dr Cipto Mangunkusumo National General Hospital); Santi Yuanita Brata, Hadi Winoto (Mardi Rahayu Hospital).

Iran, Islamic Rep.: Nastaran Barati, Manoochehr Karami, Hamidreza Khorshidi, Homa Naderifar (Besat Hospital).

Iraq: Mazin A. Abdulla (Basra Teaching Hospital).

Ireland: Maggie Coleman, Ronan J Doherty, Rob Hannon (Beacon Hospital); Brenda Murphy, Aine Stakelum, Des Winter (St Vincent’s University Hospital); Lylas Aljohmani, Richard Farnan, Yeshey Seldon, Tanna Tan, Shriya Varghese (St. James’s Hospital); Mohammad Alherz, Muaaz Ather, Mohammad Bajilan, Vivien Graziadei, Isobel Pilkington, Omar Quidwai, Paul Ridgway, Haaris Shiwani*, Abd al-Rahman Tahir (Tallaght Hospital); Eimear Blunnie, Daniel Burke, Niall Kennedy, Kate Macdonagh, Maeve O’Neill, Siobhan Rooney (University Hospital Galway).

Italy: Giuseppe Falco, Guglielmo Ferrari, Simone Mele, Gabriela Elisa Nita, Lara Ugoletti, Maurizio Zizzo (Arcispedale Santa Maria Nuova); Gianmaria Confalonieri, Giovanni Pesenti, Fulvio Tagliabue (ASST di Lecco - P.O di Lecco); Gianluca Baronio, Deborah Ongaro, Giacomo Pata (ASST Spedali Civili, Ospedale di Brescia); Bruno Compagnoni, Renato Salvadori, Lucio Taglietti (ASST Valcamonica Ospedale di Esine); Nicola D’Alessandro, Pierpaolo Di Lascio, Giovanni Pascale (Azienda Ospedaliera Regionale ‘San Carlo’); Luca Bortolasi, Tommaso Campagnaro, Massimo Carlini, Giorgio Lisi, Davide Lombardi, Corrado Pedrazzani, Domenico Spoletini, Giulia Turri, Paola Violi (Azienda Ospedaliera Universitaria Integrata di Verona); Donato Francesco Altomare, Fabrizio Aquilino, Nicola Musa, Vincenzo Papagni, Arcangelo Picciariello, Leonardo Vincenti (Azienda Ospedaliero Universitaria Consorziale Policlinico Di Bari); Dario Andreotti, Savino Occhionorelli, Matteo Tondo (Azienda Ospedaliero-Universitaria Di Ferrara); Stefano Maria Massimiliano. Basso (Azienda Per L’assistenza Sanitaria N. 5 Friuli Occidentale); Riccardo Cirelli, Marco Enrico Mario Maino, Guglielmo Niccolò Piozzi (Casa di Cura Igea); Emanuele Picone, Rosa Scaramuzzo, Giovanni Sinibaldi (Fatebenefratelli Isola Tiberina); Alfonso Amendola, Lorenzo Anastasio, Luigi Bucci, Emanuele Caruso, Antonio Castaldi, Sara Di Maso, Vincenza Paola Dinuzzi, Giovanni Esposito, Maria Gaudiello, Mariano Cesare Giglio, Paola Antonella Greco, Gaetano Luglio, Andrea Manfreda, Ester Marra, Federica Mastella, Gianluca Pagano, Roberto Peltrini, Vincenzo Pepe, Michele Sacco, Viviana Sollazzo, Giovanni Spiezio (Federico II University of Naples); Ettore Cianchetti, Nunzia Menduni (Hospital G.Bernabeo); Michele Maria Carvello, Francesca Di Candido, Antonino Spinelli (Humanitas Research Hospital); Fabio Corsi, Luca Sorrentino (ICS Maugeri); Fabio Marino (IRCCS ‘Saverio de Bellis’); Emanuele Luigi Giuseppe Asti, Luigi Bonavina, Emanuele Rausa (IRCCS Policlinico San Donato); Martina Asta, Andrea Belli, Francesco Bianco, Carmela Cervone, Paolo Delrio, Armando Falato, Andrea Fares Bucci, Rita Guarino, Ugo Pace, Daniela Rega (Istituto Nazionale Tumori Fondazione, Pascale-I.R.C.C.S.); Emilia De Luca, Gaetano Gallo, Giuseppe Sammarco, Giuseppe Sena, Giuseppina Vescio (Mater Domini University Hospital); Letizia Santandrea, Giampaolo Ugolini, Davide Zattoni (Ospedale degli Infermi di Faenza); Nicola Chetta, Gaetano Logrieco, Serafino Vanella (Ospedale Generale Regionale F. Miulli); Gianluca Garulli, Nicola Zanini (Ospedale Infermi di Rimini); Andrea Bondurri, Francesco Cammarata, Francesco Colombo, Diego Foschi, Giulia Maria Beatrice Lamperti, Anna Maffioli, Gianluca Matteo Sampietro, Al’ona Yakushkina, Gloria Zaffaroni (Ospedale Luigi Sacco Milano); Luca Ansaloni, Enrico Cicuttin, Maria Grazia Sibilla (Ospedale M. Bufalini); Harmony Impellizzeri, Marco Inama, Gianluigi Moretto (Ospedale Pederzoli); Sylvie Mochet, Elisa Ponte, Antonella Usai (Ospedale Regionale Umberto Parini); Stefano Mancini, Andrea Sagnotta, Luigi Solinas (Ospedale San Filippo Neri); Elisa Bolzonaro, Nicolò Tamini (Ospedale San Gerardo); Gianluca Curletti, Raffaele Galleano, Michele Malerba (Ospedale Santa Corona, Pietra Ligure (SV)); Sofia Campanella, Gianfranco Cocorullo, Francesco Colli, Paolino De Marco, Nicolò Falco, Tommaso Fontana, Leonel jospin Kamdem Mambou, Antonella La Brocca, Leo Licari, Brenda Randisi, Giovanna Rizzo, Giulia Rotolo, Giuseppe Salamone, Roberta Tutino, Paolina Venturelli (Policlinico Paolo Giaccone di Palermo); Stefano Malabarba, Alessandro Sgrò, Ivan Vella (Policlinico San Matteo); Bruno Cirillo, Daniele Crocetti, Giorgio De Toma, Pierfrancesco Lapolla, Andrea Mingoli, Paolo Sapienza (Policlinico Umberto I); Angela Belvedere, Stefania Bianchini, Margherita Binetti, Arianna Birindelli, Valeria Tonini (S.Orsola-Malpighi Hospital); Mauro Podda, Fabio Pulighe (San Francesco Hospital); Michele De Rosa (San Giovanni Battista Hospital); Lorenzo Bono, Felice Borghi, Paolo Geretto, Maria Carmela Giuffrida, Corrado Lauro, Alessandra Marano, Luca Pellegrino, Paola Salusso, Diego Sasia (Santa Croce and Carle Hospital); Michela Campanelli, Alberto Realis Luc, Mario Trompetto (Santa Rita Clinic, Vercelli); Roberto Cardia, Nicola Cillara, Antonio Nicola Giordano (Santissima Trinità - ATS Sardegna); Antonio Costanzo, Mario Alessandro Giovilli, Luca Turati (Treviglio Hospital); Silvestro Canonico, Gianluca Pellino, Guido Sciaudone, Francesco Selvaggi, Lucio Selvaggi (Universitá della Campania ‘Luigi Vanvitelli’, Naples).

Jordan: Nader Albsoul, Ahmad AlBsoul, Ala’a Aldeen Alkhatib, Osama Alsallaq, Justin Z. Amarin, Rami Ayoub, Isam Bsisu*, M S El Muhtaseb, Mohammad Jabaiti, Jamal Melhem, Ibrahim Nour, Yasmeen Z. Qwaider, Mohammad Hasan Salameh, Ahmad Suleihat, Haya H. Suradi (Jordan University Hospital); Mohammad Alammarin, Almoutuz Aljaafreh, Mohammad Bani hani*, Zeina Bani hani, Farah Bani Hani, Toqa Fahmawee, Shadi Hamouri, Cyrine Katanani, Ra’fat Tawalbeh, Tamara Tawalbeh, Hassan Zawahrah (King Abdullah University Hospital); Mohamad K. Abou Chaar, Lana Abusalem, Mahmoud Al-Masri*, Hani Al-Najjar, Lutfi Barghuthi (King Hussein Cancer Center).

Kenya: Zahra Ahmed, Adnan Maulana, Omar Ngotho (Coast Provincial General Hospital); Charbel Kamau, Aruyaru Stanley Mwenda (Consolata Hospital); Fridah Bosire, Elizabeth Mwachiro, Robert Parker, Ian Simel, Kimutai Sylvester (Tenwek Hospital).

Libya: Abdulmunem Ahmed Mustafa Althini, Sofian Elbarouni, Aya Elseed Elbeshina, Ahmed Gwea, Ans Malek, Wedad Albashir Masoud Farag (Alkhadra Hospital); Abdulwahab Abdalei, Abu Baker Abdel Malik*, Areej Abo-khammash, Ma’aly Abuhlaiga, Nour Adnan, Marwa Albaggar, Asma Alfitory, Asma Aljanfi, Fakhruddin Almuzghi, Zohoor Altumei, Fatima Alzabti, Hana Ashoushan, Mohamed Assalhi, Joma Azzubia, Sondos Bnhameıda, Malik Delhen, Houssein Elshafei, Hana Elteir, Fatima Esbaga, Abdel Aziz Gobbi, Fatma Hamouda, Hamdan Hilan, Rania Ismail, Fieruz Jebran, Muataz Kasbour, Galia Maderi, Saja Mohammad, Burooj Mohammed, Habib Murtadi, Hamassat Mustafa, Mohamed Rajab, Sarah Trenba, Mariam Wafaa (Misurata Cancer Center); Eman Al Sagheir, Alabas Almigheerbi, Ahmed Alzahaf, Sumayyah Ghayth Bahroun*, Najah Ben Dallah, Mahmoud Elshaibani, Haitem Eswaye, Maha Karar, Samah Omar, Eman Younes, Maha Younes, Dafer Zreeg (National cancer institute); Saleh Abujamra, Firas Ashour, Mala Elgammudi, Wesal Omar F. Aljadidi, Enas Saddouh, Randa Sharif (New Bridge Hospital); Aya Alabuzidi, AbdulMawlay Alwerfally, Sarra Aribi, Fatma Bibas, Taha Elfaituri, Yasmine Elhajjaji, Ala Khaled, Wegdan Khalil, Tesneem Layas, Enas Soula, Ahmed Tarek (Tripoli Central Hospital); Muad fathi khalleefah Abu hallalah, Saleh Abujamra, Hazem Abdelkarem Ahmed*, Tagwa Alsharef, Abdulsalam Ali Ben Saoud, Tasnim El Gharmoul, Ahmed Elhadi, Safa Elrais, Abdulhalim Shebani, Heba Zarti, Asaid Zeiton (Tripoli Medical Center).

Lithuania: Marijus Ambrazevicius*, Nerijus Kaselis, Migle Stakyte (Klaipeda Republic Hospital); Oleg Aliosin, Agne Cizauskaite, Sarunas Dailidenas, Vitalijus Eismontas, Migle Kybransiene, Vitalija Nutautiene, Narimantas Samalavicius, Dainius Simcikas*, Algirdas Slepavicius, Albinas Tamosiunas, Nerijus Ubartas, Paulius Zeromskas (Klaipeda University Hospital); Saulius Bradulskis, Edvinas Dainius, Juozas Juočas, Egle Kubiliute, Juozas Kutkevičius, Aurimas Opolskis, Audrius Parseliunas, Andrejus Subocius, Donatas Venskutonis, Egle Virbickaite, Diana Zuikyte (Lithuanian University of Health Sciences Kaunas Clinical Hospital); Algirdas Bogusevicius, Kristina Buzaite, Daiva Čepulienė, Ieva Cesleviciene, Vaidotas Cesna, Jolanta Gribauskaite, Povilas Ignatavicius, Mantas Jokubauskas, Monika Liugailaitė, Ernest Margelis, Ruta Mazelyte, Lina Pankratjevaitė, Matas Pažusis, Agne Rackeviciute, Justina Saladyte, Monika Škimelytė, Vygintas Šlenfuktas, Monika Sudeikyte, Algimantas Tamelis, Tomas Vanagas*, Žygimantas Žumbakys (Lithuanian University of Health Sciences Kaunas Clinics); Aivaras Atkociunas, Audrius Dulskas, Justas Kuliavas (National Cancer Institute); Justas Birutis*, Sigitas Paškevičius, Mindaugas Šatkauskas (Republican Siauliai County Hospital); Donatas Danys, Matas Jakubauskas, Lina Jakubauskiene, Marius Kryzauskas, Vytautas Lipnickas, Gabija Makūnaitė (Vilnius University Hospital).

Madagascar: Fanjandrainy Rasoaherinomenjanahary, Herizo Rasolofonarivo, Luc Hervé Samison* (Joseph Ravoahangy Andrianavalona Hospital).

Malawi: Bitiel Banda, Precious Malemia, Vanessa Msosa (Kamuzu Central Hospital).

Malaysia: Ahmad Imran Ahmad Izzuddin, Andre Das (Hospital Kajang); Ying Yee Gan, Tan Shong Sheng, Jia yng Siaw (Hospital Sibu); Mohd Fadliyazid Ab Rahim, Dyg Zahratul Hamrak Abang Jamari, Nurfariza Che Husin, Muhd Yusairi Kamarulzaman, Yi Ping Lim, Nil Amri Mohamed Kamil, Mohd Razeen Mohd Hassan, Saidah Mohd Sahid*, Johari Mustafa, Elaine Hui Been Ng, Wan Khamizar Wan Khazim (Hospital Sultanah Bahiyah); Ng Chang Ern, P.g. Lingeshan, Syariz Ezuan Sulaiman (Hospital Sultanah Nora Ismail); Sue Ean Ang, Muhammad Navid Bin Mohamad Sithik, Yih Jeng Cheong, Mahadevan Deva Tata, Law Jia Xian, Aravinthan Kadravello, I-Ern Koh, Li-Yen Ng, Yuki Julius Ng We Yong, Kandasami Palayan, Chi Xuan Sam, Phuah Siow Jin, Jeremy Tan Ern Hwei, Yita Tang, Alvin Zubin Ter (Hospital Tuanku Ja’afar); Michael Pak-Kai Wong, Andee Dzulkarnaen Zakaria, Zaidi Zakaria (Hospital Universiti Sains Malaysia); Fitjerald Henry, Thyivya Kalaiselvan (Selayang Hospital); Muhammad Fairuz Shah Abd Karim, Mohamed Rezal Abdul Aziz, Nora Abdul Aziz, Tak Loon Khong, Peng Choong Lau, Hiong Chin Lim, April Camilla Roslani*, Jonathan Chen Ken Seak, Sui-Weng Wong, Lai Fen Wong, Leow Yeen Chin (University Malaya Medical Centre).

Malta: Mercy Chinemerem Anyanwu, Elaine Borg, Zachary Busuttil, Thomas Calleja, Kurt Lee Chircop, Ruth Cutajar, Andrew Michael Dimech, Sarah Ellul, Joseph Galea, Kiara Gascon Perai, Ruth Gatt, Lisa Kelman, Elizabeth Micallef, Favour Nwolu, Kim Sammut, Joanna Thompson, Sean Warwicker, Matthew Zammit (Mater Dei Hospital).

Mexico: Fernando Cordera, Efraín Cruz González, Jorge Sánchez-García (ABC Medical Center); Francisco José Barbosa Camacho, Francisco Javier Barrera López, Carlos Jose Zuloaga Fernandez del Valle (ANKER Oncologia Global Especializada); Eric Acosta, Iván Romarico González Espinoza, Perla Moreno (Hospital Angeles Puebla); Ana Olivia Cortes-Flores, Clotilde Fuentes Orozco, Alejandro Gonzalez Ojeda (Hospital de Especialidades, CMNO-IMSS); Samantha Corro Díaz González, Laura Martinez, Antonio Ramos-De la Medina (Hospital Español Veracruz); Bonifacio Mosqueda Amador, Armando Novoa, Dennet Arturo Olazo Espejo (Hospital Regional de Alta Especialidad); Alejandro Jimenez, Federico Lopez Rosales, Elva Gabriela Vanoye (Hospital Regional de Alta Especialidad de la Península de Yucatán); Luis Alberto Garcia Gonzalez, Roberto Carlos Miranda-Ackerman, Manuel Solano-Genesta (Hospital San Javier); Alethia Alvarez-Cano, Hector Hugo Romero-Garza (Hospital Universitario Dr José Eleuterio González); Heriberto Medina- Franco, Lorelí Mejía-Fernández, Noel Salgado-Nesme, Omar Vergara-Fernandez (Instituto Nacional de Ciencias Médicas y Nutrición ‘Salvador Zubirán’); Guadalupe Montserrat Gutiérrez-Mota, Francisco Xavier Hernandez Vera, Anabella Llantada Lopez, Gilberto Morgan Villela, Felipe de Jesus Ramirez Padilla, Walezka Tapia Marin (Morgan Oncología Soluciones Integrales); Mónica Martínez Maldonado, Ramses Sánchez Suárez, José Manuel Troche (Unidad de Medicina de Alta Especialidad ‘Adolfo Ruiz Cortines’).

Morocco: Chaymae Benyaiche, Oumaima Outani (Centre Hospitalier Universitaire Ibn Sina Rabat); Souadka Amine, Amine Benkabbou, Anass Mohammed Majbar, Raouf Mohsine, Ali Rafik (Institut National d’Oncologie).

Myanmar: Thida Oung, Moe Moe Tin (North Okkalapa General Hospital).

Namibia: David W Borowski, Philipp Plarre (Mediclinic Cottage Hospital); David W Borowski, Philipp Plarre (Welwitschia Hospital).

Netherlands: Anna Alberga, Nina Sluiter, Jurriaan Tuynman (Amsterdam UMC VUmc); Robin Blok, Didem Cömert, Roel Hompes, Marianne Kalff, Merel Elisabeth Stellingwerf, Pieter Tanis, Mark van Berge Henegouwen, Elise Maria van Praag, Daan Wisselink (Amsterdam UMC, University of Amsterdam); Michael Gerhards, Josephine Lopes Cardozo, Emma Westerduin (Onze Lieve Vrouwe Gasthuis); Joske de Jonge, Aaw van Geloven, Kaz van Schilt (Tergooi Hospital); Frank den Boer, Simone Stoots, Stijn Vlek (Zaans Medisch Centrum).

New Zealand: Jamie Adams, Ibrahim S. Al-Busaidi, Gabrielle Budd, Seung il Choi, Michael Jen Jie Chu, Anurag Ganugapati, Lucy McKinstry, Rebecca Pascoe, Simon Richards, Kenrick Rosser, Annie Stevenson, Rebecca White (Christchurch Hospital); Shebani Farik, Jin Kwun, Ahmed Murad (North Shore Hospital); Sarah Cowan, Timothy Hall, Michael Hayton (Taranaki Base Hospital).

Niger: Laminou Malam Sani (Agadez Hospital); Souleymane Oumarou Garba* (Zinder Mother Child Center); Harissou Adamou, Ibrahim Amadou Magagi*, Oumarou Habou (Zinder National Hospital).

Nigeria: Halima Aliyu, Muhammad Daniyan, Tunde T. Sholadoye (Ahmadu Bello University Teaching Hospital); Lawal Abdullahi, Lofty-John Anyanwu*, Aminu Mohammad Mohammad, Abubakar Bala Muhammad, Abdurrahman Abba Sheshe, Ibrahim Suleiman (Aminu Kano Teaching Hospital); Alaba Adesina, Ajibola Awolowo, Clement Onuoha, Omotayo Salami, Ogechukwu Taiwo*, Agboola Taiwo (Babcock University Teaching Hospital); Stephen Kache, Jerry Godfrey Makama, Danjuma Sale (Barau Dikko Teaching Hospital); Olajide Abiola, Akinlabi Ajao, Anthony Ajiboye (Bowen University Teaching Hospital); Amarachukwu Etonyeaku, Julius Olaogun* (Ekiti State University Teaching Hospital); Ademola Adebanjo, Opeoluwa Adesanya (Federal Medical Centre); Michael Olatunji Afolayan, Olanrewaju Balogun, Ayomide Makanjuola, Samuel Nwokocha, Rufus Wale Ojewola, Thomas Olagboyega Olajide* (Lagos University Teaching Hospital); Adewale Aderounmu, Abdul-Rashid Adesunkanmi, Adewale Adisa*, Augustine Agbakwuru, Adeleke Akeem Aderogba, Olusegun Isaac Alatise, Olukayode Arowolo, Oladejo Lawal, Tajudeen Mohammed, Chinedu Ndegbu, Olalekan Olasehinde, Funmilola Wuraola (Obafemi Awolowo University Teaching Hospitals Complex); Akinbolaji Akinkuolie, Amarachukwu Etonyeaku, Arinzechukwu Mosanya (Obafemi Awolowo University Teaching Hospitals Complex Wesley Guild Hospital Unit); Omobolaji Ayandipo, Peter Elemile, Taiwo Akeem Lawal (University College Hospital); Samuel Ali SANI, Stephen Garba, Rebecca Hauwa SANI, Samson Olori*, Henry Onyebuashi, Ifeanyi Umoke (University of Abuja Teaching Hospital); Adedire Adenuga, Ademola Adeyeye*, Olufemi Habeeb, Bashir Lawal, Abdulrasheed Nasir (University of Ilorin Teaching Hospital).

Norway: Eirik Kjus Aahlin, Didrik Kjønås, Elisabeth Myrseth (University Hospital Of North Norway).

Pakistan: Jibran Abbasy, Abdul Alvi, Omair Saleem (Aga Khan University); Asma Afzal, Anam Nazir (Ganga Ram Hospital); Muhammad Farooq, Ayesha Liaqat, Syed Asghar Naqi*, Ali Raza, Muzna Sarfraz, Muhammad Sarwar (King Edward Medical University, Mayo Hospital, Lahore); Muntaha Banglani, Ambreen Munir, Rahmat Sehrish (Liaquat University of Medical & Health Sciences); Bushra Ayub, Raza Sayyed (Patel Hospital); Amna Altaf, Saima Ayub, Ahmad Uzair Qureshi, Komal Saeed, Bilal Syed (Services Hospital Lahore); Sana Amir Akbar, Abdul Wahid Anwer, Ruqayya Naheed Khan*, Amina Iqbal Khan, Shahid Khattak, Sameen Mohtasham, Muhammad Asad Parvaiz, Aamir Ali Syed (Shaukat Khanam Memorial Cancer Hospital and Research Centre); Abdul Basit Ansari, Noman Shahzad (Sindh Institute of Urology and Transplantation); Tanwir Khaliq, Isbah Rashid, Shahzad Hussain Waqar (The Pakistan Institute of Medical Sciences).

Palestine: Hasan Abu Al-saleem, Amjad Abu Alqumboz, Mohammad Alqadi, Adham Amro, Rawan Assa, Eman Awesat, Rawan Ayyad, Mohammed Hammad, Ayat Haymony, Bassel Hijazi, Bara Hmeidat, Rowaa Lahaseh, Aseel Qawasmi, Alaa Rajabi, Mohammed Shehada, Sundus Shkokani, Yasmine Yaghi, Nadine Yaghi (Al Makassed Islamic Charitable Society Hospital Jerusalem); Mohammad AlZohour, Mohammad Farid, Yousef Mahmoud Habes, Wesam Juba, Yanal Nubani, Abdelrahman Rabee, Mohammad Sa’deh (Al-Ahli Hospital); Saeed Abed, Iyad Al basos, Mohammad Alswerki, Dina Ashour, Israa Awad, Samar Diab, Alaa El Jamassi, Sahar El-Kahlout, Somaya Elhout, Ahmed N K Hajjaj, Doaa Hasanain, Baraa Nabil hajjaj, Mohammed Obaid, Eman Saikaly, Ahmed Salhi (Al-Shifa Hospital); Hiba Al-Tammam, Murad Almasri, Muath Baniowda, Doha Beshtawi, Ali Horoub, Rami Misk, Bayan Mohammad, Rami Qasrawi, Tasnim Sholi (An-Najah National University Hospital); Samar Abu-Nimeh, Abrar Abu-srour, Sadi A. Abukhalaf, Samer Adawi, Barah Alsalameh, Kholoud Ayesh, Muawiyah Elqadi, Ahmad Hammouri, Fatima Karim Mustafa, Natalie Marzouqa, Shatha Melhem, Dima Miqdad, Balqees Mohamad, Mhammed Rawhi (Beit Jala Governmental Hospital (Al Hussein)); Ayman B. Abu Ahammala, Ahmed Abu Ataya, Israa Abu Jayyab, Samar Al-Shwaikh, Othman Alagha, Mohammed Alasttal, Haneen Awadallah, Mahmood Elblbessy, Jehad Fares, Akram Jarbou, Ibtisam Mahfouz, Moath A. Albahnasawi (European Gaza Hospital); Asmaa’ Abo mahadi, Hasan Abuelhatal, Ayham Abuelqomboz, Abdelrahman Almoqayyad, Abdallah Alwali, Reem Balaawi, Mahmoud Hamouda, Mohammed Humeid, Abdullah Jedyan, Tasneem Mahmoud Abu hamam, Ghadeer Matar, ALi Salem, Tahani Samra, Nureddin Shaheen, Karam Shihada (Indonesian Hospital); Ayoob A.Nemer, Mahmoud Abu Al Amrain, Abdulwhhab Abu Alamrain, Najlaa Abu Jamie, Mohammed R. Abu-Rous, Nada Alfarra, Mohammed AlTaweel, Noor Alwhaidi, Ramadan Hamed, Bader Saqqa, Ahmad Shaheen (Nasser Hospital); Dana Aljaber, Loay Aljaberi, Malak Alwaheidi, Assef Jawaada, Hani Khaldi, Rami Qahoush, Jalil Qari, Rana Saadeh, Ahlam Salim, Aseel Yacoub (Palestine Medical Complex); Abbas Abbas, Rana Abu shua`ib, Baraa Abu Zainah, Mahmoud AbuSirrees, Basheer Babaa, Ola Barhoush, Asef Belal qadomi, Laith Daraghmeh, Reema Haji, Alaa Khatatbeh, Lana Khatib, Salsabeel Qarariah, Yara Quzmar, Khalil Safadi, Roqaya Salameh (Rafidia Hospital); Mohammad Hassan, Shifaa Herzallah, Loai Massad, Ahmed Nazzal, Ranin Nazzal (The Martyr Dr. Khalil Sulaiman Hospital (Jenin Governmental Hospital)).

Paraguay: Dennis Escobar, Gustavo Miguel Machain V, Agustin Rodriguez Gonzalez (Hospital de Clínicas, II Cátedra de Clínica Quirúrgica, Universidad Nacional de Asunción).

Peru: Jorge Emerson Chachaima Mar, Nathaly Olga Chinchihualpa Paredes, Vicente Cuba, Walter Lopez, Maria Milagros Niquen Jimenez*, Nestor Alberto Sanchez Bartra, Olenka Sapallanay Ojeda, Diego Sequeiros, Andrea Toscano Pacheco, María Vergara (Arzopispo Loayza National Hospital); Sol Abarca, Rodrigo Alcorta, Giuliano Borda-Luque, Ivan Edward Eusebio Zegarra, Claudia Luján López, Mirella Marrufo, Cinthya Mogrovejo, Andrea Nomura, Yamile Rodríguez Angeles, Maitza Rosario Vidal Meza, Gabriela Zavala* (Cayetano Heredia National Hospital); José Neiser Castillo Arrascue, Jomara Caroline Hidrogo Cabrera, José Julio Mariano Larrea vera, Miguel Osorio, Edgar Alcides Ylatoma Díaz* (Hospital Nacional Almanzor Aguinaga Asenjo).

Philippines: Mark Anthony Fontanilla, Joseph Roy Fuentes, Anna Leah Salazar (José R. Reyes Memorial Medical Center); Genieve Dominguez, Marc Paul Lopez, Shiela Macalindong, Mark Augustine Onglao, Arjel Ramirez, Marie Dione Sacdalan, Mayou Martin Tampo, Gemma Leonora Uy (Philippine General Hospital, University Of The Philippines Manila); Jeremiah Mangahas, Kenneth Yabut (Quirino Memorial Medical Center); Joannes Paul Cañete, Bernalynn Eris Cansana, Ernes John Castro, Maria Kaiserin Lipana, Manuel Francisco Roxas, Vlu Jean Zara (The Medical City).

Poland: Maciej Chroł, Paula Franczak, Michał Orłowski (Ceynowa Hospital); Piotr Budzyński, Andrzej Budzyński, Pawel Bury, Agata Czerwińska, Jadwiga Dworak, Jacek Dziedzic, Michał Kisielewski, Jan Kulawik, Anna Lasek, Piotr Major, Piotr Małczak, Marcin Migaczewski, Michał Pędziwiatr, Magdalena Pisarska, Dorota Radkowiak, Mateusz Rubinkiewicz*, Anna Rzepa, Tomasz Skoczylas, Maciej Stanek, Katarzyna Truszkiewicz, Mateusz Wierdak, Marek Winiarski, Piotr Zarzycki, Anna Zub-Pokrowiecka (Jagiellonian University Medical College); Piotr Kowalewski, Rafał Roszkowski, Maciej Walędziak (Military Institute Of Medicine).

Portugal: Miguel Tomé, Sara Patrocinio, Ines Guerreiro* (Centro Hospitalar Barreiro Montijo, EPE); Filipe Almeida, Xavier de Sousa*, Nuno Monteiro (Centro Hospitalar de Setúbal); Maria Teresa Costa Santos*, Daniela de Oliveira, Marta Lopes Serra, Daniela Morgado, Christian Neves, Ana Carolina Oliveira, Alice Pimentel, Sofia Silva (Centro Hospitalar do Baixo Vouga); Márcia Carvalho* (Centro Hospitalar do Medio Ave); Lúcia Carvalho, Joana Magalhães, Leonor Matos* (Centro Hospitalar Entre o Douro e Vouga); Tânia Monteiro, Carlota Ramos*, Vanessa Santos (Centro Hospitalar Lisboa Norte); José Barbosa, Jose Costa-Maia, Vítor Devezas, Ana Fareleira, Cristina Fernandes, Diana Gonçalves, Henrique Mora, Marina Morais*, Fabiana Silva de Sousa (Centro Hospitalar Sao Joao); Sara Catarino Santos*, Ana Logrado, André Tojal (Centro Hospitalar Tondela-Viseu); Edgar Amorim, Miguel F. Cunha*, Ana Fazenda, João Pedro Melo Neves, Inês Isabel Sampaio da Nóvoa Gomes Miguel, Diogo Veiga (Centro Hospitalar Universitario do Algarve); José Azevedo, Hugo Cardoso Louro*, Mariana Leite (Centro Hospitalar Vila Nova de Gaia/Espinho); José Azevedo*, Maria Bairos Menezes, Bárbara Gama (Hospital da Horta, E.P.E.); Diana Brito, Marta Cristina Cruz Martins, André Graça e Magalhães, Ana Catarina Longras*, Rita Lourenço, Diana Matos (Hospital da Senhora da Oliveira); Luis Castro, Filipa Policarpo, Joana Romano* (Hospital de Egas Moniz); Mariana Leite, Cristina Monteiro*, Diogo Pinto (Hospital de Santa Luzia); Marina Duarte, Sónia Fortuna Martins*, Mariline Oliveira (Hospital de Santarem); Diogo Galvão, Lisandra Martins, Anaisa Silva, Viorel Taranu, Bárbara Vieira* (Hospital de Santo Espirito da ilha Terceira); Jessica Neves*, Simone Oliveira, Hugo Ribeiro (Hospital Distrital da Figueira da Foz); Margarida Cinza, Rosa Felix, Arnaldo Machado, Joana Oliveira, Joana Patrício*, Rita Pedroso de Lima, Mário Pereira, Miguel Rocha Melo, Cristina Velez (Hospital do Espirito Santo); Alberto Abreu da Silva, Mariana Claro*, Daniel Costa Santos, Andreia Ferreira (Hospital do Litoral Alentejano); Hugo Capote, Daniela Rosado, Filipa Taré (Hospital Doutor José Maria Grande); Oriana Nogueira, Miguel Ângelo, José Miguel Baiao, Andreia Guimarães, João Marques, Miguel Nico Albano*, Marta Silva, Ana Valente da Costa, Teresa Vieira Caroço (Hospital Geral - Centro Hospitalar de Coimbra); Sara Almeida Braga, Ines Capunge, Marta Fragoso*, João Guimarães, Bruno Pinto, João Ribeiro (Hospital Prof. Doutor Fernando Fonseca, E.P.E.); Miguel Angel, Guilherme Fialho*, Monica Guerrero (Hospital Santa Luzia Elvas); Filipa Campos Costa, Diogo Cardoso, Vasco Cardoso (Hospital Sao Francisco Xavier); Magda Alves, Inês Estalagem, Tiago Louro, Cláudia Marques*, Rita Martelo, Miguel Morgado (Hospital Vila Franca de Xira); Rita Canotilho, Ana Margarida Correia, Pedro Martins, Mariana Peyroteo* (IPO Porto); João Gomes, Rita Monteiro, Manuela Romano* (Unidade Local de Saúde de Castelo Branco); Daniela Macedo Alves, Rita Peixoto, Catarina Quintela* (Unidade Local de Saude de Matosinhos - Hospital Pedro Hispano); Maria João Jervis, Débora Melo, André Pacheco, Valter Paixão, Vera Pedro, Joana Pimenta, João Pimenta de Castro*, Ana Rocha (Unidade Local de Saude do Baixo Alentejo).

Romania: Mircea Beuran, Matei Razvan Bratu, Cezar Ciubotaru, Bogdan Diaconescu, Sorin Hostiuc, Ionut Negoi, Bogdan Stoica, NA (Emergency Clinical Hospital Bucharest).

Russian Federation: Evgeny Anokhin, Georgy Kuznetsov, Giorgi Oganezov, Fedor Paramzin, Ekaterina Romanova, Valeryan Rutkovskii, Vasilii Rutkovskii, Mikhail Shushval, Mikhail Zabiyaka (Kaliningrad Regional Hospital); Khasan Dzhumabaev, Valerii Ivanov, Zaman Mamedli (N.N.Blokhin Russian Cancer Research Center); Sergey Achkasov, Artem Balkarov, Elnur Nabiev, Marat Nagudov, Evgeny Rybakov, Karina Saifutdinova, Oleg Sushkov, Armen Vardanyan* (State Scientific Centre of Coloproctology).

Rwanda: Ainhoa Costas-Chavarri, Lule Joseph, Isaac Ndayishimiye (Rwanda Military Hospital); Jc Allen Ingabire, Ntirenganya Faustin, Alphonse Zeta Mutabazi, Jean Paul Mvukiyehe, Vizir J.P Nsengimana, Carine Uwakunda (University Teaching Hospital of Kigali).

Saudi Arabia: Mohammad Monir Abbas, Nouf Akeel, Murad Aljiffry, Kholoud Awaji, Ali Farsi, Ghader Jamjoum, Ahmad Khoja, Ashraf Maghrabi, Nadim Malibary, Mohammed Nassif, Abdulaziz Saleem, Abdullah Sultan, Wail Tashkandi, Hanaa Tashkandi, Nora Trabulsi (King Abdulaziz University Hospital).

Senegal: Mouhamadou Bachir Ba, Adja Coumba Diallo, Abdourahmane Ndong (Hopital Aristide Le Dantec).

Serbia: Vladica Cuk, Uroš Janković, Jovan Juloski (Zvezdara University Medical Center).

Singapore: Sharon Zhiling Koh, Frederick Koh, Kuok Chung Lee, Kai Yin Lee, Sean Lee, Wei Qi Leong, Bettina Lieske, Su Ann Lui, Prajwala Prakash (National University Hospital).

Slovenia: Jan Grosek, Gregor Norcic, Ales Tomazic (University Medical Centre).

South Africa: Nicolas Fitchat, Robert Jaich, Devorah Wineberg (Chris Hani Baragwanath Academic Hospital); Modise Zacharia Koto (Dr George Mukhari Academic Hospital); Daniella Baiocchi, Damian Clarke, Christina Johanna Steenkamp, Stephanie Van Straten (Greys hospital); Sharon Bannister, Adam Boutall, Galya Chinnery, Anna Coccia, Angela Dell, Parveen Karjiker, Christo Kloppers, Nicholas Loxton, Tumi Mabogoane, Francois Malherbe, Eugenio Panieri, Shreya Rayamajhi, Richard Spence, Tirsa van Wyngaard, Claire Warden (Groote Schuur Hospital); T E Madiba, Yoshan Moodley, Nivashen Pillay* (Inkosi Albert Luthuli Central Hospital); Savannah Brooks, Charlise Kruger, Lisa Hannah Van Der Merwe (Kalafong Academic Hospital); Ferhana Gool, Maahir Kariem (Mitchell’s Plain District Hospital); Heather Bougard, Kathryn Chu, Nazmie Kariem, Fazlin Noor, Reantha Pillay, Leandi Steynfaardt (New Somerset Hospital).

Spain: Lucía González González, José Miguel Marín Santos, Paula Martín-Borregón, Javier Martínez Caballero, Cristina Nevado García, Pastora Rodriguez Fraga (12 de Octubre University Hospital); Gonzalo De Castro Parga, Maria Pilar Fernández Veiga, Lucía Garrido López, Hugo Infante Pino, Irene Lages Cal, Marta López Otero, Manuel Nogueira Sixto, Marta Paniagua García Señorans, Laura Rodríguez Fernández, Alejandro Ruano Poblador, Erika Rufo Crespo, Raquel Sanchez-Santos, Vincenzo Vigorita (Álvaro Cunqueiro Hospital); Ester Alonso Batanero, Dorisme Asnel, Isabel Cifrian Canales, Elisa Contreras Saiz, Irene De Santiago Alvarez, Tamara Díaz Vico, Sebastian Fernandez Arias, Daniel Fernández Martínez, Carmen García Bernardo, Luis Joaquín García Flórez, Carmen Garcia Gutierrez, Manuel García Munar, Carlos Alberto Márquez Zorrilla Molina, Marta Merayo, José Luis Michi Campos, Maria Moreno Gijon, Jorge L. Otero-Diez, Jose Luis Rodicio Miravalles, Lorena Solar-Garcia, Aida Suárez Sánchez, Nuria Truan (Central University Hospital Of Asturias); Cristina Alejandre Villalobos, Yurena Caballero Díaz, Marta Jimenez, Dacil Montesdeoca, Antonio Navarro-Sánchez*, Victor Vega (Complejo Hospitalario Universitario Insular-Materno Infantil); Juan Beltrán de Heredia, Zahira Gómez, Carlos Jezieniecki, Ana Patricia Legido Morán, Mario Montes-Manrique*, Mario Rodriguez-Lopez, María Ruiz Soriano, Jeancarlos Trujillo Díaz, Andrea Vazquez Fernandez (Hospital Clínico Universitario de Valladolid); Nuria Argudo, Miguel Pera, Laia Torrent Jansà (Hospital del Mar); Melody García Domínguez, Ignacio Goded, Marta Roldón Golet, Issa Talal El-Abur, Alejandra Utrilla Fornals, Vanesa Zambrana Campos (Hospital General San Jorge); Maria Del Mar Aguilar Martinez, Marina Bosch, Luis García-Catalá, Luis Sánchez-Guillén (Hospital General Universitario de Elche); Eva Artigau, Nuria Gomez Romeu, David Julià Bergkvist (Hospital Universitari de Girona Dr. Josep Trueta); Beatriz Espina Perez, Olga Morató, Carles Olona (Hospital Universitari de Tarragona Joan XXIII); Beatriz Diéguez, Alexander Forero-Torres, Manuel Losada (Hospital Universitario del Sureste); Segundo Gomez-Abril, Paula Gonzálvez, Rosario Martinez, Sergio Navarro Martínez, Carmen Payá-Llorente, Álvaro Pérez Rubio, Sandra Santarrufina Martinez, Juan Carlos Sebastián Tomás, Ramon Trullenque Juan (Hospital Universitario Doctor Peset); Alberto Gegúndez Simón, Paloma Maté, Maria Isabel Prieto-Nieto, Ines Rubio-Perez, Aitor Urbieta, Marina Vicario Bravo (Hospital Universitario la Paz); David Abelló, Matteo Frasson, Alvaro Garcia-Granero (Hospital Universitario y Politécnico La Fe); Alfredo Abad Gurumeta, Ane Abad-Motos, Elena Lucena- de Pablo, Beatriz Nozal, Javier Ripollés-Melchor, Rut Salvachúa (Infanta Leonor University Hospital); Esther Ferrero, Luis Garcia- Sancho Tellez, Irene Ortega Vázquez, Antonio L. Picardo, Jose Alberto Rojo López, Laura Patricia Zorrilla Matilla (Infanta Sofía University Hospital); Carmen Cagigas Fernandez*, Sonia Castanedo Bezanilla, José Estevez Tesouro, Maria Jose Fernandez-Diaz, Juan García Cardo, Marcos Gomez Ruiz, Erik Gonzalez-Tolaretxipi, Jaime Jimeno Fraile, Cristobal Poch, Montserrat Rodriguez-Aguirre, Noemí Troche Pesqueira, Maria Soledad Trugeda-Carrera (Marqués de Valdecilla University Hospital); Javier de la Torre, Ruth Blanco- Colino, Eloy Espin-Basany, Martin Espinosa-Bravo, Clara Morales Comas, Eduardo Reyes Afonso, Joaquín Rivero Déniz, Christian Siso Raber, Mireia Verdaguer Tremolosa (Vall d’Hebron University Hospital).

Sri Lanka: Pramodh Chandrasinghe, Sumudu Kumarage, Nimeshi Wijekoon Arachchilage (North Colombo Teaching Hospital); Kithsiri Janakantha Senanayake (Teaching Hospital Anuradhapura).

Sudan: Ahmed Abdalla Ahmed Elkamel (Ibrahim Malik Teaching Hospital); Mohammed A. Adam (Soba University Hospital); Mahmoud Saleh (University of Gezira Hospital).

Sweden: Nina Blomme, Anders Thorell, Fredrik Wogensen (Ersta Hospital); Andreas Älgå*, Dhirar Ansarei, Fuat Celebioglu, Göran Heinius, Linda Nigard, Emil Pieniowski (South General Hospital); Sandra Ahlqvist, Ida Björklund, Yucel Cengiz, Andreas Frånberg, Martina Håkansson (Sundsvall Hospital); Karin Adamo, Oskar Franklin, Malin Sund, Rebecca Wiberg (Umea University Hospital); Yvette Andersson, Abbas Chabok, Maziar Nikberg (Västmanlands Hospital Västerås); Alexander Kugelberg (Vrinnevi Hospital).

Switzerland: Claudia Canonica, Dimitrios Christoforidis*, Fabrizio Fasolini, Paolo Gaffuri, Mauro Giuliani, Francesco Meani, Sotirios Georgios Popeskou, Silvia Pozza, Wiebke Wandschneider (Ente Ospedaliero Cantonale); Lorenz Peterer, Lukas Werner Widmer*, Bernd Zimmermann (Kantonsspital Graubunden); Panagiotis Bakoleas, Iris Chanousi, Lydia Charalampidou, Lukasz Filip Grochola, Franziska Heid, Sotirios Ntaoulas, Michail Outos, Georgios Peros*, Hanna Podolska-Skoczek, Katharina Beate Reinisch, Christian Zielasek (Kantonsspital Winterthur); Daniel Clerc*, Nicolas Demartines, Jérôme Gilgien, Amaniel Kefleyesus, Pénélope St-Amour, Arnaud Toussaint (Lausanne University Hospital CHUV).

Syrian Arab Republic: Maryam Alhimyar, Bayan Alsaid, Amr Alyafi (Al-Assad University Hospital); Ahmad Alkhaledi, Basel Kouz, Ahmad Omarain (Al-Bairouni University Hospital); Yusra Al-Sabbagh, Haya Alkhatib, Samer Sara (Al-Mouwasat University Hospital); Ahmad Alhaj, Aghyad Danial, Lama Kadoura (Aleppo University Hospital); Sarah Maa Albared, Yamen Monawar, Louei Nahas (Damascus Hospital); Barook Abd, Ahmad Saad, Habib Wakkaf (Tishreen University Hospital).

Tunisia: Hanen Bouaziz, Hatem Bouzaiene, Montassar Ghalleb (Institut Salah Azaiez).

Turkey: Elif Akaydin, Ata Cem Akbaba, Onur Atakul, Ege Baltaci, Sevval Besli, Gökçen Burgu, Ulukan Cenal, Cansu de Muijnck, Hasan Can Demirkaya, Alper Dogruoz, Zeynep Ipek Gezer, Yasemin Gündoğdu, Merve Kara, Hasan Kürşad Korkmaz, Gökalp Kağan Kurtoğlu, Volkan Ozben, Berk Baris Ozmen, Ahmet Murat Pektaş, Eda Kübra Sel, Nilüfer Yenidünya (Acibadem Atakent Hospital); Fuat Baris Bengur, Berke Mustafa Oral, Tahir Koray Yozgatli (Acibadem Maslak Hospital); Seymur Abdullayev, Mehmet Emin Gunes, Nuri Alper Sahbaz (Bakirkoy Dr. Sadi Konuk Training And Research Hospital); Tuba Banaz, Kübra Kargıcı, Omer Faruk Kuyumcu, Erkan Yanıkoğlu, Merve Yeşilsancak, Duygu Yılmaz (Cerrahpasa Medical Faculty Istanbul University); Melik Kagan Aktas, Ahmet Rencuzogullari (Cukurova University Faculty of Medicine); Arda Isik (Erzincan University Hospital); Sezai Leventoğlu, Ali Yalçınkaya, Osman Yüksel (Gazi University Medical Faculty Hospital); Mustafa U Kalaycı, Yası̇n Kara, Inanc Samil Sarici (Kanuni Sultan Suleyman Training and Research Hospital); Alp Akin, Gökçe nur Alemdağ, Ekin Arslan, Bahadir Emre Baki, Muhammed Selim Bodur, Adnan Calik, Bahar Candas Altinbas, İrem Cihanyurdu, Oğuz Erkul, Burak Gül, Ali Guner, Beyza Köse, Anil Semiz, Şule Sevim, Serkan Tayar, Kadir Tomas, Ozan yavuz Tüfek, Serdar Türkyılmaz, Mehmet Uluşahin, Arif Usta, Reyyan Yildirim (Karadeniz Technical University Farabi Hospital); Sertaç Ata Güler, Ozan Can Tatar, Ecenur Varol (Kocaeli University Teaching Hospital); Busenur Kirimtay, Muhammed Uysal, Alp Yildiz (Memorial Ankara Hospital); Emin Kose (Okmeydanı Training And Research Hospital); Ahmet Burak Ciftci, Elı̇f Çolak, Huseyin Eraslan, Gultekin Ozan Kucuk, Kürşat Yemez (Samsun Training and Research Hospital).

Uganda: Herman Lule* (Fort Portal Regional Referral Hospital); Mumbere Bienfait, Herman Lule* (Kampala International University Teaching Hospital); Emmanuel Bua, Matthew Doe*, Noella Okalany (Mbale Regional Referral Centre); Arianna Birindelli* (St. Kizito Hospital).

Ukraine: Maksym Basarab*, Oleksii Bielosludtsev, Maryna Freigofer, Kateryna Kolhanova, Kateryna Perepelytsia, Kateryna Romanukha, Dmytro Savenkov, Stanislav Siryi, Maksym Tereshchenko, Nezamai Viacheslav, Anton Volovetskyi (Dnepropetrovsk Regional Clinical Oncology Center); Andrey Kebkalo, Yegor Tryliskyy, Volodimir Tyselskiy (Kyiv Regional Clinical Hospital).

United Kingdom: Eilidh Bruce, Bing Lun Chow, Emma Iddles, Sarah McGuckin, Nicola Newall, George Ramsay, Parivrudh Sharma, Caitlin Stewart, Jeremy Wong (Aberdeen Royal Infirmary); Abdul Badran, Michael Bath*, Fanny Belais, Eman Butt, Kaustuv Joshi, Milan Kapur, Mike Shaw, Adam Townson, Christopher Yee Khang Williams (Addenbrooke’s Hospital); Timothy Gray, Robert Greig, Mansoor Husain, Elspeth Murray, Ahmed Mustafa (Borders General Hospital); Ashar Asif, Arya Gokul, Max Shah (Bristol Royal Infirmary); Mabel Temisanren Akitikori, Alexandros Charalabopoulos (Broomfield Hospital); Sophie Davidson, Sinead McNally, Shamil Rupani (Causeway Hospital); Fatema Juma, Sarah Catherine Mills, Laura Muirhead, Kate Sellars, Una Walsh, Oliver Warren (Chelsea and Westminster Hospital); Alice Chambers, Richard Hunt, Ella Teasdale (Cheltenham General Hospital); Stephen Boyce, Hannah Cornwall, Isabel Tol (Churchill Hospital); Eleftherios Orestis Argyriou, Nicola Eardley, Meical Povey (Countess of Chester Hospital); Joanna M S Aithie, Ahmer Irfan, Mari-Claire McGuigan, Robert Starr, Craig Russell Warren (Gartnavel General Hospital); Jess Archibald, Georgia Kirby, Ivan Kisyov (Gloucestershire Royal Hospital); Chun Kheng Khoo, Rachel Lee, Dana Photiou (Grantham and District Hospital); Rowan Davis, Uday Prasad, P Zichu Yang (Hairmyres Hospital); Jonathan Bird, Edmund Leung, Virginia Summerour (Hereford County Hospital); Chelise Currow, Jianshen Kiam, Gerald Jack Soon Tan (Ipswich Hospital); Anitha Muthusami, Ibifunke Pegba-Otemolu, Tomas Urbonas (John Radcliffe Hospital); Joseph Nunoo-Mensah, Edgaras Smolskas (King’s College Hospital); Alex Boddy, Gianpiero Gravante, David Hunter (Leicester Royal Infirmary); David Andrew, Amanda Koh, Amari Thompson (Lincoln County Hospital); Lawrence Adams, Hollie A Clements, Kasun De Silva, Ogbonnia Ekpete, Seraj Haque, Scott Henderson, Bilal Ibrahim, Thummini Jayasinghe, Jennifer Livie, Keir Mailley, Gopikrishnan Nair, Daniel Tan (Ninewells Hospital); Caitlin Baggaley, Aleksander Dawidziuk, Bartosz Szyszka (Northwick Park Hospital); Charlotte Barter, Nirav Gandhi, Karen Hassell, Samantha Hitchin, Jennett Kelsall, Eva Nagy, Ashrafun Nessa, Lisa Whisker, Fady Yanni (Nottingham City Hospital); Mahmoud Ali, Deeksha Arora, Sunanda Hediwattege, Navam Kumarasinghe, Munir Rathore, Athula Tennakoon (Pilgrim Hospital); Syed Mustafa Ali Ahmad, Oreoluwa Bajomo, Fahema Nadira (Princess Alexandra Hospital); Valerio Celentano (Queen Alexandra Hospital); Aneel Bhangu, James Glasbey, Ewen Griffiths, Rama Santhosh Karri, Jason Kei Chak Mak, Dmitri Nepogodiev, Michelle Pipe (Queen Elizabeth Hospital Birmingham); Muhammad Iqbal Bhatti, Mohamed Rabie (Queen Elizabeth Hospital King’s Lynn); Connor Boyle, David Hamilton, Aishath Mihuna, James Chean Khun Ng, Gary Nicholson, Agata Oliwa, Robert Pearson, Anna Rose, Shun Qi Yong (Queen Elizabeth University Hospital); Catherine Boereboom, Michael Hanna, Catherine Walter (Queens Medical Centre); Thomas Samuel Greensmith, Rachel Mitchell, Eimear Monaghan (Raigmore Hospital Inverness); James Crawford, Susan Moug (Royal Alexandra Hospital); James Blackwell, Hannah Boyd-Carson, Philip Herrod (Royal Derby Hospital); Omar Al-Allaf, Miriam Beattie, Cameron Bullock, Shivang Burman, Gemma Clark, Nicolas Flamey, Oliver Flannery, Alexander Harding, Ben Kodiatt, Samuel Lawday*, Shivani Mahapatra, Navin Mukundu Nagesh, Michael Ng, Dupinderjit Rye, Andrel Yoong (Royal Devon and Exeter Hospital); Laura Clark, Chris Deans, Monisha Edirisooriya, Cameron Fairfield, Ewen M Harrison (Royal Infirmary of Edinburgh); Emma Victoria Carrington, Tsz Lun Ernest Wong, Baasil Yusuf (Royal London Hospital); Carla Chamberlain, Kathryn Duke, Elizabeth Kmiotek (Royal Surrey County Hospital); Azel Botes, Natalie Condie*, Timothy Schrire, Reena Shah, Iolo Thomas-Jones, Charlotte Yates (Southmead Hospital); Natasha Anthony, Edward Matthews, Kapil Sahnan, James Tankel, Sally Tucker, Jasmine Winter Beatty, Paul Ziprin (St Mary’s Hospital); William Duggan, Anastasia Kantartzi, Shruthi Sridhar (St Thomas’ Hospital); Rachel Alys Khaw, Prakhar Srivastava, Charlotte Underwood (The Christie Hospital); Homero Alves do Canto Brum, Sharat Chopra, Laura Davis (University Hospital of Wales); Rebecca Hughes, Joshua Tulley (Walsall Manor Hospital); Justin Alberts, Thomas Athisayaraj, Mojolaoluwa Olugbemi (West Suffolk Hospital); Kasim Ahmad, Claudia Chan, Gavin Chapman, Hannah Fleming, Benjamin Fox, Julia Grewar, Kate Hulse, Duncan Rutherford, Mackay Sinead, Scott Smith, Doug Speake*, Peter G Vaughan-Shaw (Western General Hospital); Natasha Christodoulides, Simrit Kudhail, Matthew Welch (Wexham Park Hospital); Syed Muhibullah Husaini, Simon Lambracos (Worthing Hospital).

United States: Chikamuche Anyanwu, Rishi Suresh, Jimmy Scott Thomas (Baylor Scott&White Medical Center); Elizabeth Gleeson, Rebecca Platoff, Areeba Saif (Hahnemann University Hospital); Zachary Enumah, Eric Etchill, Alodia Gabre-Kidan (Johns Hopkins Hospital); Mitchell Bernstein, Francesco Maria Carrano, Joseph Connors, Patricio Lynn, Marcovalerio Melis, Elliot Newman (NYU Langone Medical Center); Deshka S Foster, Kenneth Perrone, Ashley Titan, Thomas G Weiser (Stanford Health Care); Sarwat Ahmad, Andrea Chao M. D. Bafford, Marco Dal Molin, Nader Hanna, Syed Nabeel Zafar (University of Maryland Hospital); Mark Hemmila, Lena Napolitano, Jane J Wong (University Of Michigan Medical Center); Julia Chandler, Lauren Wood, Sherry Wren (VA Palo Alto Hospital); Taylor Ottesen, Lucia You, Kristin Yu (Yale New Haven Hospital).

Uruguay: María del pilar Arciénega Yañez, Martin Ferreira Fernandes, Daniel González (Cooperativa Medica de Florida); Santiago Cubas, María Catalina González, Vanessa Zubiaurre (Hospital De Clinicas); Rodrigo Demolin, Nicolas Giroff, Pablo Sciuto (Hospital Espanol); Maite Campos, Gabriela Rodríguez Cantera, Gabriela Wagner (Hospital Maciel).

Zambia: Garg Deepika, Mayaba Maimbo, Elliot Simuchimba (Kitwe Teaching Hospital); Anadi Bulaya, Chali Chibuye, Bright Chirengendure (Ndola Central Hospital); Mary-Rose Kabale, Kizito Kabongo, David Linyama, James Munthali, Oliver Mweso, Francis Pikiti (University Teaching Hospital).

#### Data Validators

Australia: James Otieno (Calvary Mater Newcastle); Erick Chan (Gold Coast University Hospital); Log Tung Lai (Gosford Hospital); Brighid Blackman (Gosford Private Hospital); Sophie Richards (John Hunter Hospital); Suren Subramaniam (Peter MacCallum Cancer Centre); Rafid Karim (Princess Alexandra Hospital); Nathan Kok (Redcliffe Hospital); Yanni Dion Lee (Royal Adelaide Hospital); Shabina Ali (The Queen Elizabeth Hospital); Aanjaneya Sinha (The Wesley Hospital); Robert Corrigan (Toowoomba Hospital); Nicole Barnes (University Hospital Geelong); Florence Wong (Westmead Hospital); Grace Dennis (Wyong Public Hospital).

Austria: Julia Jedamzik (General Hospital of Vienna).

Barbados: Emil Phillips (Queen Elizabeth Hospital).

Belgium: Wivine Piette (Grand Hopital de Charleroi - Site Saint-Joseph); Marie Van hentenryck (Hôpitaux Iris Sud - Etterbeek-Ixelles).

Benin: Houenoukpo Koco (Centre Hospitalier Universitaire et Departemental Oueme Plateau); Souliath Lawani (Centre National Hospitalier et Universitaire Hubert Koutoukou Maga).

Botswana: Mamo Woldu Kassa (Princess Marina Hospital).

Brazil: Tainá Santos Bezerra (Hospital Santa Casa de Misericordia de Maceio).

Bulgaria: Petar Gribnev (University Hospital Alexandrovska); Dobromir Dimitrov (University Hospital Dr Georgi Stranski); Panche Krastev (University Hospital Eurohospital).

Cambodia: Sovannarith Oum (World Mate Emergency Hospital).

Cameroon: Divine Tim Bonghaseh (Baptist Hospital).

Canada: Maryam Al Farsi (Jewish General Hospital); Nourah Alsharqawi (McGill University Health Center); Arnav Agarwal (Sunnybrook Hospital).

Colombia: Veronica Acevedo (CES Clinic); Andrea Carolina Castillo Barbosa (Fundacion Cardioinfantil-IC); Felipe Giron (Hospital Universitario Mayor Méderi); Jimmy Paul Leon Rodriguez (Hospital Universitario San Vicente Fundacion).

Croatia: Darko Kučan (Clinical Hospital Merkur); Damir Rosko (General Hospital Dr. Josip Bencevic); Neven Barsic (University Hospital Center Sestre milosrdnice); Domagoj Župan (Zadar General Hospital).

Czech Republic: Amgad Hegazi (Charles University Hospital); Vendula Trunčíková (Slezská nemocnice v Opavě, p.o.); Vladimir Fryba (The General University Hospital in Prague).

Egypt: Mostafa Mohamed (Ain Shams University Specialized Hospital); Ahmed Sultan (Al-Tagamoh Hospital); Ahmed Nagi (Alexandria Main University Hospital); Abdallah Rashad Temerik (Assiut University Hospital); Mohamed Elemam Elshawy (El Demerdash University Hospital); Moustafa Ibrahim Mahmoud (Gamal Abd El Nasser Hospital); Shrouk Omar (Kasr Alainy Hospital, Faculty of Medicine, Cairo University); Mohamed Anwar (Mansoura University Hospital); Tarek Rageh (Menofiya University Hospital); Aya Elmokadem (National Cancer Institute); Khaled Gaballa (Oncology Center Mansoura University).

Estonia: Sandra Teppo (The North Estonia Medical Centre).

Finland: Antti Turunen (Helsinki University Hospital); Pasi Pengermä (Kanta-Hame Central Hospital).

France: Quentin Ballouhey (CHU Limoges); Damien Bergeat (CHU Rennes - General Surgery); Ariane Weyl (CHU Toulouse); Elisabeth Hain (Hôpital Cochin - APHP).

Ghana: Adam Gyedu (Komfo-Anokye Teaching Hospital); Edwin Yenli (St. Patrick’s Hospital); Dorcas Osei-Poku (Tamale Teaching Hospital).

Greece: Vaia-Aliki Rompou (Agios Savvas Anticancer Hospital); Athanasios Zoikas (Athens Medical Center); Apostolos Gaitanidis (Attikon University General Hospital); Georgios Koukis (General Hospital of Nikaia); Konstantinos Perivoliotis (General University Hospital of Larissa); Panagiotis Tavlas (General University Hospital of Patras); Konstantinos Galanos-Demiris (George Papanikolaou General Hospital of Thessaloniki); George Zografos (Hippocratio General Hospital); Ioannis Karavokyros (Laiko University Hospital); Georgia Xanthopoulou (Naval And Veterans Hospital); Eirini Iordanidou (Serres General Hospital).

Guatemala: Fernanda Ayau (Hospital General De Enfermedades); Allan Garcia (Hospital General San Juan De Dios).

Hungary: Pekli Damján (1st Department of Surgery - Semmelweis University).

India: Deepender Wason (Pandit Bhagwat Dayal Sharma Post Graduate Institute of Medical Sciences); Ashika B L (Victoria Hospital).

Indonesia: Ervandy Rangganata (Dr Cipto Mangunkusumo National General Hospital).

Ireland: Prerna Kamath (St. James’s Hospital); Donal B O’Connor (Tallaght Hospital).

Italy: Margherita Pinto (Azienda Ospedaliera Regionale ‘San Carlo’); Fabrizio Perrone (Azienda Ospedaliero Universitaria Consorziale Policlinico Di Bari); Francesca Paola Tropeano (Federico II University of Naples); Francesca Troilo (Hospital G.Bernabeo); Daniela Bossi (ICS Maugeri); Dario Scala (Istituto Nazionale Tumori Fondazione, Pascale-I.R.C.C.S.); Lucrezia Pulitanò (Mater Domini University Hospital); Marcella Carella (Ospedale Generale Regionale F. Miulli); Andrea Pietrabissa (Policlinico San Matteo); Alice Gori (S.Orsola-Malpighi Hospital); Giorgio Giraudo (Santa Croce and Carle Hospital); Veronica De Simone (Santa Rita Clinic, Vercelli); Alfio Alessandro Russo (Treviglio Hospital); Bartolomeo Braccio (Universitá della Campania ‘Luigi Vanvitelli’, Naples).

Jordan: Raed Al-Taher (Jordan University Hospital); Sarah Athamneh (King Abdullah University Hospital).

Kenya: Andrea Parker (Tenwek Hospital).

Libya: Adnan Sawiee (Alkhadra Hospital); Amina Kattia (Misurata Cancer Center); Malik Salem (National cancer institute); Osama Tababa (New Bridge Hospital); Zuhour Shaeeb (Tripoli Central Hospital).

Lithuania: Vilius Syminas (Klaipeda Republic Hospital); Jonas Jurgaitis (Klaipeda University Hospital); Gytė Damulevičienė (Lithuanian University of Health Sciences Kaunas Clinical Hospital); Saulius Svagzdys (Lithuanian University of Health Sciences Kaunas Clinics); Tomas Poskus (Vilnius University Hospital).

Madagascar: Narindra Njarasoa Mihaja Razafimanjato (Joseph Ravoahangy Andrianavalona Hospital).

Malaysia: Ling Chieng Loo (Hospital Sibu); Ing Ching Tiong (Hospital Sultanah Bahiyah); Wan Farahiyah Wan Muhmad (Hospital Tuanku Ja’afar); Harinthiran Vijeyan (Hospital Universiti Sains Malaysia); Teoh Li Ying (University Malaya Medical Centre).

Malta: Gabriella Grech (Mater Dei Hospital).

Mexico: Rodrigo Arrangoiz (ABC Medical Center); Vania Brickelia Jimenez Ley (ANKER Oncologia Global Especializada); Daniel Arizpe (Hospital Angeles Puebla); Vania Brickelia Jimenez Ley (Hospital de Especialidades, CMNO-IMSS); Elizabeth Lagunes Lara (Hospital Español Veracruz); Elizabeth Victoria Castro López (Hospital Regional de Alta Especialidad); Jose Eaazim (Instituto Nacional de Ciencias Médicas y Nutrición ‘Salvador Zubirán’).

Netherlands: Marije Gordinou de Gouberville (Amsterdam UMC VUmc); Vivian Bastiaenen (Amsterdam UMC, University of Amsterdam); Simone Rottier (Tergooi Hospital).

New Zealand: Fouad Nahab (North Shore Hospital); Maria Yeonhee Ji (Taranaki Base Hospital).

Nigeria: Mohammed Seyoji (Ahmadu Bello University Teaching Hospital); Callistus Nwachukwu (Aminu Kano Teaching Hospital); Okechukwu Emeghara (Babcock University Teaching Hospital); Sayyid Egbunu Muhammed (Barau Dikko Teaching Hospital); Ayodeji Idowu (Ekiti State University Teaching Hospital); Olamiposi Sowemimo (Obafemi Awolowo University Teaching Hospitals Complex); Olakayode Ogundoyin (University College Hospital); Oluwatosin Akande (University of Ilorin Teaching Hospital).

Norway: Alexander Lott (University Hospital Of North Norway).

Pakistan: Maliha Nadeem (Ganga Ram Hospital); Ahsan Ali Laghari (Liaquat University of Medical & Health Sciences); Asif Loya (Shaukat Khanam Memorial Cancer Hospital and Research Centre); Hassan Mushtaq (Sindh Institute of Urology and Transplantation); Muhammad Tariq Abdullah (The Pakistan Institute of Medical Sciences).

Palestine: Baseel Abuhilal (Al Makassed Islamic Charitable Society Hospital Jerusalem); Mohammad Atawneh (Al-Ahli Hospital); Hamdan Hamdan (Beit Jala Governmental Hospital (Al Hussein)); Belal Alhabil (Indonesian Hospital); Abedelrahman Srour (Palestine Medical Complex); Ibrahim Mousa (Rafidia Hospital).

Paraguay: Luis Da Silva Medina (Hospital de Clínicas, II Cátedra de Clínica Quirúrgica, Universidad Nacional de Asunción).

Philippines: Marie Dione Sacdalan (José R. Reyes Memorial Medical Center); Marie Carmela Lapitan (Philippine General Hospital, University Of The Philippines Manila); Marie Dione Sacdalan (Quirino Memorial Medical Center); Marie Dione Sacdalan (The Medical City).

Poland: Katarzyna Bartosiak (Military Institute Of Medicine).

Portugal: Pedro Ferreira (Centro Hospitalar de Setúbal); Vítor Francisco (Centro Hospitalar do Baixo Vouga); Ricardo Lemos (Centro Hospitalar do Medio Ave); Luísa Frutuoso (Centro Hospitalar Entre o Douro e Vouga); Sara Fernandes (Centro Hospitalar Lisboa Norte); Telma Fonseca (Centro Hospitalar Sao Joao); Jorge Pereira (Centro Hospitalar Tondela-Viseu); Juan Rachadell (Centro Hospitalar Universitario do Algarve); Ana Torre (Centro Hospitalar Vila Nova de Gaia/Espinho); Filipe Madeira Martins (Hospital da Horta, E.P.E.); Ana Cristina Carvalho (Hospital da Senhora da Oliveira); Joana Rodrigues Ferreira (Hospital de Egas Moniz); Bruno Ribeiro da Silva (Hospital de Santa Luzia); Helena Devesa (Hospital de Santarem); Ana Vieira (Hospital de Santo Espirito da ilha Terceira); Inês Mónica (Hospital Distrital da Figueira da Foz); Margarida Amaro (Hospital do Espirito Santo); Diogo Sousa (Hospital do Litoral Alentejano); Marta Reia (Hospital Doutor José Maria Grande); João Louro (Hospital Geral - Centro Hospitalar de Coimbra); Ana Martins (Hospital Prof. Doutor Fernando Fonseca, E.P.E.); Joaquina Dominguez (Hospital Santa Luzia Elvas); Inês Santos (Hospital Sao Francisco Xavier); Nuno Miguel Freitas Oliveira (Hospital Vila Franca de Xira); José Carlos Pereira (IPO Porto); Pedro Silva-Vaz (Unidade Local de Saúde de Castelo Branco); Ligia Freire (Unidade Local de Saude de Matosinhos - Hospital Pedro Hispano); Ricardo Escrevente (Unidade Local de Saude do Baixo Alentejo).

Romania: Valentina Madalina Negoita (Emergency Clinical Hospital Bucharest).

Russian Federation: Dmitry Shakhmatov (State Scientific Centre of Coloproctology).

Rwanda: Yves Nezerwa (Rwanda Military Hospital).

Serbia: Radosav Radulovic (Zvezdara University Medical Center).

South Africa: Rachel Moore (Chris Hani Baragwanath Academic Hospital); Gareth Obery (Groote Schuur Hospital); Francois Viljoen (Inkosi Albert Luthuli Central Hospital); Tome Mendes (Mitchell’s Plain District Hospital).

Spain: Antonio Suarez (12 de Octubre University Hospital); Enrique Moncada (Álvaro Cunqueiro Hospital); Maria Fernandez-Hevia (Central University Hospital Of Asturias); Carolina Curtis Martínez (Hospital General Universitario de Elche); Julia Maria Gil Garcia (Hospital Universitari de Girona Dr. Josep Trueta); Mariana González Zunzarren (Infanta Sofía University Hospital).

Sudan: Tarig Idris (Ibrahim Malik Teaching Hospital).

Sweden: Karolina Eklöv (South General Hospital); Oskar Grahn, Leila Amin (Umea University Hospital); Malin Blomqvist (Vrinnevi Hospital).

Switzerland: Costanza Ajani (Ente Ospedaliero Cantonale); Rebecca Kraus (Kantonsspital Graubunden); Nico Seeger (Kantonsspital Winterthur); Melissa Willemin (Lausanne University Hospital CHUV).

Syrian Arab Republic: Fadi Rayya (Al-Assad University Hospital); Mohammad Ayash (Al-Bairouni University Hospital); Raneem Msouti (Al-Mouwasat University Hospital); Israa Kannas (Aleppo University Hospital); Eias Abazid (Damascus Hospital); Asil Esper (Tishreen University Hospital).

Tunisia: Skander Slim (Institut Salah Azaiez).

Turkey: Akil Serdar Kavcar (Acibadem Atakent Hospital); Erman Aytac (Acibadem Maslak Hospital); Ahmet Cem Dural (Bakirkoy Dr. Sadi Konuk Training And Research Hospital); Ayse Ilker (Cerrahpasa Medical Faculty Istanbul University); Ismail Cem Eray (Cukurova University Faculty of Medicine); Eray Kurnaz (Erzincan University Hospital); Saygin Altiner (Gazi University Medical Faculty Hospital); Mustafa Deniz Tepe (Karadeniz Technical University Farabi Hospital); Can Şahin (Memorial Ankara Hospital); Evrim Savli (Samsun Training and Research Hospital).

Uganda: Aryon Innocent (Fort Portal Regional Referral Hospital); Lilian Babirye (Kampala International University Teaching Hospital).

Ukraine: Andrii Diachenko (Dnepropetrovsk Regional Clinical Oncology Center); Vladislav Hordoskiy (Kyiv Regional Clinical Hospital).

United Kingdom: Heather Curry (Aberdeen Royal Infirmary); Charlene Yat Che Chau (Addenbrooke’s Hospital); Harry Robertson (Bristol Royal Infirmary); Arin Mahmoud (Broomfield Hospital); Hannah Lennon (Countess of Chester Hospital); Lynette Loi (Gartnavel General Hospital); Emily Kirkham (Gloucestershire Royal Hospital); Cameron McCann (Hairmyres Hospital); Daniel Watts (John Radcliffe Hospital); Binay Gurung (Leicester Royal Infirmary); Michael Wilson (Ninewells Hospital); Thomas Tribedi (Nottingham City Hospital); Eleonora Garofalo (Queen Alexandra Hospital); Baryab Zahra (Queen Elizabeth University Hospital); Scott MacDonald (Royal Alexandra Hospital); Ian Daniels (Royal Devon and Exeter Hospital); Nathan Ng (Royal Infirmary of Edinburgh); Shivun Khosla (Royal Surrey County Hospital); James Olivier (Southmead Hospital); Sum Yu Pansy Yue (St Thomas’ Hospital); Gayathri Suresh (The Christie Hospital); Jack Wellington (University Hospital of Wales); Emmanuel Lorejo (West Suffolk Hospital); Mafdi Mossaad (Wexham Park Hospital); Yegor Tryliskyy (Worthing Hospital).

United States: Madison Crutcher (Hahnemann University Hospital); Marjan Alimi (NYU Langone Medical Center); Ioana Baiu (Stanford Health Care); Hossam Abdou (University of Maryland Hospital); Alison Conway (VA Palo Alto Hospital); Connor Peck (Yale New Haven Hospital).

Uruguay: Gabriela Wagner (Hospital De Clinicas); Mauro Andres Perdomo Perez (Hospital Espanol); Ivan Trostchansky (Hospital Maciel).

Zambia: Stanley Zulu (Kitwe Teaching Hospital); Mildred Nakazwe (University Teaching Hospital).

### APOLLO

#### Study Management Group

William Xu, Chris Varghese, Daoud Chaudhry, Mustafa Ege Seker, Noor Essa, Mafalda Sampaio Alves, Moritz Steinruecke, Setthasorn Ooi, Adam Turňa, Irene Santos, Laura Kehoe, Muhammad Elhadi.

#### Expert advisory group

Ian Bissett, Stephen Chapman, Ruth Blanco Colino, James Glasbey, Susan Moug, Dion Morton, Wal Baraza, Sue Blackwell, Dimitri Nepogodiev, Francesco Pata, Gianluca Pellino, Peter Pockney, Alessandro Sgrò.

#### National Leads

Amanda Dawson, Loranne Gaborit, Sarah Goh (Australia), Samir Delibegovic (Bosnia and Herzegovina), Arwa Mohamad (Bulgaria), Jakov Mihanovic (Croatia), Daniela Arbeláez-Lelion (Colombia), Daniela Merz (Germany), OrestisIoannidis, Argyrios Ioannidis (Greece), Sahana Bopparaju (Telangana-India), Francesco Pata, Gianluca Pellino (Italy), Hoshika Tharni Sivapalan (Latvia), Muhammed Elhadi (Libya and MENA), Albertas Dauksa (Lithuania), Andee Dzulkarnaen Zakaria (Malaysia), Luis Adrian Alvarez-Lozada (Mexico), Anthony Lin, Shuba Kosna (New Zealand), Ademola Adeyeye (Nigeria), Umar Saeed (Pakistan), Irène Santos, José Guilherme Gonçalves-Nobre, Mafalda Sampaio-Alves (Portugal), Anastasia Novikova (Russian Federation), Melik Kağan Aktaş, Mustafa Deniz Tepe (Türkiye), Jurij Ales Kosir (Slovenia), Neoleen Van Staden (South Africa),Ruth Blanco-Colino (Spain),Cristiana Riboni, Dimitri Christoforidis (Switzerland), Moritz Steinruecke, Setthasorn Ooi, Louise Ko (United Kingdom).

#### Regional Leads

Kirsty Luo-Yng Tay (Aberdeen); Fatimah Khan (Anglia Ruskin University); Ronald Hang Kin Nam (Aston); Aleksandra Lopuszko (Barts and the London (QMUL)); Patrick Keane (Belfast (QUB)); Warda Jamshaid (Birmingham ); Pierre Jean-Marie (Brighton & Sussex); Runqing Su (Bristol); Vian Omar (Buckingham); Ellen Fung (Cambridge); Maryam Jamshaid (Cardiff); Ashna Ashpak (Central Lancashire); Xianghan Zheng (Cork); Ned Quirke (Dublin (UCD)); Tasnim Kouli (Dundee); Yuk Wing Liza Chong (Edinburgh); Alexandra Sebastiao (Exeter); Nahl Iftikhar (Glasgow); Mitchel Shula (Hull York ); Kavyesh Vivek (Imperial); Balamrit Singh Sokhal (Keele); Anthony Siu (Kings); Hamzah Ahmin (Lancaster); Ankit Gupta (Leeds); Sanjana Shaunak (Leicester); Arooj Qaiser (Lincoln (Nottingham)); Shubhi Gupta (Liverpool); Ayesha Qureshi (Manchester); Richard Huynh (Newcastle); Rhea Suribhatla (Oxford); Sam Ghaznavi (Peninsula (Plymouth)); Sanjana Mehrotra (Sheffield); Kiran Stowell (Southampton); Kofi Cox (St George's); Hermes Manos (St. Andrews); Roshni Patel (Swansea); K.Ewomazino Oderoha (Trinity College Dublin); Raian Jaibaji (UCL); Ellisa Baggott (UEA (Norwich)); Semhar Abraha (Warwick), Cristiana Riboni, Salomone di Saverio, Alice Gori, Vinicio Mosca, Mauro Podda, Matteo Rottoli, Alessandro Sgrò (Italy).

#### Hospital Leads

Algeria: Anisse Tidjane (Ehu-1st November 1954).

Australia: David Proud (Austin Hospital); Shane Zhang (Calvary Mater Newcastle); Gemma Qian, Amanda Dawson(Gosford Hospital); Edward Zhang (John Hunter Hospital); Antonio Barbaro (Lyell McEwin Hospital); Madison Lowe (Mount Gambier And Districts Health Service); Madhavi-Priya Singh (Northern Hospital); Luke Traeger, Tarik Sammour (Royal Adelaide Hospital); Talia Shepherd (Royal Perth Hospital); Mary Theophilus (St John Of God Midland Public And Private Hospital); Qiwen Wang (The Queen Elizabeth Hospital); Richard G McGee (Wyong Public Hospital).

Bosnia And Herzegovina: Merima Kruščica, Mirhan Salibašić (Clinical Center University Of Sarajevo); Haris Kuralić (University Clinical Center Tuzla).

Bulgaria: Dimitar Hadzhiev (Umhat Sveti Georgi); Martin Karamanliev (University Hospital Dr Georgi Stranski).

Colombia: Daniela Arbelaez Lelion (Hospital Pablo Tobón Uribe).

Croatia: Jakov Mihanovic (Zadar General Hospital).

Egypt: Ahmed Sabry (Alexandria Main University Hospital); Ahmed M. Abbas (Assiut University Hospital); Sarah Abdelmohsen (Aswan University Hospital); Ahmed Saber Mohamed Abdelrahman (Giza International Hospital); Khaled Abdelwahab (Oncology Center Mansoura University); Mohamed Elbahnasawy (Tanta University Hospital); Bassam Fahmy (The Memorial Soaad Kafafi University Hospital).

Germany: Daniel Reim (Technical University of Munich, School of Medicine and Health, TUM University Hospital, Department of Surgery); Gregor Massoth (University Hospital Bonn); Ulrich Ronellenfitsch (University Hospital Halle).

Greece: Dimitrios Korkolis (Agios Savvas Anticancer Hospital); Christos Chouliaras (Athens Medical Center); Dimitrios Manatakis (Athens Naval And Veterans Hospital); Theodoros A Sidiropoulos (Attikon University General Hospital); Maria Sotiropoulou (Evaggelismos General Hospital); Prokopis Christodoulou (General Hospital Asklepieio Voulas); Francesk Mulita (General University Hospital Of Patras); Orestis Ioannidis (George Papanikolaou General Hospital Of Thessaloniki); Maximos Frountzas (Hippocratio General Hospital); Nikolaos Machairas (Laiko University Hospital); Ioannis Katsaros (Metaxa Cancer Hospital); Michael Spartalis (Sotiria General Hospital Of Thoracic Diseases); Konstantinos Lasithiotakis (University Hospital Of Heraklion Crete).

India: Yashwant Sakaray (Post Graduate Institute of Medical Education and Research).

Iraq: Ali Kadhim (Al-hussien Medical City).

Ireland: Ireland: Ned Quirke (St Vincent's University Hospital); Kevin Ewomazino Oderoha (St James's Hospital).

Italy: Stefano Piero Bernardo Cioffi (Asst Grande Ospedale Metropolitano Niguarda); Bruno Nardo (Azienda Ospedaliera Di Cosenza); Nicola Passuello (Azienda Ospedaliera Di Padova); Giulia Turri (Azienda Ospedaliera Universitaria Integrata Di Verona); Massimiliano Veroux (Azienda Ospedaliero- Universitaria Policlinico San Marco); Mauro Podda (Cagliari University Hospital); Alan Biloslavo (Cattinara University Hospital); Alessandra Marano (A.O.U. Città della Salute e della Scienza di Torino); Marco Amisano (Irccs Ospedale Policlinico San Martino); Daniela Rega (Istituto Nazionale Tumori Fondazione); Pasquale Cianci (Lorenzo Bonomo); Luca Cardinali (Madonna Del Soccorso Hospital); Daunia Verdi (Mirano Hospital); Fabrizio D'acapito (Morgagni-Pierantoni Hospital AUSL Romagna); Alessandro Broglia (Ospedale Civile Di Voghera); Francesco Maria Carrano (Ospedale Di Circolo Di Busto Arsizio); Giovanni Tarchi (Ospedale Di Legnano); Marco De Prizio (Ospedale San Donato Usl Toscana Sud Est); Nicolò Tamini (IRCCS San Gerardo dei Tintori - Monza); Andrea-Pierre Luzzi (Ospedale Villa Scassi); Vincenzo Lizzi (Ospedali Riuniti Azienda Ospedaliera Universitaria Foggia); Pierfrancesco Lapolla (Policlinico Umberto I); Francesco Fleres (University of Messina, Messina); Gaetano Poillucci (San Matteo Degli Infermi); Marco Clementi (San Salvatore Hospital, University of L’Aquila); Valeria Tonini (Santa Annunziata Hospital); Giacomo Calini (Ospedale Santa Maria della Misericordia di Udine); Nicola Cillara (Santissima Trinità - Ats Sardegna); Matteo Desio (University Of Insubria).

Jordan: Majedah Hmeidan (Al-Tafilah New Hospital); Bourhan Alrayes (Islamic Hospital); Almu'atasim Khamees (Jordan University Hospital); Mahmoud Mahafdah (King Abdullah University Hospital/ Jordan University Of Science And Technology); Samah Alananzeh (Princess Basma Hospital).

Latvia: Nityanand Jain (Pauls Stradins Clinical University Hospital)

Libya: Arwa Kara (Benghazi Medical Center); Hamida El Magrahi (Crown Health Care Clinical Team); Hibah Bileid Bakeer (Gharyan Central Hospital); Ayyah Emran (Tobruk Medical Center); Eman Abdulwahed (Tripoli Central Hospital); Mohamed Alsori (Tripoli Medical Center/ Tripoli University Hospital); Najat Ben Hasan (Zliten Teaching Hospital).

Lithuania: Kristina Marcinkevičiūtė (Vilnius University Hospital Santaros Klinikos).

Malaysia: Andee Dzulkarnaen Zakaria (School of Medical Sciences & Universiti Sains Malaysia Specialist Hospital); April Camilla Roslani (University Malaya Medical Centre).

Mexico: Francisco Emmanuel Alvarez Bautista (Hospital General Dr. Manuel Gea González); Danilo Tueme De La Peña (Instituto Nacional De Ciencias Médicas Y Nutrición ‘Salvador Zubirán’).

Morocco: Samia Errami (Hopital Ibn Tofail); Samia Kessab (Institut National D'oncologie).

New Zealand: Ashley Pereira, Wal Baraza (Auckland City Hospital); William Ju, Tamara Glyn (Christchurch Hospital); Avinash Sathiyaseelan, Sze Lin Peng (Middlemore Hospital); Cheuk Lam Jeffrey Lui, Xiao Shen Hu, Siraj Rajaratnam (North Shore Hospital); Edmund Leung (Taranaki Base Hospital); Binura Lekamalage, Jeremy Rossaak (Tauranga Hospital); Niki Kau, Jesse Fischer (Waikato Hospital); Sarah Rennie (Wairarapa Hospital); Mairarangi Haimona, Anthony Lin (Wellington Regional Hospital); Matthew McGuinness, Christopher Harmston (Whangarei Hospital).

Nigeria: Abubakar Bala Muhammad (Aminu Kano Teaching Hospital); Nurudeen Akinbami (University College Hospital); Matthew Bojuwoye (University Of Ilorin Teaching Hospital).

Pakistan: Warda Ahmed (Aga Khan University).

Poland: Jan Nicikowski (University Hospital of Karol Marcinkowski in Zielona Góra).

Portugal: Sofia Reis (Centro Hospitalar Barreiro Montijo); Daniela Martins (Centro Hospitalar De Trás-Os-Montes E Alto Douro); Marta Costa (Centro Hospitalar E Universitário De Coimbra - Hospital Geral); Penélope Correia (Centro Hospitalar Entre O Douro E Vouga); Rita Galama (Centro Hospitalar Médio Tejo); Beatriz Mendes (Centro Hospitalar Universitario Do Algarve - Unidade De Portimão); Ana Rita Loureiro (Hospital Das Caldas Da Rainha - Centro Hospitalar Do Oeste); Sonia Fortuna Martins (Hospital De Santarem); Joana Bolota (Hospital Do Espirito Santo); Alberto Silva (Hospital Do Litoral Alentejano); Madalena Trindade (Hospital Garcia De Orta); Lígia Freire (Unidade Local De Saude De Matosinhos - Hospital Pedro Hispano).

Qatar: Leena Aboidris (Hamad General Hospital).

Russian Federation: Sergey Efetov (Im Sechenov First Moscow State Medical University); Aleksandr Butyrskii (Municipal Emegency Hospital No.6); Alexey Yanishev (Privolzhsky Research Medical University).

South Africa: Margot Flint (Groote Schuur Hospital).

Spain: Zutoia Balciscueta (Hospital Arnau De Vilanova); David Moro-Valdezate (Hospital Clínico Universitario De Valencia); Ana Maria Minaya Bravo (Hospital Del Henares); Silvia Pérez-Ajates (Hospital General Universitario Gregorio Marañón); Fernando Mendoza-Moreno (Hospital Universitario Principe De Asturias); Noelia Ibáñez (Hospital Universitario Virgen De La Arrixaca); Felipe Pareja Ciuró (Hospital Universitario Virgen Del Rocio); Jorge Sancho-Muriel (Hospital Universitario Y Politécnico La Fe); Aitor Landaluce-Olavarria (Hospital Urduliz); Ana María Camacho Oliva (Puerta del Mar University Hospital); Mercedes Estaire Gómez (Severo Ochoa University Hospital).

Sri Lanka: Umesh Jayarajah (Colombo South Teaching Hospital); Sanjeewa Seneviratne (National Hospital Of Sri Lanka).

Sudan: Mohamed Musa Yassin (Gadarif Teaching Hospital); Essam Eldien Abuobaida (Ribat University Hospital).

Switzerland: Vaihere Delaune (Geneva University Hospitals); Joanna Naemi Marx (Kantonsspital Olten); Jörn-Markus Gass (Luzerner Kantonsspital)

Syrian Arab Republic: Mohamad Klib (Al-Mouwasat University Hospital); Anwar Chammout (Aleppo University Hospital).

Turkey: Gokalp Kagan Kurtoglu (Acibadem Altunizade Hospital); Emre Tuzuner (Acibadem Maslak Hospital); Beste Yıldırım (Dokuz Eylul Univ. Hospital); Kaan Okumuş (Ege University Hospital); Banu Yigit (Elazig Fethi Sekin City Hospital); Abdullah Emre Askin (Istanbul Medipol University Hospital); Ergin Erginöz (Istanbul Universty - Cerrahpaşa Medical Faculty); Ozgem Uysal (Izmir Katip Celebi University Faculty of Medicine); Aras Emre Canda (Acibadem Izmir Kent Hospital); Ayse Nilufer Yuzgec (Karadeniz Technical University Farabi Hospital); İbrahim Halil Özata (Koç University Medical School); Muhammed Enes Tasci (Marmara University School of Medicine); Nurhilal Kızıltoprak (Sultan 2. Abdülhamid Han Research and Training Hospital); Merve Yaren Kayabaş (Trakya University Hospital); Kemal Erdinç Kamer (University Of Health Sciences Tepecik Training And Research Hospital); Bulent Citgez (Uskudar University Faculty Of Medicine).

United Kingdom: Kirsty Luo-Yng Tay (Aberdeen Royal Infirmary); Alexia Farrugia (Sandwell General Hospital), Jessica Chang (Good Hope Hospital), Sharad Karandikar (Heartlands Hospital), Smaragda Gkolia (Royal London Hospital), Tara Chan-a-sue (Belfast City Hospital), Aine McGettigan (Royal Victoria Hospital), Ellen Dunlop (Ulster Hospital Dundonald), Grace Doherty (Antrim Area Hospital- Northern Health And Social Care Trust), Feargal McKey (Craigavon Area Hospital), Shabnam Cyclewala (Bristol Royal Infirmary), Vimaladhithan Mahendran (Yeovil District Hospital), Isabella Sawyer (Royal United Hospital Bath), Thomas Hibbs (Gloucestershire Royal Hospital), Hayden Simmons (Western General Hospital), Felicity Greenfield (Great Western Hospital), Pierre Jean-Marie (Royal Sussex County Hospital), Frances Dixon (Milton Keynes University Hospital), James Everson (Stoke Mandeville), Zafar Shahbaz (South Warwickshire NHS Foundation Trust), Fathima Manaal (Addenbrooke's Hospital), Kwan Wai Fung (Peterborough City Hospital), Jianing You (Hinchingbrooke Hospital), Cheuk Man Lam (West Suffolk Hospital), Emily Smith (Bedford Hospital), Ashna Ashpak (Royal Blackburn Hospital), Jenna Cook (Ninewells Hospital), Liza YW Chong (Western General Hospital), Melissa Bennett (Royal Devon And Exeter Hospital), Aris Alexiadis (Royal Cornwall Hospital), Hwei Jene Ng (Royal Alexandra Hospital), Mishal Shahid (Inverclyde Royal Hospital), Kavyesh Vivek (West Middlesex University Hospital), Kavyesh Vivek (Chelsea And Westminster Hospital), Kavyesh Vivek (St Mary's Hospital), Kavyesh Vivek (Charing Cross Hospital), Sadhasivam Ramasamy (University Hospitals Of North Midlands), Kareem Omran (Guy's And St Thomas' Hospitals), Ruairi Doherty (Furness General Hospital), Aqib Khan (Royal Lancaster Infirmary), Arooj Qaiser (Lincoln County Hospital), Jahnavi Kalvala (Queens Medical Centre), Michaela Silver (St James's University Hospital Leeds), Matthew Fok (Countess Of Chester Hospital), Rachael Clifford (Aintree University Hospital), Shubhi Gupta (Wirral University Teaching Hospital), Ammarah Ughratdar (Royal Preston Hospital), Oluwatobi Adegboye (Salford Royal Hospital), Yik Roy Hwang (Darlington Memorial Hospital), Khadija Khan (Gateshead Health NHS Foundation Trust), Reeves Campbell (Newcastle Upon Tyne Hospitals NHS Foundation Trust), Rhea Suribhatla (John Radcliffe Hospital), charlotte johnson (Derriford Hospital), Archchun Karunananthan (Derriford Hospital), Nujha Begum (Torbay And South Devon NHS Trust), Cody Breese (Scunthorpe General Hospital), Ahmad Gulzar (Barnsley Hospital NHS Foundation Trust), Emma Nofal (Rotherham District General Hospital), Rina George (Doncaster Royal Infirmary), Kiran Stowell (Dorset County Hospital), Kiran Stowell (Royal Hampshire County Hospital), Alex Glendenning (Morriston Hospital Swansea), Kofi Cox (St George's Hospital), Panagiotis Kapsampelis (Kingston), Peter Crabtree (University College London Hospital), Angus Gao (Royal Free Hospital), Neal Patel (The Whittington Hospital), Shern Wai Koh (North Middlesex University Hospital), James Hernon (Norfolk And Norwich University Hospital), Giuseppe Preziosi (Queen Elizabeth The Queen Mother Hospital Margate), Hermes Manos (Victoria Hospital Kirkcaldy)

United States: Eli Adams (OSF Saint Francis Medical Center).

Yemen Rep.: Mohammed Al-Shehari (Al-Thawra Modern General Hospital).

#### Collaborators

Algeria: Benali Tabeti, Aicha Bengueddach, Anouar Remini, Hakima Kehili, Nabil Boudjenan Serradj, Nacim Ikhlef, Noureddine Chadeli, Hakim Larbi, Abdelkader Menasria, Chakib Behilil, Mohammed Elamine Meghaizerou (Ehu-1st November 1954).

Australia: David Proud, Nastassia Shulman, Wael Jamel, Hwa Ian Ong, Anh Vu, Felix Wang (Austin Hospital); Stephen Smith, Luke Peters, Saksham Gupta, Shane Zhang, Isaac Caitens, Colin House, Thomas Capomolla, You Jin Han, Timothy Walker, Peter Martin (Calvary Mater Newcastle); Kelvin Kwok, Mitchell Gooch, Shirley Cai, Jonathan Tandjung, Vanessa Ng, Joyce Wang, Emily Reid, James Cui, Dennis Shen, Andrew Li, Shawn Ng, Angeline Sathiakumar, Amanda Dawson, Jess Barklimore, Benjamin Julien, Gemma Qian, Janae Chew, Riya Bhatia, Jenny Siu, Nicole Li, Chakraborty Oung, Peter Hamer, William Lai, Sarah Maguire, Adi Naarayanan, Chi Wen Un (Gosford Hospital); Francesco Amico, Myrna Ishak, Linda Beukes, Aiden Cheung, Leannedra Kang, Auston Yu, Mina Al Tawel (John Hunter Hospital); Elizabeth Murphy, Yick Ho Lam, Carolyn Chew, Antonio Barbaro, Hong Lee, Charlotte Stennard, Angelyn Khong, Dylan Morley (Lyell McEwin Hospital); Matthias Wichmann, Alicia Lim, Ishraq Murshed, Mary Zhao, Stephanie Louey (Mount Gambier And Districts Health Service); Russell Hodgson, Zainab Naseem, Allen Xiao, Junghyun Nam, Shady Rizk, Kieran Benn, Emma Haege, Laura Bland, Mark Slavec, Kexin Sun, Yining Huang (Northern Hospital); Tarik Sammour, Luke Traeger, Hakim Fong, Yu Zhou, Jiangyu Zhou, Nicole Kate, Divyanshu Joshi (Royal Adelaide Hospital); Ruben Rajan, Alex Mimery, Talia Shepherd, Megan Banks, Tamika Bland, Bhargav Jayani, Conor Nash (Royal Perth Hospital); Abdallah Elsabagh, Pauline Miller, Joel Stein, Harshit Morisetty, Deanna Lee, Faisal Mohammed (St John Of God Midland Public And Private Hospital); Darren Tonkin, Dominic Parker, Qiwen Wang (The Queen Elizabeth Hospital); Richard G McGee, Shubhang Hariharan, Elizabeth Lun, Yannick De Silva, Betty Wang, Hugh Elbourne, Darvesh Singh Maan (Wyong Public Hospital).

Bosnia And Herzegovina: Samir Delibegovic, Mirhan Salibasic, Amela Komilija Efendic, Merima Kruscica, Emsad Halilovic (Clinical Center University Of Sarajevo).

Bulgaria: Dzhevdet Chakarov, Elena Hadzhieva (Umhat Sveti Georgi); Dobromir Dimitrov, Paulina Vladova, Meri Shoshkova, Martin Karamanliev, Aparajeya Shanker, Ananya Mehta, Mohamed Abdullahi, Vysakh Ratheesh, Vasan Kamalathevan, Anandu Sundar, Saheed Owolabi Shittu, Susanna Koshy Thomas, Chandini Vijay Kurup, Munazza Khan, Shashwat Shanker, Elahe Naghavi, Mitchelle Bernard D Silva, Christina Wiesner (University Hospital Dr Georgi Stranski).

Colombia: David Baquero, Juan Sebastian Lopez Figueroa, Daniela De La Ossa-Posada, Manuela Arbelaez-Gomez, Camilo Calderón Cano, Fernanda Villa Quijano, Juan Manuel Bermudez, Isabella Osorno Munoz, Manuela Restrepo Molina, Lorenzo Neira, Natalia Montes Suaza (Pablo Tobon Uribe Hospital).

Croatia: Dario Vukosav, Ivan Zekanovic, Ivanica Zupan, Jakov Mihanovic, Ivan Bacic (Zadar General Hospital).

Egypt: Mohamed Al Sayed, Mohamed Zidan, Hashem Altabbaa, Yousef Tanas, Youssef Kerolous, Mohamed Mokhtar, Youssef Okazy, Yasmine Seada, Marwan Bahnacy, Mostafa Seif El Deen, Abdelrahman Zidan, Albaraa Daradkeh, Osama Al Shaqran, Stephanie Hanna, Ahmed El Banna, Yassin Badr, Heidi Sherif, Abdalrhman Abousetta, Yasmena Gaber, Mohamed Mahmoud, Youmna Abourady, Mohamed Basha, Tarek Zaho (Alexandria Main University Hospital); Mohammed F. Ramadan, Mohamed Ibrahim, Abdallah Hussein, Sherif Alaa, Ahmed M. Kedwany, Eman Mohamed, Sarah Khaled, Esraa Kotb, Alshymaa Ebrahim, Samah Arafa, Moaiad Eldin A. Mohamed, Afnan Morad, Fatma Monib, Randa Soliman, Moamen Shalkamy, Mariam Nageh, Shehab Eldin Saad, Sohayla Youssef, Mohamed Shazly, Sahar Hassan, Mohamed Ragab, Asmaa Sedeek, Omar Arafat Mahmoud, Omar Salah, Abdelrahman A. Abdelrahman, Shahy Wael, Mariam Rayan, Mohamed Tarek Mahmoud, Amal Refaie, Gehad Kamel (Assiut University Hospital); Mohie El-Din Mostafa Madany, Sarah Abdelmohsen (Aswan University Hospital); Mohammed Bedair, Dina Hamed, Ahmed Saber Mohamed Abdelrahman (Giza International Hospital); Ahmed Abdallah, Abdullah N Nassar, Medhat Katry (Oncology Center Mansoura University); Moatassem Erfan, Mennatallah Elnokity, Shimaa Atiya, Mahmoud Reda, Dunia Mowafy (The Memorial Soaad Kafafi University Hospital).

Germany: Daniel Reim, Marie Christin Weber, Maximilian Berlet, Maximilian Kiessler (Technical University of Munich, School of Medicine and Health, TUM University Hospital, Department of Surgery); Tim Vilz, Willis Maria, Jan Gortzen Patin, Martin Sohle, Florian Recker, Maria Wittmann, Achilles Delis, Markus Velten, Mumtaz Koksal, Max Oremek, Linda Gosejacob, Niko Knulle, Nils Sommer, Elena Aleksandrova, Mike Stanke, Sven Klaschik, Philippe Kruse, Christian Bode, Pascal Kowark, Ana Kowark, Mark Coburn, Daniel Beel, Jonas Dohmen, Maximilian Oremek, Leona Baier, Andrea Kunsorg, Claudia Neumann, Tobias Hilbert, Nadine Strassberger Nerschbach, Ricarda Neubauer, Nicolas Borter, Dorothea Protte (University Hospital Bonn); Jorg Kleeff, Johannes Klose, Onur Bayram (University Hospital Halle).

Greece: Georgios Kavalieratos, Aikaterini Sarafi (Agios Savvas Anticancer Hospital); Ioannis Tierris, Christos Chouliaras (Athens Medical Center); Dimitrios Korkolis, Konstantinos Fousekis, Nikolaos Tasis, Dimitrios Manatakis (Athens Naval And Veterans Hospital); Nikolaos V Michalopoulos, Maria Papadoliopoulou, Angelos I Nikolaou, Polyxeni Alexiou, Efthymios Poulios, Spyridon Christodoulou, Panagiotis Kokoropoulos, Ioannis Hatzaras, Nikolaos Danias, Panteleimon Vassiliu, Nikolaos Arkadopoulos, Theodoros Sidiropoulos, Maria Papadoliopoulou, Ioannis Margaris, Dimitrios Sampanis (Attikon University General Hospital); Stylianos Kapiris, Aikaterini Paraskeva, Michail Psarologos, Aikaterini Paraskeva (Evaggelismos General Hospital); Georgios Kapogiannatos, Aristotelis Nikitaras, John Katogiritis, Prokopis Christodoulou, Christos Ioannides, Ioanna Gogoulou (General Hospital Asklepieio Voulas); Konstantinos Bouchagier, Georgios Ioannis Verras, Levan Tchabashvili, Francesk Mulita (General University Hospital Of Patras); Savvas Symeonidis, Elissavet Anestiadou, Konstantinos Zapsalis, Nikolaos Ouzounidis, Stefanos Bitsianis, Lydia Loutzidou, Vasileios Foutsitzis, Antonia Aikaterini Bourtzinakou, Konstantinos Siozos (George Papanikolaou General Hospital Of Thessaloniki); Konstantinos Toutouzas, Nikolaos Intzes, Anna Mexi (Hippocratio General Hospital); Stylianos Kykalos, Panagiotis Dorovinis, Myrto D Keramida, Athanasios Syllaios, Adam Mylonakis, Markos Despotidis, Fotios Stavratis, Annita Loizou, Konstantinos S Giannakopoulos, Michail Vailas, Aikaterini Mastoraki, Islam Kourampi, Ilias Vagios, Aouatif Erasmia El Kanty, Emmanouil Mylonakis, Dimitrios Schizas (Laiko University Hospital); Elissaios Kontis, Eleni Papamattheou, Lykourgos Katsiaras, Andreas Efstathiou (Metaxa Cancer Hospital); Eleftherios Spartalis, Georgia Schismenou, Michael Spartalis (Sotiria General Hospital Of Thoracic Diseases); Konstantinos Lasithiotakis, Georgia Petra, Ioannis Tsikritzakis, Eftychia Nikolaou (University Hospital Of Heraklion Crete).

India: Yashwant Sakaray, Kanai Debnath, Satyam Khanna, Irrinki Santosh, Satyajit Sarangi, Varsha Khandelwal, Satish S N, Anmol Singh, Anand Kothari (Post Graduate Institute of Medical Education and Research).

Iraq: Moayad Al Nakeeb, Sahar Mezher, Mustafa Joudah, Ahmed Fakeri, Karrar Al Mosawi, Abdulla Abdulla, Hayder Jabbar, Ahmed Al Janabi (Al-hussien Medical City).

Ireland: Helen Heneghan, Tom Gallagher, Robert O’Connell, Kealan Blake, Conor Gleeson, James O’Grady, Kate Eustace, Lily Farrell, Solveig Svendsen (St Vincent's University Hospital).

Italy: Stefania Cimbanassi, Maria Danieli, Michele Altomare, Andrea Spota, Roberto Bini, Giuliano Santolamazza, Martina Aguzzi, Francesca Nava, Francesco Virdis, Federica Renzi, Osvaldo Chiara, Margherita Carbonaro, Pietro Calcagno, Pietro Lombardi, Pietro Achilli, Lorenzo Morini, Bruno Alampi, Giovanni Ferrari, Vincenzo Nicastro, Irene Giusti, Pietro Carnevali (Asst Grande Ospedale Metropolitano Niguarda); Francesco Pata, Mariasara Osso (Azienda Ospedaliera Di Cosenza); Fabrizio Vittadello, Chiara Girotto, Andrea Grego, Enzo Mammano, Emanuela Tessari, Antonio Rella, Alvise Frasson, Luca Faccio, Giacomo Sarzo, Caterina Barrella, Emanuela Tessari (Azienda Ospedaliera Di Padova); Corrado Pedrazzani, Sabrina Zambelli Sopalu, Gabriele Gecchele, Angelo Di Vittori, Noemi Bicelli, Ernesto De Giulio, Riccardo Giuri, Giacomo Faccioli (Azienda Ospedaliera Universitaria Integrata Di Verona); Danilo Centonze, Costanza Distefano, Roberta Granata, Giordana Riccioli, Ludovica Stella, Rossella Gioco, Salvatore Costa, Domenico Zerbo, Massimiliano Veroux, Alessio Licciardello (Azienda Ospedaliero- Universitaria Policlinico San Marco); Adolfo Pisanu, Valentina Murzi, Tiziana Pilia, Federica Campus, Carla Piras, Piergiorgio Serra, Alessandra Saba, Mauro Podda, Emanuela Gessa, Paola Marongiu, Alessandro Cois, Federico Corronca, Silvia Montisci, Eleonora Locci, Marcello Pisano, Eleonora Silanos, Alessandro Carta (Cagliari University Hospital); Paola Germani, Sara Pepe, Davide Drigo, Letizia Cecchini (Cattinara University Hospital); Mauro Santarelli, Enrico Potenza, Beatrice De Zolt Ponte, Elena Montanari, Victor Ugo De Donato, Lorenzo Capello, Sara Galati, Micol Giuliano, Diego Visconti, Luca Benedetto Lo Piccolo, Sofia Gamba, Chiara Celano (A.O.U. Città della Salute e della Scienza di Torino); Domenico Soriero, Giacomo Carganico, Davide Pertile (Irccs Ospedale Policlinico San Martino); Paolo Delrio, Teresa Pagano, Daniele Sannino, Carmela Cervone, Alessia Aversano (Istituto Nazionale Tumori Fondazione); Enrico Restini, Ivana Conversano, Rocco Tumolo, Marco Varesano, Grazisa Scialandrone (Lorenzo Bonomo); Salomone Di Saverio, Marziali Irene, Martina Zambon, Grazia Travaglini, Laura Lely, Alberto Buonanno (Madonna Del Soccorso Hospital); Isabella Mondi, Eleonora Ciccioli, Federico Cavallari, Andrea Lana, Fjorenta Sulo, Mario Biral, Rolando Tasinato, Sebastiano Pillirone, Daunia Verdi (Mirano Hospital); Giorgio Ercolani, Francesca Tauceri, Leonardo Solaini, Daniela Di Pietrantonio, Massimo Framarini, Chiara Casadei (Morgagni-Pierantoni Hospital AUSL Romagna); Elise Pouli, Luca Schiavone, Alessandro Broglio, Martina Martorana (Ospedale Civile Di Voghera); Francesco Roscio, Anna Laureys, Tea Bergenti (Ospedale Di Circolo Di Busto Arsizio); Gianandrea Baldazzi, Marta Spalluto, Massimiliano Ardu, Giovanni Tarchi, Camillo Franzetti (Ospedale Di Legnano); Lorenzo Maria Fatucchi, Osvaldo Carpineto Savorani, Leonelle Lore Nguefack Noudem (Ospedale San Donato Usl Toscana Sud Est); Lorenzo Ripamonti, Giulia De Carlo, Veronica Brocco, Elenasofia Signaroli, Andrea Scacchi (IRCCS San Gerardo dei Tintori - Monza); Emanuele Romairone, Sara Marzorati, Lorenzo Epis, Salvatore Carrabetta, Francesco Floris, Andrea Pierre Luzzi, Ugo Giuseppe Ribeca, Raquel Diaz, Alice Filippelli, Salvatore Carrabetta, Dorena Caruso, Fabrizio Ballari, Carolina Righetti, Pietro Grondona, Francesca Re (Ospedale Villa Scassi); Vincenzo Lizzi, Marco Montagna, Alessandra Giuliani, Francesco Maffei, Rocco Melino, Nicola Tartaglia, Fernanda Vovola, Giovanna Pavone, Mario Pacilli, Alberto Gerundo, Giovanni Di Gioia (Ospedali Riuniti Azienda Ospedaliera Universitaria Foggia); Andrea Mingoli, Gioia Brachini, Bruno Cirillo (Policlinico Umberto I); Eugenio Cucinotta, Santino A Biondo, Teresa Sinicropi, Francesco Fleres, Giuseppe Martorana, Vincenzo F Tripodi, Carmelo Mazzeo (University of Messina, Messina); Alessandro Spaziani, Emanuela Basile, Gaetano Poillucci (San Matteo Degli Infermi); Martina De Leonardis, Irene Tucceri Cimini, Danilo Meloni, Antonella Grasso (San Salvatore Hospital, University of L’Aquila); Maurizio Cervellera, Lodovico Sartarelli, Claudia Mongelli, Francesco Bagnardi (Santa Annunziata Hospital); Giovanni Terrosu, Lara Bonello, Davide Muschitiello, Vittoria Morinelli, Federica Passafiume (Ospedale Santa Maria della Misericordia di Udine); Antonello Deserra, Alessandro Cannavera, Francesca D Agostino, Carla Margiani, Cristina Murru, Giada Pattaro, Barbara Demurtas (Santissima Trinità - Ats Sardegna); Giuseppe Ietto, Simone Gianazza, Elisa Monti, Giacomo Borroni, Stefano Megna, Eugenio Cocozza, Lorenzo Livraghi, Roberto Delpini, Mattia Berselli, Davide Inversini, Enrico Ferri, Andrea Palillo, Domenico Iovino, Sabrina Garbarino, Alessandra Zullo, Chiara Peverelli, Valeria Quintodei, Valentina Marchionini, Lorenzo Conti, Alessandro Marzorati, Elisabetta Marta Colombo, Murad Odeh, Giuglio Carcano, David Meierruth (University Of Insubria)

Jordan: Mohammad Salah, Mus Ab Elatrash, Faris Abbadi (Al-Tafilah New Hospital); Motasem Almaletti, Yanof Al Naggar, Mohammad Salah, Sief Addeen Al Tahayneh (Islamic Hospital); Mohammad Sami Elmuhtaseb, Osama Abdul Kareem Sarhan, Almu’atasim Khamees, Raghad Yousef Yassin, Khalifa Salem Augi, Noorelhuda Kamal Abubaker, Seba Mahmoud Alghananeem, Ayat Khaled Mohsen, Aseel Ahmad Rezeq (Jordan University Hospital); Khaled Obeidat, Mahmoud Mahafdah, Saleh Shammakh, Hasn Haj Freej, Suleiman Mahafdah (King Abdullah University Hospital/ Jordan University Of Science And Technology); Anas Aljaiuossi, Mohammad Tanashat, Obieda Altobaishat, Samah Alananzeh (Princess Basma Hospital).

Latvia: Kristine Pavlovica, Daniella Zvina, Anzelika Beikule, Hoshika Tharni Sivapalan, Aija Tumova, Ilvija Knospina, Alise Antuanete Snikere, Anna Borisova, Sibi Anpalagan, Luxsana Mathiyalagan, Zhizi Yang, Andrejs Tolstiks, Nityanand Jain, Renate Ruta Apse, Asnate Matroze, Emija Nikola Karele, Sarmishtha Sharma, Deepkanwar Singh Panag, Aybaniz Ahmadova, Rohela Amiry, Olegs Zasibajevs (Pauls Stradins Clinical University Hospital).

Libya: Mostafa Elawami, Naseralla Elsaadi, Ebtisam Elbraky, Azza Eloshibi, Fayrouz Hweidy, Asma Moftah, Wijdan Sayfulnasr, Randa Ateh, Montaser Benzayed (Benghazi Medical Center); Hamida El Magrahi, Abir Ben Ashur, Salem Ali (Crown Health Care Clinical Team); Hussain Aboudlal, Akram Alkaseek, Haitam Shames, Anas Salim Abd Zayed, Amyirah Alshiteewi (Gharyan Central Hospital); Yamen Bobaker, Fatama Salem, Najway Tahir, Fatama Salem (Tobruk Medical Center); Sharfeddin Barka, Reem Ghmagh, Entisar Alshareea, Eman Abdulwahed (Tripoli Central Hospital); Ali Amer Ghummied, Suhaylah Timmalah, Serien Hossain Bensalem, Duaa Eisa Omar, Mohamed Alsori, Amira Ali Ashour, Rawia Jamal Hafed Ghmagh, Sara Mohammed Ammar Alsagheer (Tripoli Medical Center/ Tripoli University Hospital); Majdolin Miloud Almahjoub, Mustafa Ahmed Ekhail, Hajir Salem (Zliten Teaching Hospital)

Lithuania: Tomas Poskus, Marius Kryzauskas, Matas Jakubauskas, Kristina Marcinkeviciute, Ugne Silinskaite, Augustas Poskus (Vilnius University Hospital Santaros Klinikos).

Malaysia: Zaidi Zakaria, Michael Pak Kai Wong, Maya Mazuwin Yahya, Mohd Nizam Md Hashim, Wan Mokhzani Wan Mokhter, Wan Zainira Wan Zain, Siti Rahmah Hashim Isa Merican, Rosnelifaizur Ramely, Ikhwan Sani Mohamad, Syed Hassan Syed Abd Aziz, Jien Yen Soh, Mohd Azem Fathi Mohammad Azmi (School of Medical Sciences & Universiti Sains Malaysia Specialist Hospital); Tak Loon Khong, Sui Weng Wong, Mohamed Rezal Abdul Aziz, Amanda Weng Yee Leong, Wei Yang Heng (University Malaya Medical Centre).

Mexico: Mario Trejo Avila, Asya Zubillaga Mares, Daniel Astorga Guardado, Alejandra Nunez Venzor, Minnet Serrano Sanchez, Javier Andres Meza Hernandez (Hospital General Dr. Manuel Gea González); Noel Salgado Nesme, Oscar Santes Jasso, Danilo Tueme de la Peña, Oscar Posadas Trujillo, Daniel Doniz Gomez Llanos, Emilio Sanchez Garcia Ramos, Alberto Najera Saldana, Florencia Lucero Serrano, Jorge Canto Losa, Luis Davila Sanchez (Instituto Nacional De Ciencias Médicas Y Nutrición ‘Salvador Zubirán’).

Morocco: Elmehdi Boutajanouit, Youness Belattar, Hind Essalim, Ayman Echab, Mohamed Sami Melouane, Soulaimane Laaziri, Imane Oujaa, Ayoub Bousselham (Hopital Ibn Tofail); Amine Souadka, Mohammed Anass Majbar, Amine Benkabbou, Saloua Kassad, Arwa Aboumedian, Fatima Zahra Aboumedian, Othman Arsalan (Institut National D'oncologie).

New Zealand: Wal Baraza, Cameron Wells, Viha Vig, Genevieve Groult, Ashley Pereira, Josheel Ram, Hannah Stone, (Auckland City Hospital); Tamara Glyn, Mathew Morreau, Maia Wehi, Andreas Nicolaou, John Lu, Niamh Kilpatrick, Stella Daniell, Laura Sunderland, Zoe Williams, Analise Wang, Samuel Tomkins (Christchurch Hospital); Sze Lin Peng, Cameron Wells, Hannah Ashmore Price, Omar Nosseir, Marta Simonetti, Megan Singhal (Middlemore Hospital); Siraj Rajaratnam, Eileen Song, James Jin, Xiao Shen Hu, Caroline Stokowski, Anamitra Nair, Li Mei Gan, Vishvini Vijayakumaran, Si Jia Ong, Lisa Smith, Calvin Fraser, Varun Modi, Ethan Figgitt, Naeun Hwang (North Shore Hospital); Laura Brockie (Taranaki Base Hospital); Jeremy Rossaak, Binura Lekamalage, Jonathan Johns, Sophia Xu, Rebecca Veitch, Sunny Ding, Consuelo Alarcon, Yash Shahri, Claudia Thomas, Julia Shearer, Daniel Carson (Tauranga Hospital); Jesse Fischer, James Uivaa, Monique Mahadik, Simon Lai, Emily Joe, Shaamnil Prasad, Maitreyi Jain, Ajay Bansal, Olga Korduke (Waikato Hospital); Sarah Rennie, Anahera Herewini, Clare Mcinerny Heather, Greer Jackways, Christina Gordon (Wairarapa Hospital); Anthony Lin, Sue Ong, Jeffery Tan, Mohammed H Alsinan, Tharinya Gamalath, Annelies Bardoul, Mairarangi Haimona (Wellington Regional Hospital); Christopher Harmston, Matthew Mcguinness, Oscar De Langen, Andrew Mcintyre Robinson (Whangarei Hospital).

Nigeria: Garzali Ibrahim Umar, Mustapha Abdullahi Muhammad, Tijjani Nasir Nagwamutse, Khadija Abdullahi Ado, Rifkatu Nasiru Alhassan (Aminu Kano Teaching Hospital); Oludolapo Afuwape, Oyeyemi Dada, Vincent Osoka, Adigun Ishola, Nurudeen Akinbami (University College Hospital); Samuel Olatoke, Taofiq Raji, Olushola Fasiku, Olufemi Arinde, Olayide Agodirin, Solomon Irmiya, Olalekan Agede, Saburi Oyewale (University Of Ilorin Teaching Hospital).

Pakistan: Tabish Chawla, Annam Kafeel, Madeeha Ali, Izza Tahir, Muhammad Mushahid Hussain Rizvi, Alinah Qureshi, Aqsa Amjad, Mohammad Shahzaib Qadir, Muhammad Mobeen Shahid, Muhammad Tabish Nasim, Saniya Waseem (Aga Khan University).

Peru: Cesar Huaroto Landeo, Juan Carlos Luna Cydejko, Diego Chavez Hernandez (Clinica Internacional).

Poland: Jakub Migoń, Michał Bąk, Adrianna Pielech, Natalia Torz, Jan Nicikowski, Joanna Osmólska, Dawid Murawa (University Hospital of Karol Marcinkowski in Zielona Góra).

Portugal: Helder Alem, Catarina Santos, Nuno Mendonca, Patricia Bernardo, Sara Patrocinio, Frederico Nazareth, Lourenco Moniz (Centro Hospitalar Barreiro Montijo); Catia Ferreira, Goncalo Guidi, Carolina Marques, Clara Leal, Andre Silva, Margarida Dupont (Centro Hospitalar De Trás-Os-Montes E Alto Douro); Pedro Silva-Vaz, Manuel Rosete, Maria João Amaral, Catarina Lopes, Rita Andrade, Diogo Paula, Tiago Antunes, Ana Filipa Vilela, Cristina Camacho, Ana Ruivo, Ines Prior, Adriana Ferreira, Pedro Pinto, Mariana Lemos, Ana Claudia Raposo, Raquel Teixeira (Centro Hospitalar E Universitário De Coimbra - Hospital Geral); Luisa Frutuoso, Joana Antunes, Egon Rodrigues, Penelope Correia, Miguel Magalhaes, Luisa Magno, Pryangka Martins, Maria Iglesias (Centro Hospitalar Entre O Douro E Vouga); Pedro Febra, Elisabete Pacheco, Joana Domingues, Rita Galama, Ines Seixo, Raquel Lalanda (Centro Hospitalar Médio Tejo); Miguel Cunha, Edgar Amorim, Juan Rachadell, Sofia Pina, Ines Miguel, Beatriz Dias, Pedro Almeida, Barbara Santos, Catarina Santos (Centro Hospitalar Universitario Do Algarve - Unidade De Portimão); Rita Camarneiro, Leticia Heeren, Regina Silva, Andrea Abreu, Barbara Tinoco (Hospital Das Caldas Da Rainha - Centro Hospitalar Do Oeste); Olena Teslyak, Aldo Jarimba, Renato Barradas, Beatriz Louro, Rita Lourenco, Nadia Marcos (Hospital De Santarem); Manuel Damasio Cotovio, Arnaldo Machado, Martim Rente, Rita Lima, Maria Isabel Pereira, Ines Matias, Ma Madalena Siqueira, Joana Bolota, Sofia Leandro (Hospital Do Espirito Santo); Diogo Sousa, Jessica Ricardo, Marilia Ferreira, Diana Stoian (Hospital Do Litoral Alentejano); Sandra Carlos, Brigitta Cismasiu, Ana Lucia Barreira, Joao Vaz, Francisco Vara Luiz, Miguel Palas, Susana Henriques, Jose Miguel Carlos (Hospital Garcia De Orta); Catarina Quintela Silva, Catarina Mesquita Guimaraes, Lilian Farias, Ligia Freire (Unidade Local De Saude De Matosinhos - Hospital Pedro Hispano).

Qatar: Zia Aftab, Ali Toffaha, Mohamed Abunada, Ammar Aleter, Mohammad Al Yaseen, Sherif Ahmed, Amjad Parvaiz, Mohamed Kurer, Omer Al Yahri, Murad Alahmad, Mohamed Khalaf, Mahmood Al Dhaheri, Ayman Ahmed, Salwa Sidahmed, Mahwish Khawar, Maram Salah (Hamad General Hospital).

Russian Federation: Tatiana Khorobrykh, Albina Zubayraeva, Akmalbek Otabekov, Aleksandra Koziy, Polina Panova, Bogdan Semchenko, Anna Rebrova (Im Sechenov First Moscow State Medical University); Aleksandr Butyrskii, Ferat Suleymanov, Server Kareymanov (Municipal Emegency Hospital No.6); Andrei Bazaev, Armen Kokobelan, Iliya Zhukov, Andrei Malov (Privolzhsky Research Medical University).

Slovenia: Jan Grosek, Tajda Kosir Bozic, Jurij Ales Kosir, Ales Tomazic (University Medical Centre Ljubljana).

South Africa: Adam Boutall, Vuyolwethu Soldati, Alex Muturi, Simphiwe Gumede, Zubeir Salie, Shrikant Peters, Kathryn Nieuwenhuis, Mayilan Chetty, Deborah Obeng, Robyn Brown, Bruce Biccard, Margot Flint (Groote Schuur Hospital).

Spain: Izaskun Balciscueta, Carla Leal, Christian Esteo, Christian Esteo, Sara Garcia, Jorge Febre, Beatriz Cueno (Hospital Arnau De Vilanova); Jose Martin Arevalo, Ana Izquierdo Moreno, Sara Palomares Casasus, Luisa Garzon Hernandez, Pablo Moya Marcos, Alba Perez Del Pozo, David Moro Valdezate (Hospital Clínico Universitario De Valencia); Carlos Guijarro Moreno, Ana Sanchez Gollarte, Enrique Gonzalez Gonzalez, Armando Galvan Perez (Hospital Del Henares); Luis Miguel Jimenez Gomez, Silvia Perez Ajates, Maria Sanchez Rodriguez, Monica Ballon Bordo, Alvaro Landeras Lopez, Carlos Morales Garcia, Daniel Velayos Herraez, Jose David Gonzalez Esteban, Cristina Rey Valcarcel, Monica Ballon Bordo, Jose David Gonzalez Esteban (Hospital General Universitario Gregorio Marañón); Sebastian Fernandez Arias, Leire Garcia Alonso, Pablo Del Val Ruiz, Ainoa Fraile Gonzalez (Hospital Universitario Central De Asturias (Huca)); Cristina Vera Mansilla, Manuel Diez Alonso, Belen Matias Garcia, Fernando Mendoza Moreno, Alma Blazquez Martin, Ana Quiroga Valcarcel, Enrique Ovejero Merino, Lucia Diego Garcia, Lucas Casalduero Garcia, Alberto Vilar Tabanera, Yousef Allaoua Moussaoui (Hospital Universitario Principe De Asturias); Jesus Abrisqueta, Angela Alcarazo, Noelia Ibanez (Hospital Universitario Virgen De La Arrixaca); Virginia Duran Munoz Cruzado, Carlos Javier Garcia Sanchez, Beatriz De Los Angeles Ruiz Garcia, Marta Garcia Corona, Paula Bravo Raton (Hospital Universitario Virgen Del Rocio); Hanna Cholewa, Vicent Primo, Blas Flor, Pablo Guerrero, Marta Nieto Sanchez, David Plazas, Alba Serrano, Jorge Sancho Muriel, Monica Millan, David Quevedo (Hospital Universitario Y Politécnico La Fe); Begona Estraviz Mateos, Manolo Leon Valarezo, Ana Uriguen Echeberria, Jaime Gonzalez Taranco, Laura Fernandez Gomezcruzado, Izaskun Markinez Gordobil (Hospital Urduliz); Maria De Los Angeles Mayo Ossorio, Ainhoa Maestu Fonseca, Alicia Inmaculada Alvarez Gonzalez, Ana Maria Camacho Oliva (Puerta del Mar University Hospital); Gerardo Chamoso Mialdea, Ricardo Jesus Castro Lara, Antonio Garcia Dominguez, Mercedes Estaire Gomez (Severo Ochoa University Hospital).

Sri Lanka: Malith Nandasena, Niruban Ganesarajah, Harikrishanth Shanmugaraj, Kalaiyukan Sathasivam, Dulanjana Ranasinghe, Minidu Chandraguptha, Hasangi Gamage, Shalika Nagasinghe, Jeewantha Senavirathna, Yohan Chamara, Kanchana Wijesinghe (Colombo South Teaching Hospital); Dakshitha Wickramasinghe, Deshan Gomez, Salma Salih, Duminda Subasinghe, Thamisha Nugaliyadda (National Hospital Of Sri Lanka).

Sudan: Mutasim Mursi Abubaker, Mojahid Hamdan Ahmed, Mohamed Musa Yassin, Ola Abdallah Eljizoly, Ahmed Mohamed Ibrahim Mohamed, Mohamed Ahmed Adam, Mohammed Salah Abduelrahman, Wtfaa Siddeg, Said Ibrahim (Gadarif Teaching Hospital); Elsamoul Abdulgafoor, Hayat Abuobaida, Essam Eldien Abuobaida (Ribat University Hospital).

Switzerland: Frederic Ris, Vaihere Delaune, Jeremy Meyer, Enrica Chiriatti, Isabelle Uhe, Abdul Ghyasi, Andrea Peloso, Emilie Liot, Guillaume Meurette (Geneva University Hospitals); Christos Andreou, Eliane Koller Brolese, Georgios Vergos, Joanna Naemi Marx, Lukas Eisner, Athanasios Tampakis (Kantonsspital Olten); Antonietta Petrusic, Popeskou Sotirios Georgios (Lugano Regional Hospital); Muhlhausser Julia, Stephanie Strauven, Anna Katharina Huber, Jorn Markus Gass (Luzerner Kantonsspital).

Syrian Arab Republic: Abdul Rahman Hammadieh, Gheed Abdul Khalek, Zienab Klib, Mohammad Rabee Mslmani, Kinan Nassar, Nafiza Martini, Majd Hanna (Al-Mouwasat University Hospital); Anwar Chammout, Abd Alwahab Alkhalaf, Shadi Haj Hussein, Hala Bakro, Ahmad Sajjee (Aleppo University Hospital).

Turkey: Afag Aghayeva, Gokalp Kagan Kurtoglu, Yilmaz Onat Koyluoglu, Metincan Erkaya, Esra Dogan, Mehmet Metin Yurttaser, Bengi Agca, Goktug Mert Kurtoglu, Abdurrahman Furkan Cetisli, Elvin Ay, Fran Ivan Cubranic, Sara Matulic, Emre Tunccan (Acibadem Altunizade Hospital); Tayfun Karahasanoglu, Melik Kagan Aktas, Bengu Togay, Ece Ada, Ilayda Esma Yavuz, Emre Tuzuner, Ismail Ahmet Bilgin, Tayfun Karahasanoglu, Mert Tanal, Bilgunay Ilkin Safa, Atahan Durbas, Yagmur Karatas, Bilgesu Duman, Furkan Demiral (Acibadem Maslak Hospital); Tayfun Bisgin, Muhammet Berkay Sakaoglu, Turugsan Safak, Mevlut Bugra Baysal, Beste Yildirim, Onur Karaagac (Dokuz Eylul Univ. Hospital); Osman Bozbiyik, Busra Kucukates, Sarp Tunali, Suleyman Unal, Ayse Tuzcuoglu, Eylul Turksever, Martin Andonov Mitkov, Kaan Okumus, Mert Anil Altun, Zeynep Selin Savas, Mustafa Ali Korkut, Ferda Ozkan, Tayfun Yoldas, Bahadir Emre Baki, Firat Basci, Zekeriya Erhan Akgun, Ilay Suleyman, Sonmez Sehim, Ece Irem Zaman, Batuhan Cakmak, Mehmet Baskayali, Berk Goktepe, Recep Temel, Gunay Huseynova, Ozge Kilinc, Sinem Kilicat, Sevgi Sena Demirci, Irem Guneri, Nilgun Izmirli, Rabia Sultan Atahan, Karya Islamoglu, Baris Ozkilic (Ege University Hospital); Ahmet Aslan, Sadik Kesmer, Banu Yigit, Serkan Yilmaz (Elazig Fethi Sekin City Hospital); Mustafa Oncel, Alparslan Saylar, Abdullah Emre Askin, Sebnem Bektas, Aylin Izgis, Mustafa Sefa Isin, Gozde Deniz, Meltem Yasar, Eyup Deniz (Istanbul Medipol University Hospital); Server Sezgin Uludag, Mehmet Faik Ozcelik, Haktan Ovul Bozkir, Berke Buyukarikan, Mehmet Erinc Onal, Didem Gulhan (Istanbul Universty - Cerrahpaşa Medical Faculty); Arif Atay, Hakan Kul, Mert Yoldas, Ozgem Uysal, Ali Alcan, Ayse Beyza Acik, Bilal Batuhan Yenen, Ezgi Su Albayrak, Furkan Baysal, Gulce Ozkayahan, Mehmet Oguz Pinar, Kaan Turmus, Mete Numan Etlik (Izmir Katip Celebi University Faculty of Medicine); Mustafa Cem Terzi, Semra Demirli Atici, Aras Emre Canda (Acibadem Izmir Kent Hospital); Mehmet Ulusahin, Adnan Gundogdu, Muhammet Ates, Hatice Bulut, Sule Sevim, Zeynep Kamak Surmen, Tayfun Surmen, Beyza Nur Ekmekci, Ayse Nilufer Yuzgec, Mustafa Deniz Tepe, Bilal Alkas, Merve Aktas, Ogun Bebek, Mahsima Gul Gumrukcu, Ece Karabulut, Mert Uzun, Mohammad Alnas Ah, Zeinab Danaei (Karadeniz Technical University Farabi Hospital); Emre Balik, Muhammed Ikbal Ates, Salih Nafiz Karahan, Mekselina Kalender, Furkan Camci, Arif Emir Narin, Dilara Yigit (Koç University Medical School); Tevfik Kivilcim Uprak, Ahmet Omak, Halil Ibrahim Sevindi, Sila Catal, Ozde Buda, Esin Zeynep Cinal, Burak Mert Saracoglu, Abbas Can Uludag, Asiye Sena Anli, Amer Salameh, Eminenur Sen, Muhammed Enes Tasci (Marmara University School of Medicine); Omer Faruk Ozkan, Elif Didem Terzi, Zafer Senol, Haron Cemel, Firat Demircan, Berkay Ozcan (Sultan 2. Abdülhamid Han Research and Training Hospital); Ibrahim Ethem Cakcak, Muhammed Samil Yekeler, Buse Ozkan, Selcen Demircioglu, Asli Kaya, Sarper Kizilkaya, Utku Yartasi, Eylul Senodeyici, Ilayda Bozkaya, Neslican Demir, Oya Budak, Arya Celikhasi, Mehmet Halil Keskin, Mutlu Can Pacaci, Kubra Nur Kabay, Mirac Ajredini, Merve Yaren Kayabas (Trakya University Hospital); Hamdi Ozsahin, Cenk Ersavas, Bulent Citgez (Uskudar University Faculty Of Medicine).

United Kingdom: George Ramsay, George Neelankavil Davis, Esyn Yeoh Ee Xin, Erica Ho Ching Tsoi, Prem Nagaraj, Stephanie Walker, Kirsty Luo-Yng Tay, Alishah Haider, Dominique Da Luz, Shubham Jain, Jasmine Luangboriboon, Anagha Chinmayee, Danielle Robertson, Tessa Yau, Miriam Lecerof, Savithri Sathivelu, Natthaya Eiamampai (Aberdeen Royal Infirmary); Michael Powar, Konstantinos Stasinos, Tanzil Rujeedawa, Hetta Friend, Jasmine Thomas, Wai Yan Ding, Lewis Witton, Fathima Manaal, Annie Zhao (Addenbrooke's Hospital); Colman Byrnes, Christopher Brown, Conor Mccollam (Antrim Area Hospital- Northern Health And Social Care Trust); Katharine Bevan, Abdul Hakeem, Seiver Karim, Jonathan Bennett, Zhenhao Lu, Arjun Sharma, Emily Smith, Iona Phillips, Ilias Epanomeritakis, Mathew Moolamannil, Anton Swarnan (Bedford Hospital); William Wallace, Aideen Campbell, Finn Mccann, Joyce Beshara, Catinca Ciuculete, Carlo Ferrazzano, Nial Connell, Sarah Mccandless (Belfast City Hospital); David Messenger, Shabnam Cyclewala, Matthew Kobetic, Alice Winch, Barbara Piecha, Tushar Rakhecha, Emma Baker, Stephen Downey (Bristol Royal Infirmary); Jamie Powell, Saidah Mohd Sahid, Chirag Goyal, Byapti Alice Nandi, Ethan Porritt, Chiara Dell Oro, Nikita James, Wafia Hussain, Ryhan Patel, Annie Alocious (Charing Cross Hospital); Natalia Sanchez Thompson, Sruthi Vatsavayi, Niharika Chokkapu (Chelsea And Westminster Hospital); Nicola Eardley, Aran Rees, Kaye Sparrow, Priyadarshini Padmanaban (Countess Of Chester Hospital); Manos Epanomeritakis, Jane Kilkenny, Donovan Campbell, Ronan Fegan, James Cartilage (Craigavon Area Hospital); Khalid Osman, Mazuin Abu Talib, Keith Mathew, Giustina Chu, Sulaymaan Al Majid (Darlington Memorial Hospital); Rina George, Jessica Banks, Freya Braddon (Doncaster Royal Infirmary); Hannah Easterbrook, Thomas Knowles, Cailin Ho, Zoe Austin (Dorset County Hospital); Panna Patel, Mohammed Kawsar, Ruiari Doherty, Zachary Campbell (Furness General Hospital); Thomas Hibbs, Paris Bruno, Mark Vipond, Fatima Sheerin, Euan Lewis, Eve Miller, Olivia Lark, Asha Jina, George Snell, Sajad Hussain, Anna Armstrong, Thomas Nicholls (Gloucestershire Royal Hospital); Deepa Bapu, Jessica Chang, Liam Phelan (Good Hope Hospital); Felicity Greenfield, Chris Thorn, Michael Okocha, Yegor Tryliskyy, Oceana Fernando, Alexander Kimish, Aminata Kaloko, Mya Patel Vathvali, Robert Seah, Farai Chiwah, Nirmitha Thayaparan, Sudais Naeem (Great Western Hospital); Alexis Schizas, Aliki Rompou, Kareem Omran, Leona Takeuchi, Hana Moattar, Ryan Sia, Duaa Faruqi, Jia Xin Khoo, Mosope Ajegbomogun, Nafisa Zilani, Miriam Frankl, Mahta Haghighat Ghahfarokhi, Hassan Kamal, Nadia Chowdhury (Guy's And St Thomas' Hospitals); Jalal Mohammad, Joseph Wyer (Heartlands Hospital); Vimal Hariharan, Dylan Whitaker, Shahin Zakeri, Jacinta Ngeh, Ehren Agarwal, Jianing You, Shiqing Ma, Katy Hempson (Hinchingbrooke Hospital); Sylvia Brown, Mishal Shahid, Nina Dworschak (Inverclyde Royal Hospital); Mark Bignell, Isobel Burridge, Shu Luk, Hanaa Asharaf, George Corby, Hanxiao Li, Hannah Nentwich, Imogen Wilkinson, Blessing Omorodion, Niamh Owens, William Thornton (John Radcliffe Hospital); Aarti Varma, Zoe Chia, James Bailey, Dylan Abeelack, Tanvi Mungale, Amrita Jandu, Arooj Qaiser, Rohit Ramesh, Rivya Mathews, Khafia Mehboob, Ashviny Ravindran, Ruta Dubinskaite (Lincoln County Hospital); Barrie Keeler, Ahmed Gendia, Dibyeshwari Rana, Aida Azlan, Lavanya Gupta, Raniah Al Saidi, Natasha Sheelam, Carlita Smith, Sonakshi Nemchand, Shreya Srikumar, Ragul Rajivan (Milton Keynes University Hospital); Dean Harris, Peter Cripps, Shwe Ooi, Samantha King, Lawrence Quach, Robyn Taylor, Colin Seel, Emma Harvey, Belinda Wang, Julia Bieniek, Bappy Basak (Morriston Hospital Swansea); Hema Sekhar, Mohammed Aradaib, Fouad Ashoush, Eleanor Kissane, Lucy Rimmer, Carol Koubaesh, Khan Mahrukh (Newcastle Upon Tyne Hospitals NHS Foundation Trust); Samer Zino, Ramy Shaalan, Vaishnevy Ganesh, Jenna Cook, Radhe Shantha Kumar, Sundas Butt, Rania Fernandes, Adibah Mohammad Amin, Joaquim De Sousa, Orla Busby, Tasnim Kouli, Sara Mohamed, Sameh Abdelwahab, Marcel Al Horoub, Shaimi Niraula, Sarah Virani, Ize Osagie (Ninewells Hospital); James Hernon, James Aldwinckle, Eunice Choi Yun Kwan, Dagmara Aulich, Francis Donya, Natalie Wheelhouse, Jungho Min, Sara Kazemzadeh, Mohammad Ali, Precious Ojo, Sho Giersztein (Norfolk And Norwich University Hospital); Lee Dvorkin, Ee Teng Goh, Michael Bath, Subramaniam Guru Naidu, Halil Hussein, Mamun Dornseifer, Niroshan Sivasothy, Lalana Songra, James Kersey, Tharindu Menushka Hansamal Galboda Liyanage, Shern Howe Koh (North Middlesex University Hospital); Elisabeth Drye, Mohamed Albendary, Abdulqudus Deeknah, Natasha Kowshik, Natasha Reid, Alwaleed Al Doory, Mikael Steede, Alan Hamda, Khai Saw (Peterborough City Hospital); Giuseppe Preziosi, Kulsum Maula, Nicole Claire Gentles, Aneeshka Nagpaul (Queen Elizabeth The Queen Mother Hospital Margate); Miss Catherine Boereboom, Hannah Boyd Carson, Francesca Ligori Malcom, Jahnavi Kalvala, Aashlesha Galla, India Jacklin Chatha, Manmeet Saundh, Titobiloluwa Coker, Ashrit Chohan, Bobbie Webster, Amelia Simenacz, Hawawu Muazu (Queens Medical Centre); Richard Slater, Alaa Obeida, Malaz Abbakar, Tariro Madziro, Hassan Farooq (Rotherham District General Hospital); Susan Moug, Hwei Jene Ng, Scott Macdonald, Aoife Carr, Emma Clark, Heather Craig, Rabia Ali, Mairi Cunningham, Mackenzie Green (Royal Alexandra Hospital); Nick Heywood, Alice Proctor, Sayed Mdabu, Khansa Irfan, Maneesha Weerasooriya, Amin Sohani, Joud Qasem, Shreya Sachdeva (Royal Blackburn Hospital); Nicholas Battersby, James Hughes, James Miller, Amy Stokes, Thibagaran Sathambihai, Dexter Sim, Alice Whitbread Abrutat (Royal Cornwall Hospital); Robert Bethune, Ben Rossi, Blazej Rybinski, Awais Chaudhary, Lavinia Cochetti, Sophie Thompson, Hannah Humphrey, Yara Lima De Mendonca, Pavel Loginovic, Eva Ruiz Daum, Burraq Imran (Royal Devon And Exeter Hospital); Jack Broadhurst, Kirsty Cole, Vinay Patel, Carlo Lori, Georgina Hart, Michelle John (Royal Hampshire County Hospital); Ondrej Ryska, Nader Helmy, Hamzah Amin, Elina Stokolova, Ferdos Rizgar, Saan Dyare, Fauzaan Syed, Shaheer Safdar (Royal Lancaster Infirmary); Mohamed Thaha, Theo Pelly, Yi Lun Khaw, Andreas Kakkou, Aleksandra Laguna, Jit Yih Tan, David Sinclair Thomas Junior, Anmol Kaur Dhaliwal, Navnit Kaur, Paraskevi Papatzanaki (Royal London Hospital); Alka Jadav, Edward Parkin, Ammarah Ughratdar, Bryant Chong, Namareq Ahmad, Maya Holt, Madeleine Truscott (Royal Preston Hospital); Muhammad Sajid, Abdul Malik Magsi, Pierre Jean Marie, Harveer Singh, Joey Fanstone, Keerthy Vijendran, Onyedi Moses, Courtney Ann Dennis, Reubeen Ahmad, Ioana Raducanu, Sana Jaan (Royal Sussex County Hospital); Isabella Sawyer, John Bunni, Bethany Wardle, Laura Gilliland, Florian Fischer, Isabelle Craner, Elise Bisson, Zara Coombes (Royal United Hospital Bath); Olivia Hurrell, Kathryn Mcwhirter, Shazna Bi, Ciara Mccaffrey, Christian Ward Bradley, Liza Buchynska (Royal Victoria Hospital); Jonathan Epstein, Emeka Nwokeocha, Oluwatobi Adegboye, Jeel Shukla, Imogen Phillips, Jude Geldart, Luqman Aizan, Hamza Shahbaz (Salford Royal Hospital); Rajeev Peravali, Alexia Farrugia (Sandwell General Hospital); Geeta Kaur, Sanjay Basu, Shaker Alseifi, Qaisar Razzaq, Ali Hamad, Michael Naguib, John Mcalister, Nicholas Dawson (Scunthorpe General Hospital); Paul Marriott, Zafar Shahbaz, Arun Osullivan, Harshita Buragapu, Abiramy Selvanathan, Lok Pong Cheng, Suki Bernstein (South Warwickshire NHS Foundation Trust); Cleo Kenington, Dimitra Peristeri, Kofi Cox, Zaynab Irfan, Sylvia Muthukkumaru, Isabelle Legood, Rishi Kumar, Raveena Khalsa, Joshua Asto, Charlotte Evans (St George's Hospital); Nasira Amtul, Candice Downey, Zaynab Hafeji, Elizabeth Burdekin, Sannah Jamil, Emily Armstrong, Bethany Bracewell, Alexander Randall, Liam Murphy, Daniel Kyeremateng, Lavesh Mirpuri, Shan Sunny, Lorna Blackmore, Sophie Price, Sagar Sanadi, Claire Hardy, Michaela Silver, Ruby O Loughlin, Benedict Mallucci, Rebecca Hakim (St James's University Hospital Leeds); James Kinross, Laura Tincknell, Yasmin Owadally, Pratik Ramkumar, Shneeza Gill, Dean Chughtai, Tianyu Song, Akshita Ramineni, Srikar Reddy Namireddy (St Mary's Hospital); Arnold Goede, Rachel Carten, Tomas Urbonas, Mariah Mwipatayi, Ameer Khamise, Santhosh Ayirookuzhi, Iona Banerjee, Beatrice Sorce, Anna Deal, Keya Agadi, Grace Scopes, Ali Waleed Khalid (Stoke Mandeville); Hasan Mukhtar, Omar Ugas, Harpreet Sekhon, Jessica Nyon, Aryan Goel, Drew Leamon, Harkirat Dhaliwal, Fope Adedeji, Zenel Qenami, Khadija Zribi, Juliet Kenstavica Pinto (The Whittington Hospital); Manish Chand, Tom Pampiglione, Taner Shakir, Hamza Mahmood, Adam Hussain, Lavine Liu, Sabrina Ali, Majlind Grozda, Rohma Shahzad, Vignesh Radhakrishnan, Hugo Ferreira, Aamina Mahmood, Mohammed Binyameen (University College London Hospital); Ghaleb Goussous, Vasileios Kalatzis, Sadhasivam Ramasamy, Emily Hall, Zaib Shamsi, Shiny Darwin, Tinashe Wadi, Julia Maciazek, Uzayr Sheikh, Ioannis Perros, Grace Bambury, Maanav Khanna (University Hospitals Of North Midlands); Michael Proctor, Alessandro Sgro, Julian Camilleri Brennan, Shannon Mcdonald, Jade Sheil, Rosie Young, Susan Chong, Susanne Marshall, Hermes Manos (Victoria Hospital Kirkcaldy); Jason Smith, Oliver Siaw, Zekiye Karagozlu, Akhil Sonecha, Ivin Jose, Haoze Yang, Ananya Jain, Andreas Zachariadis, Maryam Sheik, Hannah Hirji (West Middlesex University Hospital); Thomas Athisayaraj, Mojolaoluwa Olugbemi, Cheuk Man Lam, David Barnes, Lily Mainwaring, Aly Shaaban, Joanna Kucharczak (West Suffolk Hospital); Nicholas Ventham, Darren Porter, Emma Barron, Chantelle Sunley, Daniel Arbide, Reka Kovacs, Hariss G Paremes Sivam, Tze Yi Gan, Michelle Teo, Mathew Smith, Orieanna Reeve Chen (Western General Hospital); Conor Magee, Rebecca Reid, Farida Hegazy (Wirral University Teaching Hospital).

United States: Richard Anderson, Ayoolamide Gazal, Frank Disilvio, Eli Adams, Samantha Wahlers, Diego Ruiz-Avila, James Jackson, Devon Negron, Judy Suh, Kara Proctor (OSF Saint Francis Medical Center).

Yemen Rep.: Yasser Obadiel, Nashwan Tashan, Areej Alnajjar, Ragaa Albatool, Khloud Ali Saeed (Al-Thawra Modern General Hospital).

#### Data validators

Algeria: Anisse Tidjane (Ehu-1st November 1954).

Australia: Felix Wang (Austin Hospital); Hannah Legge Wilkinson (Calvary Mater Newcastle); Upuli Pahalawatta (Gosford Hospital); Ishraq Murshed (Mount Gambier And Districts Health Service); Yui Kaneko (Northern Hospital); Jessica Hanna (Royal Adelaide Hospital); Leesha Bryan (Royal Perth Hospital); Mohammad Faraz, Bethany Cooper (St John Of God Midland Public And Private Hospital); Denise Chia (Wyong Public Hospital).

Bulgaria: Mick Galasyuk (University Hospital Dr Georgi Stranski).

Colombia: Erika Gabriela Miranda (Pablo Tobon Uribe Hospital).

Croatia: Ivo Coza (Zadar General Hospital).

Egypt: Mohamed Al Sayed, Mohamed Zidan (Alexandria Main University Hospital); Sarah Abdelmohsen, Mohie Madany (Aswan University Hospital); Sarah Mansour (Giza International Hospital); Ahmed Wael (Oncology Center Mansoura University); Ammar Yasser, Mahmoud Reda, Dunia Mowafy (The Memorial Soaad Kafafi University Hospital).

Germany: Daniel Reim, Marie Christin Weber, Maximilian Kie Ler (Klinikum Rechts Der Isar Tum School Of Medicine); Rica Philippi, Kilian Schmidt, Vera Guttenthaler (University Hospital Bonn); Maria Schuler (University Hospital Halle).

Greece: Aris Plastiras, Theodoros Tsirlis, Dimitrios Korkolis (Agios Savvas Anticancer Hospital); Evangelos Fradelos (Athens Naval And Veterans Hospital); Pantelis Kalogerakos (Attikon University General Hospital); Elena Mavrodimitraki, Dimitrios Stergiou, Konstantinos Polyzois (Evaggelismos General Hospital); Sandra Maria Tsoti (General Hospital Asklepieio Voulas); Ioannis Maroulis, Ioannis Panagiotopoulos, Panagiotis Perdikaris (General University Hospital Of Patras); Odysseas Lomvardeas, Ourania Kerasidou (George Papanikolaou General Hospital Of Thessaloniki); Orsalia Toutouza (Hippocratio General Hospital); Dimitrios Schizas, Christos Doudakmanis, Panagiotis Sakarellos (Laiko University Hospital); Ioannis Katsaros (Metaxa Cancer Hospital); Michael Spartalis (Sotiria General Hospital Of Thoracic Diseases).

India: Anmol Singh, Anand Kothari, Satyajit Sarangi (Post Graduate Institute of Medical Education and Research).

Iraq: Mustafa Al Obaidi (Al-hussien Medical City).

Ireland: Kate O’Shea (St Vincent's University Hospital).

Italy: Stefano Piero Bernardo Cioffi (Asst Grande Ospedale Metropolitano Niguarda); Nicola Passuello, Fabrizio Vittadello, Andrea Grego (Azienda Ospedaliera Di Padova); Riccardo Giuri, Giacomo Faccioli (Azienda Ospedaliera Universitaria Integrata Di Verona); Luigi La Via, Massimiliano Sorbello, Francesco Zagari (Azienda Ospedaliero- Universitaria Policlinico San Marco); Mauro Podda (Cagliari University Hospital); Francesco Velluti (A.O.U. Città della Salute e della Scienza di Torino); Raffaele Vincenzo (Irccs Ospedale Policlinico San Martino); Vincenza Granata (Istituto Nazionale Tumori Fondazione); Domenico Gattulli (Lorenzo Bonomo); Corrado Da Lio (Mirano Hospital); Anton Mariani Ivanikhin (Ospedale Civile Di Voghera); Francesco Roscio (Ospedale Di Circolo Di Busto Arsizio); Lorenzo Maria Fatucchi (Ospedale San Donato Usl Toscana Sud Est); Marco Ceresoli (Ospedale San Gerardo); Andrea Pierre Luzzi, Salvatore Carrabetta, Francesco Floris (Ospedale Villa Scassi); Vincenzo Lizzi, Nicola Tartaglia, Giovanni Di Gioia (Ospedali Riuniti Azienda Ospedaliera Universitaria Foggia); Carmelo Mazzeo, Francesco Fleres (University of Messina, Messina); Juliana Shahu (Santa Annunziata Hospital); Nicola Cillara, Alessandro Cannavera, Francesca D Agostino (Santissima Trinità - Ats Sardegna)

Jordan: Bourhan Alrayes (Islamic Hospital); Ayah Al-Qasrawi (Jordan University Hospital); Suleiman Mahafdah (King Abdullah University Hospital/ Jordan University Of Science And Technology).

Latvia: Pavils Plume, Arturs Truskovs (Pauls Stradins Clinical University Hospital).

Libya: Ebtisam Elbraky, Montaser Benzayed, Mostafa Elawami (Benghazi Medical Center); Hibah Bileid Bakeer, Akram Alkaseek (Gharyan Central Hospital); Mohammed Aljali, Munyah Mohammed (Tobruk Medical Center); Eman Abdulwahed, Reem Ghmagh (Tripoli Central Hospital); Ayyah Abdulfatah Altahir, Asraa Ali Alboaishi (Tripoli Medical Center/ Tripoli University Hospital); Najat Ben Hasan (Zliten Teaching Hospital).

Malaysia: Jie Soang Ooi, Seng Yeong Gan, Edmund Choong Yew Hoe (Hospital Universiti Sains Malaysia); Nora Abdul Aziz (University Malaya Medical Centre).

Mexico: Daniel Lopez Zertuche, Isabel Serrano Trejo (Hospital General Dr. Manuel Gea González); Stefano Minutti Galeazzi, Paola Tellez Castillo (Instituto Nacional De Ciencias Médicas Y Nutrición ‘Salvador Zubirán’).

Morocco: Iltimass Gouazar (Hopital Ibn Tofail); Raouf Mohsine (Institut National D'oncologie).

New Zealand: Jennifer Dang, Abrar Siddiquee (Auckland City Hospital); William Ju (Christchurch Hospital); Nicole Falkner (Middlemore Hospital); Kunj Joshi, Xiao Shen Hu, Calvin Fraser, Caroline Stokowski(North Shore Hospital); Jasmine Seidelin (Tauranga Hospital); Christina Gordon, Sarah Rennie, Anahera Herewini (Wairarapa Hospital); Tom Healy (Wellington Regional Hospital); Luke Paterson (Whangarei Hospital).

Nigeria: Fauziyya Tahir, Mustapha Ibrahim Usman (Aminu Kano Teaching Hospital); Peter Adeoye (University Of Ilorin Teaching Hospital).

Pakistan: Asad Saulat Fatimi (Aga Khan University).

Poland: Hubert Ficner, Wiktor Tworkowski (University Hospital of Karol Marcinkowski in Zielona Góra).

Portugal: Daniela Martins, Catia Ferreira, Goncalo Guidi (Centro Hospitalar De Trás-Os-Montes E Alto Douro); Pedro Silva-Vaz, Manuel Rosete, Cristina Camacho (Centro Hospitalar E Universitário De Coimbra - Hospital Geral); Luisa Frutuoso (Centro Hospitalar Entre O Douro E Vouga); Rita Galama (Centro Hospitalar Médio Tejo); Ana Rita Loureiro (Hospital Das Caldas Da Rainha - Centro Hospitalar Do Oeste); Helena Devesa (Hospital De Santarem); Diana Stoian (Hospital Do Litoral Alentejano); Madalena Trindade (Hospital Garcia De Orta); Ligia Freire (Unidade Local De Saude De Matosinhos - Hospital Pedro Hispano).

Qatar: Ibrahim Amer, Omar Moustafa, Noof Al Naimi (Hamad General Hospital).

Russian Federation: Aleksandr Butyrskii (Municipal Emegency Hospital No.6); Alexander Abelevich (Privolzhsky Research Medical University).

Slovenia: Jurij Ales Kosir, Tajda Kosir Bozic, Jan Grosek (University Medical Centre Ljubljana).

South Africa: Margot Flint, Simphiwe Gumede, Kathryn Nieuwenhuis (Groote Schuur Hospital).

Spain: Francisco Garcia Garcia (Hospital Clínico Universitario De Valencia); Ana Sanchez, Armando Galvan, Enrique Gonzalez (Hospital Del Henares); Beatriz Castro Catalan (Hospital General Universitario Gregorio Marañón); Fernando Mendoza Moreno (Hospital Universitario Principe De Asturias); Ana María Camacho Oliva, Alicia Inmaculada Álvarez González (Hospital Universitario Puerta del Mar); Noelia Ibanez, Angela Alcaraz, Jesus Abrisqueta (Hospital Universitario Virgen De La Arrixaca); Carlos Javier Garcia Sanchez (Hospital Universitario Virgen Del Rocio); Jorge Sancho Muriel, Hanna Cholewa, Monica Millan (Hospital Universitario Y Politécnico La Fe); Aitor Landaluce Olavarria (Hospital Urduliz); Maria Dolores Cancelas Felgueras, Elima Pilar Cagigal Ortega, Francisco Manuel Bujalance Cabrera (Severo Ochoa University Hospital).

Sudan: Mohamed Ahmed, Mohamed Salah, Mohamed Yassin (Gadarif Teaching Hospital); Essam Eldien Abuobaida (Ribat University Hospital).

Switzerland: Sofia El Hajji (Geneva University Hospitals); Lars Korber (Kantonsspital Olten); Jornj Markus Gass, Julia Muhlhausser, Stephanie Strauven (Luzerner Kantonsspital).

Syrian Arab Republic: Mohamad Klib (Al-Mouwasat University Hospital); Zahra Kasem (Aleppo University Hospital).

Turkey: Mert Tanal, Nur Ramoglu (Acibadem Maslak Hospital); Selvi Polat (Dokuz Eylul Univ. Hospital); Banu Yigit (Elazig Fethi Sekin City Hospital); Merve Aslan (Istanbul Medipol University Hospital); Ergin Erginoz, Server Sezgin Uludag, Mehmet Faik Ozcelik (Istanbul Universty - Cerrahpaşa Medical Faculty); Murat Aydemir (Izmir Katip Celebi University Faculty of Medicine); Aras Emre Canda, Mustafa Cem Terzi, Semra Demirli Atici (Izmir Kent Hospital); Hale Cepe (Karadeniz Technical University Farabi Hospital); Muhammed Ikbal Ates, Ibrahim Halil Ozata, Salih Nafiz Karahan, Derya Salim Uymaz, Serkan Sucu, Ahmet Rencuzogullari, Emre Balik, Dilara Yigit, Emre Ozoran, Mekselina Kalender, Arif Emir Narin (Koç University Medical School); Hasan Basri Yapici (Marmara University School of Medicine); Nurhilal Kiziltoprak, Elif Didem Terzi, Haron Cemel (Sultan 2. Abdülhamid Han Research and Training Hospital); Sila Nur Cansiz, Ahmet Hakan Nayman, Buse Selin Acar (Trakya University Hospital); Bulent Citgez (Uskudar University Faculty Of Medicine).

United Kingdom: Da Costa Dorkeh, Amira Orabi (Aberdeen Royal Infirmary); Jeffrey Tooze (Addenbrooke's Hospital); Lauren Mcgill, Matthew Killough (Antrim Area Hospital- Northern Health And Social Care Trust); Mathew Moolamannil (Bedford Hospital); Callum Auld (Belfast City Hospital); Thomas Sullivan (Bristol Royal Infirmary); Rahul Penumaka (Charing Cross Hospital); Kelly De Stadler (Chelsea And Westminster Hospital); Kathryn Allen (Craigavon Area Hospital); Richard Huynh (Darlington Memorial Hospital); Tim Wilson (Doncaster Royal Infirmary); Jadzia Chou (Furness General Hospital); Tanya Premi (Gloucestershire Royal Hospital); Rebecca Badminton (Great Western Hospital); Muhammad Aftab (Guy's And St Thomas' Hospitals); Shahin Zakeri (Hinchingbrooke Hospital); Vasu Sood (Inverclyde Royal Hospital); Nathan Appanna (John Radcliffe Hospital); Anas Saadeh (Lincoln County Hospital); Gopika Nair, Ragul Rajivan (Milton Keynes University Hospital); Eleanor Hopper (Newcastle Upon Tyne Hospitals NHS Foundation Trust); Caitlyn Gallagher, Vithya Vera (Ninewells Hospital); Lahreb Aktar (Norfolk And Norwich University Hospital); Haseeb Syed (North Middlesex University Hospital); Abdulqudus Deeknah (Peterborough City Hospital); Kirtana Ponnuswamy (Queen Elizabeth The Queen Mother Hospital Margate); Nuheel Iqbal (Queens Medical Centre); Alaa Obeida (Rotherham District General Hospital); Grace Lee, Yong Hong Jun, Hua Xuan Yeow (Royal Alexandra Hospital); Ghulam Majeed (Royal Blackburn Hospital); Thomas Smith, Khalid Bukhashem, Laura Munn (Royal Cornwall Hospital); Burraq Imran, Gauri Chillarge (Royal Devon And Exeter Hospital); Tricia Tay (Royal Lancaster Infirmary); Alexander Beneke (Royal London Hospital); Venkata Soumya Bodapati (Scunthorpe General Hospital); Vipanchi Katamaneni (South Warwickshire NHS Foundation Trust); Nika Majidi, Iman Anis (St George's Hospital); Hussain Hassan, Faizan Malik (St James's University Hospital Leeds); Puntrika Tannirandorn (St Mary's Hospital); Katharina Gollub (Stoke Mandeville); Timothy Grommet (University College London Hospital); Amarah Mirza (University Hospitals Of North Midlands); Alessandro Sgro (Victoria Hospital Kirkcaldy); Mahmoud El Khatib (West Middlesex University Hospital); Joanna Kucharczak (West Suffolk Hospital); Zhe Xuan Ho, Shazia Nusky (Western General Hospital).

United States: Matthew Grammer (OSF Saint Francis Medical Center).

## Supplementary Material

zrag062_Supplementary_Data

## Data Availability

Data sharing requests will be considered by the writing group upon written request to the corresponding authors.
