# Correction: Evaluating quality of colorectal cancer surgery across elective and emergency health systems: secondary analysis of prospective cohort studies in 95 countries

**DOI:** 10.1093/bjsopen/zrag200

**Published:** 2026-10-28

This is a correction to: Sivesh K Kamarajah, Adewale O Adisa, Ajay Aggarwal, Alazar Berhe, James Glasbey, Ewen Harrison, Parvez D Haque, Ismail Lawani, Sonia Mathai, Ana Minaya Bravo, C S Pramesh, April C Roslani, Chris Varghese, Aneel Bhangu, on behalf of the NIHR Global Health Research Unit on Global Surgery, Evaluating quality of colorectal cancer surgery across elective and emergency health systems: secondary analysis of prospective cohort studies in 95 countries, *BJS Open*, Volume 10, Issue 4, August 2026, zrag062, https://doi.org/10.1093/bjsopen/zrag062

Instead of the sole affiliation "NIHR Global Health Research Unit on Global Surgery, School of Health Sciences, University of Birmingham, Birmingham, UK", the affiliations list. in content, and affiliations assignment to authors, have been corrected to read:

"Sivesh K Kamarajah^1,*^, Adewale O. Adisa^2^, Ajay Aggarwal^3,4^, Alazar Berhe^5^, James Glasbey^1^, Ewen Harrison^6^, Parvez D Haque^7^, Ismail Lawani^8,^ Sonia Mathai^9^, Ana Minaya Bravo^10^, C S Pramesh^11^, April C Roslani^12^, Chris Varghese^13^, Aneel Bhangu^1,*^, on behalf of the NIHR Global Health Research Unit on Global Surgery^†^

^1^ NIHR Global Health Research Unit on Global Surgery, School of Health Sciences, University of Birmingham, Birmingham, UK

^2^ Obafemi Awolowo University Teaching Hospital Complex, Ife, Osun, Nigeria

^3^ Faculty of Public health and policy, London School of Hygiene and Tropical Medicine, London, UK

^4^ Department of Oncology, Guy’s & ST Thomas’ NHS Trust, London UK

^5^ Hawassa University College of Medicine and Health Sciences, Ethiopia

^6^ Department of HPB Surgery, NHS Lothian, Edinburgh, UK

^7^ Christian Medical College, Ludhiana, India

^8^ International Hospital of Calavi (CHIC), Cotonou, Benin

^9^ Tata Medical Center, Kolkata, India

^10^ General Surgery Department, Hospital Universitario HM Rivas, Rivas-VaciaMadrid, Madrid, Spain

^11^ Tata Memorial Hospital, Homi Bhabha National Institute, Mumbai, India; National Cancer Grid, Tata Memorial Hospital, Mumbai, India

^12^ Faculty of Medicine, Universiti Malaya, Kuala Lumpur, Malaysia; University Malaya Medical Centre, Kuala Lumpur, Malaysia

^13^ Department of Surgery, University of Auckland, Auckland, New Zealand".

